# Numerical simulation data of building integrated solar thermal collectors under diverse conditions

**DOI:** 10.1016/j.dib.2021.107470

**Published:** 2021-10-10

**Authors:** Loucas Georgiou, Manolis Souliotis, Spiros Papaefthimiou, Paris A. Fokaides

**Affiliations:** a School of Engineering, Frederick University, Cyprus; b Department of Chemical Engineering, University of Western Macedonia, Greece; c School of Production Engineering and Management, Technical University of Crete, Greece; d Faculty of Civil Engineering and Architecture, Kaunas University of Technology Lithuania, Lithuania

**Keywords:** Solar collector, Flat plate collector, Building-integrated solar thermal collector, Transient heat transfer, Finite elements

## Abstract

This dataset presents the thermal performance of building-integrated flat solar collectors with a uniform and multiple riser structure. The input data of the numerical model were obtained with the use of the PVGIS tool. Solar radiation and ambient temperature values at slopes 0°, 45°, and 90° were extracted and used as boundary conditions. Numerical calculations were carried using Finite Element (FE) analysis. Three-dimensional transient models were developed to calculate the investigated configurations’ thermal performance based on the environmental temperature, the solar radiation, and the inclination angle. The numerical model was validated with the use of an experimental data set showing a good agreement between the two models with RMSE of 5%. Data of hourly heat flux through the building masonry with the building-integrated solar collector and the average fluid temperature of each system is presented.

## Specifications Table

**Table:** 

Subject	Energy Engineering, Building physics
Specific subject area	Heat transfer, Finite Elements Modelling (FEM), transient heat conduction, heat flux, temperature, Building-Integrated Solar Thermal Collector (BIST)
Type of data	Tables, Figures
How data were acquired	Solar radiation tool (PVGIS) for boundary conditions [2] Finite elements numerical calculation model (Solidworks Flow Simulation) for heat flux and fluid temperature [3]
Data format	Analyzed and processed output data
Parameters for data collection	The geometric parameters of the developed numerical model were considered according to solar collector applications. The solar collector and building materials’ thermophysical properties were acquired from the EN 10456: 2007 [4]. The ambient temperature and solar radiation data obtained using the PVGIS tool [2]
Description of data collection	The thermal medium mass flow rate, which has been used for the solar collector's operation, was obtained from EN 12975-1:2006+A1:2010 [5] The PVGIS tool was employed to acquire climatic data, which was used to define the external boundary conditions of the simulation models. The climatic data extracted was for the calendar months January (winter), April (spring), July (summer), and October (autumn) and for the orientation's azimuth 0°, 90°, 180° and 270°. The heat flux through the building masonry and fluid temperature data was extracted from the FEM tool based on numerical simulation, employing transient heat conduction
Data source location	Nicosia, Cyprus, 35.18° N, 33.37°E
Data accessibility	https://data.mendeley.com/datasets/xcpyjj2gv7/2 https://zenodo.org/record/5284639#.YUHpDS0RpO0

## Value of the Data

• The data provided in this work indicate the impact of design and orientation on the thermal performance of building-integrated solar flat plate collectors.
• The variability of the fluid temperature of flat plate solar collector and heat flux through building the wall under variant external boundary conditions.
• The methodology presented for developing the building-integrated solar flat plate collector can support researchers in optimizing the design for applications, indicating critical parameters.
• The data can also be used as input for numerical models and also be compared to other studies.

## 1 Data Description

**Fig. A1:**
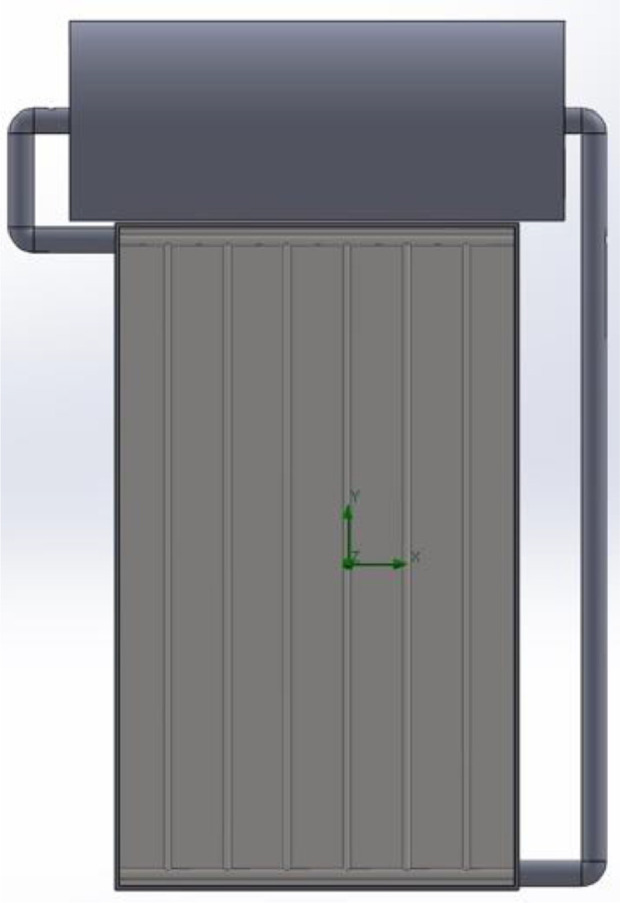
Investigated model of building-integrated solar collector-uniform riser-configuration one.

**Fig. A2:**
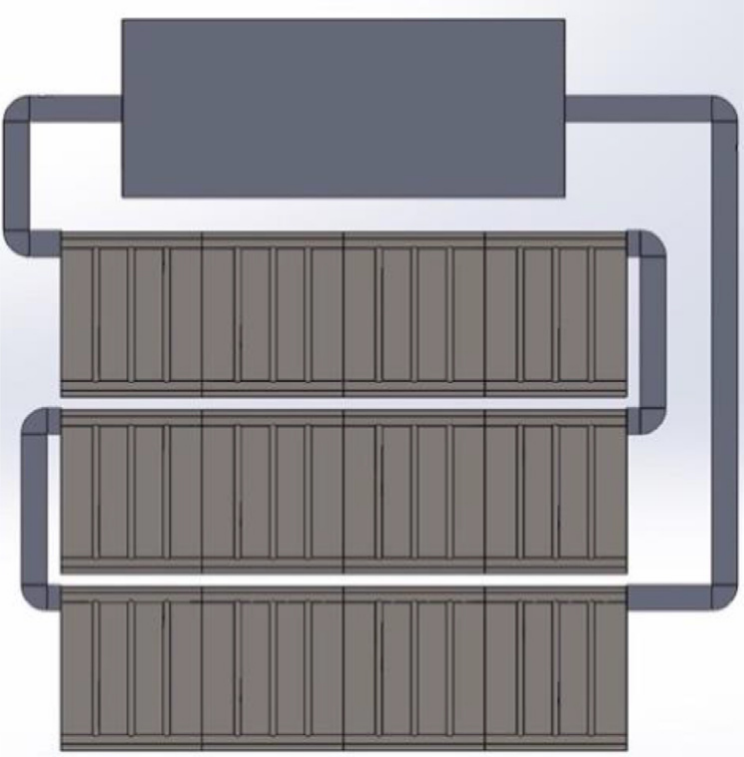
Investigated model of building-integrated solar collector-multiple riser-configuration two.

**Fig. A3:**
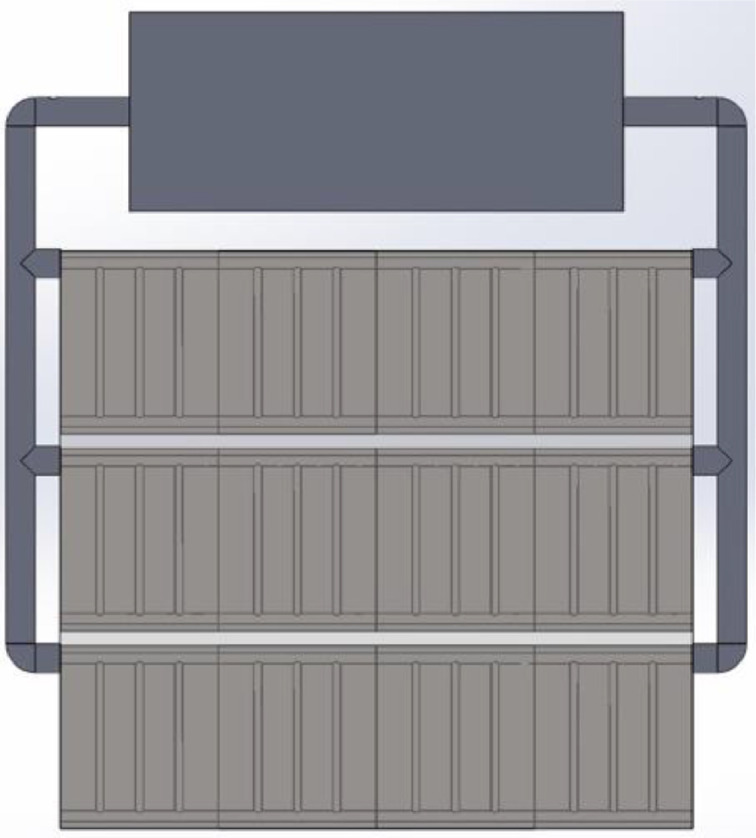
Investigated model of building-integrated solar collector-multiple riser-configuration Three.

**Fig. A4:**
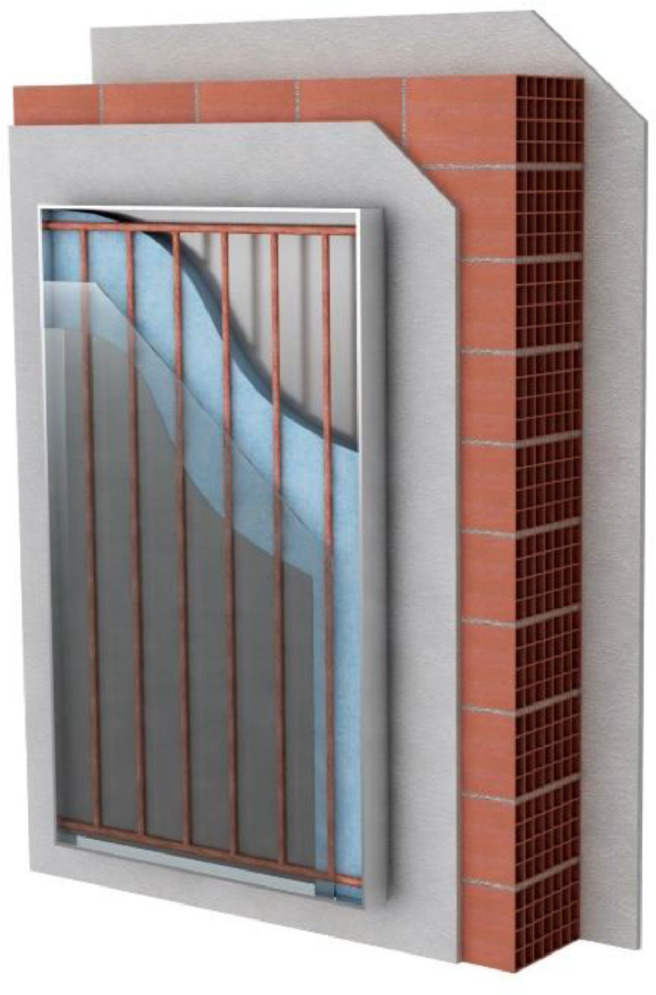
Building-integrated solar collector structure.

A summary overview of the numerical results is presented in tables (Table 1, Table 2, Table 3). Reference figures (Figs. A1–A4) demonstrate the three configurations investigated and the building-integrated setup configuration. The data provided in Table 1, Table 2, Table 3 present the minimum, average and maximum fluid system temperature achieved by configurations one, two and three, respectively. The values of fluid temperature are distinguished from the hourly value data according to the minimum, average, and maximum criteria for each orientation.

**Table 1:** Minimum, average and maximum-system fluid temperature [C°]-all configurations-all seasons-slope 0°.

	Uniform Riser-Configuration One			Multiple Riser-Configuration Two			Multiple Riser-Configuration Three		
	Min	Average	Max	Min	Average	Max	Min	Average	Max
Autumn	21.09	22.37	23.84	24.87	28.88	33.74	23.36	26.22	29.69
Winter	14.72	16.38	18.26	15.60	17.87	20.43	15.60	17.84	20.39
Spring	21.86	25.93	30.72	24.17	29.93	36.78	24.19	29.91	36.73
Summer	35.07	40.15	45.91	37.96	45.47	54.00	38.00	45.46	53.90

**Table 2:** Minimum, average and maximum-system fluid temperature [C°]-all configurations-all seasons-slope 45°.

		Uniform Riser-Configuration One			Multiple Riser-Configuration Two			Multiple Riser-Configuration Three		
		Min	Average	Max	Min	Average	Max	Min	Average	Max
Autumn	South	25.59	30.05	35.25	27.97	34.36	42.07	27.99	34.36	41.98
	West	23.08	25.60	28.96	24.52	28.03	32.67	24.51	28.01	32.63
	North	20.67	21.65	22.80	21.09	22.37	23.84	21.08	22.36	23.83
	East	22.79	25.65	28.56	24.04	28.05	32.20	24.05	28.04	32.16

Winter	South	15.80	18.15	20.84	17.10	20.38	24.13	17.09	20.33	24.04
	West	14.59	16.04	17.84	15.42	17.39	19.82	15.41	17.36	19.77
	North	13.53	14.39	15.33	13.94	15.06	16.28	13.93	15.04	16.27
	East	14.50	16.11	17.77	15.25	17.47	19.75	15.26	17.44	19.70

Spring	South	23.00	27.78	33.46	25.71	32.63	40.91	25.75	32.61	40.85
	West	21.30	24.80	29.45	23.42	28.36	34.91	23.40	28.34	34.97
	North	18.17	19.90	21.88	19.08	21.38	24.01	19.09	21.37	23.97
	East	20.89	24.85	28.84	22.80	28.38	34.09	22.81	28.38	34.03

Summer	South	34.46	39.49	45.21	37.19	44.53	52.96	37.25	44.51	52.92
	West	34.33	38.52	44.26	37.03	43.18	51.55	37.03	43.15	51.54
	North	32.48	35.65	39.10	34.48	38.97	43.88	34.49	38.94	43.82
	East	33.62	38.79	43.73	36.02	43.51	50.88	36.01	43.49	50.80

**Table 3:** Minimum, average and maximum-system fluid temperature [C°]-all configurations-all seasons-slope 90°.

		Uniform Riser-Configuration One			Multiple Riser-Configuration Two			Multiple Riser-Configuration Three		
		Min	Average	Max	Min	Average	Max	Min	Average	Max
Autumn	South	25.00	28.98	33.61	27.12	32.89	39.74	27.16	32.81	39.50
	West	22.03	23.78	26.22	23.02	25.44	28.80	23.00	25.40	28.71
	North	20.20	20.90	21.71	20.44	21.32	22.32	20.43	21.30	22.29
	East	21.71	23.82	25.78	22.55	25.48	28.23	22.57	25.43	28.14

Winter	South	15.45	17.54	19.91	16.59	19.52	22.84	16.60	19.46	22.71
	West	13.98	15.01	16.33	14.56	15.94	17.68	14.55	15.91	17.63
	North	13.12	13.66	14.24	13.33	14.04	14.79	13.32	14.03	14.77
	East	13.83	15.00	16.12	14.34	15.94	17.46	14.35	15.90	17.40

Spring	South	20.92	24.42	28.52	22.89	27.83	33.67	21.95	26.59	32.21
	West	19.55	21.85	25.16	20.98	24.19	28.77	19.78	22.91	27.37
	North	16.81	17.75	18.80	17.17	18.34	19.66	16.15	17.09	18.28
	East	18.94	21.76	24.21	20.15	24.10	27.57	19.13	22.82	26.13

Summer	South	30.81	34.06	37.67	32.39	36.27	40.70	32.40	36.21	40.58
	West	31.97	35.19	40.18	33.81	37.83	43.86	33.79	37.76	43.63
	North	28.91	30.12	31.37	29.52	30.88	32.30	29.53	30.85	32.25
	East	31.04	35.22	38.98	32.74	37.90	42.58	32.74	37.83	42.42

The figures and tables (Reference Figs. B1–B16, Tables A1–A17, Figs. B1–B3) presented in supplementary material provide brief analytical data. The Reference Figures B1, B3, B5, illustrate the investigated configurations, and Reference Figures (Figs. B2, B4, B6) present the domain meshes of the investigated geometries.

The Reference Figures (B7, B9, B11) present each configuration's riser geometry and Reference Figures (Figs. B8, B10, B12) the fluid flow pattern of each numerical model. Reference Fig. B13 shows the integration of the building-integrated solar collector structure. Reference figures (Figs. B14–B16) illustrate the riser fluid velocity distribution for each numerical model system. Tables A1-A9 provide the overall hourly average fluid temperature of each numerical model. Table A10 shows the hourly average heat flux through the building masonry without BIST integration, and Tables A11–A13 show the hourly heat flux values through the building masonry with BIST integration. The solar radiation values used as boundary conditions in the numerical model are provided in Tables A14–A16 and the ambient temperature in Tables A17. The datasets are sorted according to autumn, winter, spring, and summer conditions under the slopes 0°, 45°,90° for north, South, West, and East orientations. A graphical comparison of the three numerical models is provided in Figs. B1–B3, indicating the difference in fluid temperature according to the orientation. All the files provided in the Mendeley data are for reproduction purposes, with all the values accessible for edit [1].

## 2 Experimental Design, Materials and Methods

The calculation procedure, was based on a three-dimensional time-dependent finite element numerical modelling. A geometrical model of a building integrated solar collector with various riser configurations was developed. The thermophysical properties of the materials assumed were retrieved from the international standards EN 10456:2007 [4] and EN 12975-1:2006+A1:2010 [5]. The time step of the analysis ranged from 0,1, to 24 [h]. The internal wall of the building was set as an open boundary, whereas for the external boundary, the T-sol temperature was assumed. The boundary conditions of the numerical analysis are summarized in Table 4. These properties are summarized in Table 6. As far as the ambient conditions are concerned (temperature, solar radiation), they were defined with the use of the PVGIS tool [2]. The data was processed for different seasons, orientations and slopes (see Table 6). The simulation was performed for all four seasons of the year (winter, spring, summer autumn) and for all four main orientations of the building (north, east, south, west) for a solar collector slope of 90°. Simulations were also performed for the roof for slopes of 0° and south facing 45°.

**Table 4:** Boundary conditions.

Property	Value	Units
Thermal medium mass flow rate	0.038	[kg/s]
Solar thermal radiation	Transient (Supplementary Data)	[W/m²]
Ambient Temperature	Transient (Supplementary Data)	[°C]
Water initial temperature	Regulated (24 hours initial operation)	[°C]
Inclination	0,45,90	°

**Table 5:** Thermophysical properties of the materials used as input in the numerical simulation study of novel double-skin façade (DSF) controlled- temperature building element.

Material	Density [kg/m^3^]	Thermal Conductivity [W/(m·K)]	Heat Capacity [J(Kg·K)]	Thickness [cm]
Masonry Wall				

Mortar Plaster	700	1.000	1000	2.50
Hollow Brick (Clay Material)	880	0.400	900	20.00
Brick (Air Holes 5 × 5 [cm])	1.23	0.025	1008	20.00

Solar Collector				

Glass	2457.6	0.74976	834.61	0.20
Aluminium	2700	200	900	10.00
Cooper	8900	390	390	0.09
Polyurethane	70	0.05	1500	2.00
Mineral Wool	70	0.035	837	5.00

**Table 6:** Building-integrated solar collector materials.

Solar Collector		Masonry Wall	
Component Description	Material	Component Description	Material
Front case cover	Glass	Exterior	Plaster
Case	Aluminium	Interior	Plaster
Riser	Cooper	Brick	Hollow Brick
Pipes	Cooper		
Pipes Insulation	Polyurethane		
Tank	Cooper		
Tank Insulation	Mineral Wool		
Case insulation	Mineral Wool		

The governing equations employed were the mass, momentum and energy conservation laws, based on the Navier-Strokes approach, for closed-loop forced circulation, expressed as follows:

(1) $$\frac{\partial \rho }{\partial t}+\frac{\partial \left(\rho u_{i}\right)}{\partial x_{i}}=\;0$$

(2) $$\frac{\partial \left(\rho u_{i}\right)}{\partial t}+\frac{\partial }{\partial x_{j}}\left(\rho u_{i}u_{j}\right)+\frac{\partial P}{\partial x_{i}}=\frac{\partial }{\partial x_{j}}\left(\tau_{ij}+\tau_{ij}^{R}\right)+S_{i}$$

(3) $$\frac{\partial \rho H}{\partial t}+\frac{\partial \rho u_{i}H}{\partial x_{i}}=\frac{\partial }{\partial x_{i}}\left(u_{j}\left(\tau_{ij}+\tau_{ij}^{R}\right)+q_{i}\right)+\frac{\partial \rho }{\partial t}-\tau_{ij}^{R}\frac{\partial u_{i}}{\partial x_{j}}+\rho \varepsilon +S_{i}u_{i}+Q_{H}$$

(4) $$H=h+\frac{u^{2}}{2}$$

Concerning the solid regions of the model, heat conduction was assumed:

(5) $$\frac{\partial \rho e}{\partial t}=\frac{\partial }{\partial x_{i}}\left(\lambda_{i}\frac{\partial T}{\partial x_{i}}\right)\;+Q_{H}$$

### 2.1 Numerical model validation

The validation of the numerical model employed in this study was implemented with the use of experimental data published by Souliotis [6]. Particularly the geometry described in [6] was developed and the boundary conditions, as well as the physics of the implemented numerical model were applied. The validation of the experimental (E) and numerical values (N) was incorporated by the use of the root mean square deviation (RMSD) formula.

(6) $$RMSD=\sqrt{\frac{\sum_{i=1\;}^{n}{\left(N_{i}-E_{i}\right)}^{2}}{n}}$$

**Fig. 5:**
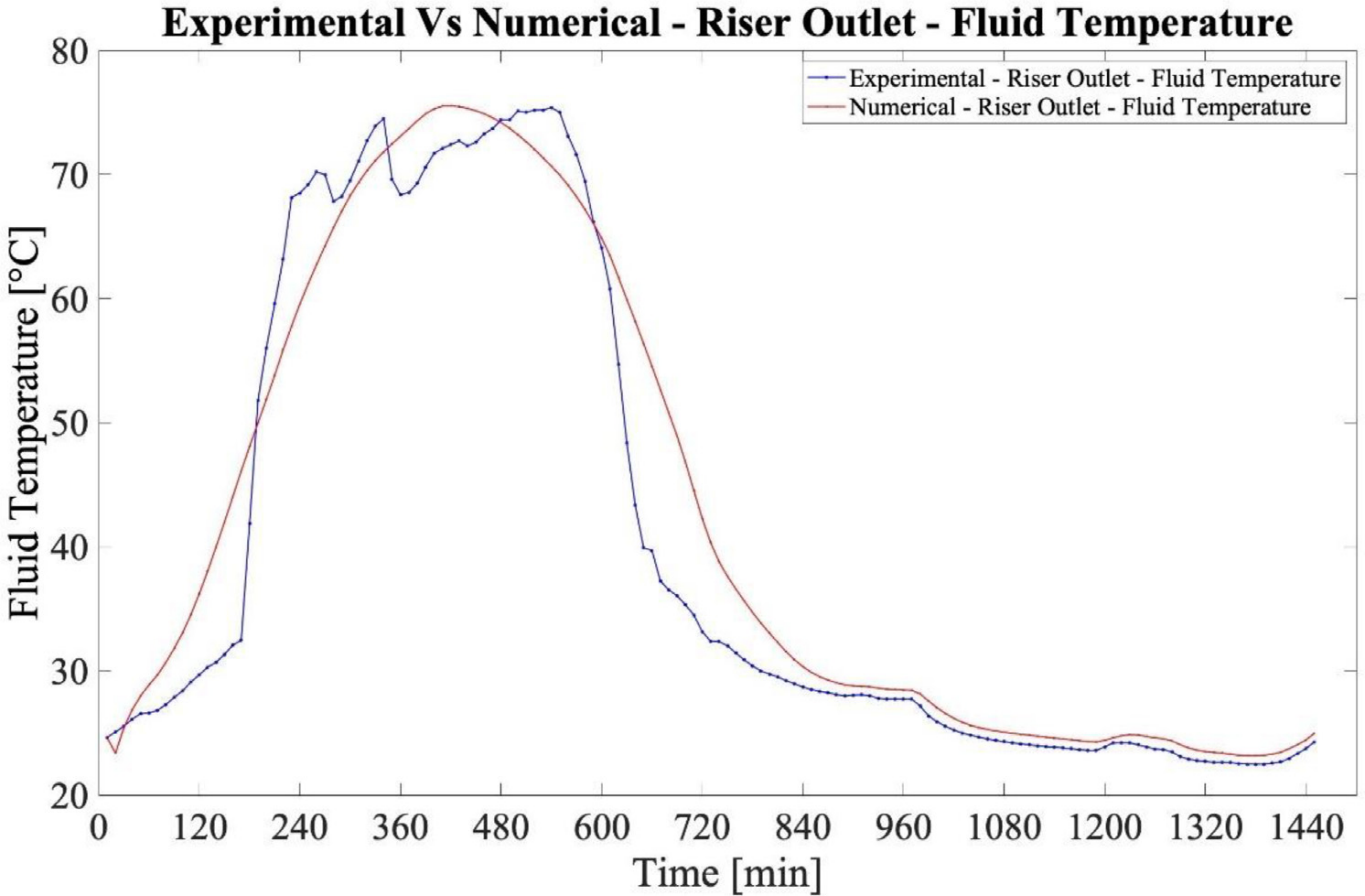
Numerical model validation-riser outlet-fluid temperature.

In Fig. 5, the agreement between the experimental and numerical values is presented. As calculated from the obtained values of experimental and numerical cases, the RMSD is 5.01%, a value which is considered satisfactory [7].

## Ethics Statement

No ethical issues are associated with this work.

## CRediT authorship contribution statement

**Loucas Georgiou:** Writing – original draft, Writing – review & editing, Formal analysis, Investigation. **Manolis Souliotis:** Writing – review & editing. **Spiros Papaefthimiou:** Writing – review & editing. **Paris A. Fokaides:** Conceptualization, Methodology, Validation, Resources, Visualization, Supervision, Project administration.

## Declaration of Competing Interest

The authors declare that they have no known competing financial interests or personal relationships which have, or could be perceived to have, influenced the work reported in this article.

## Appendix Supplementary materials

Image, application 1
