# Supplementary material for: Numerical simulation data of building integrated solar thermal collectors under diverse conditions
Source: Data Brief. 2021 Oct 10;39:107470. doi: 10.1016/j.dib.2021.107470 (PMC8529080; doi:10.1016/j.dib.2021.107470)
Supplement: Supplementary file 1 [file mmc1.docx]

| 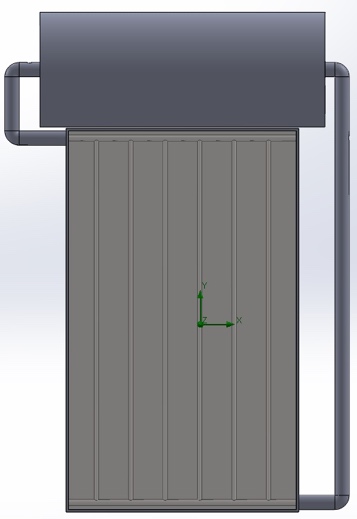 | 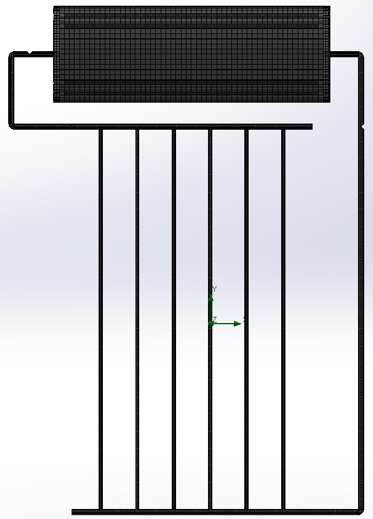 |
| --- | --- |
| **Reference Figure B1.** Investigated model of building-integrated solar collector – Uniform Riser – Configuration One | **Reference Figure B2.** Investigated model of building-integrated solar collector – Uniform Riser – Configuration two |

| 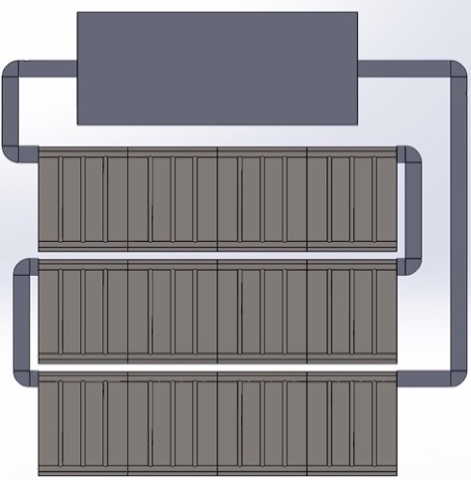 | 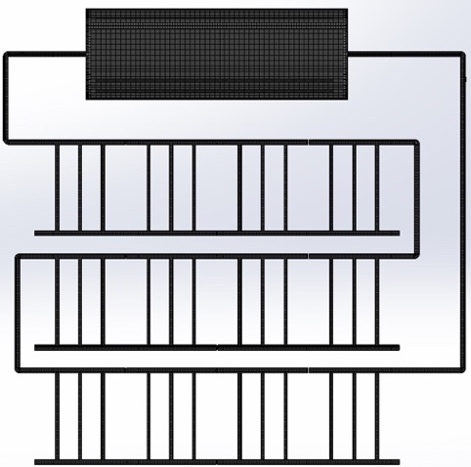 |
| --- | --- |
| **Reference Figure B3.** Investigated model of building-integrated solar collector – Multiple Riser - Configuration One | **Reference Figure B4.** Investigated domain mesh of configuration two |

| 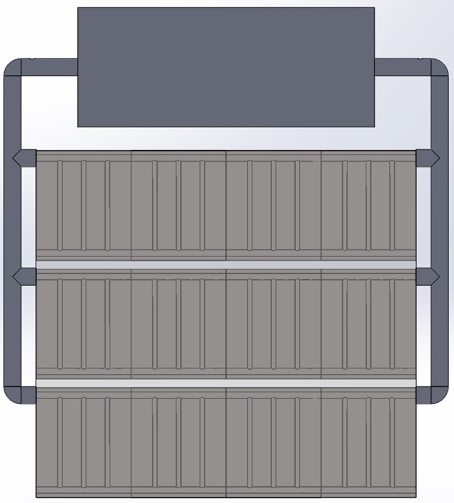 | 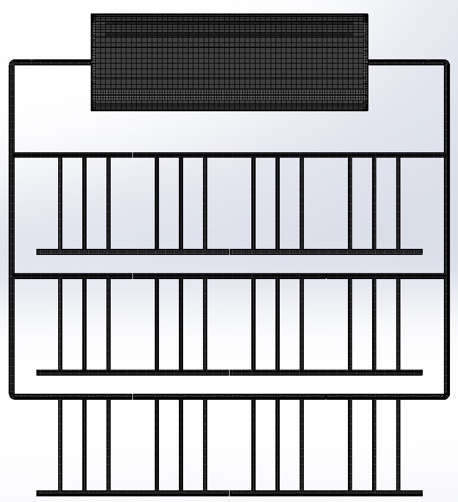 |
| --- | --- |
| **Reference Figure B5.** Investigated model of building-integrated solar collector – Multiple Riser - Configuration Two | **Reference Figure B6.** Investigated domain mesh of configuration three |

| **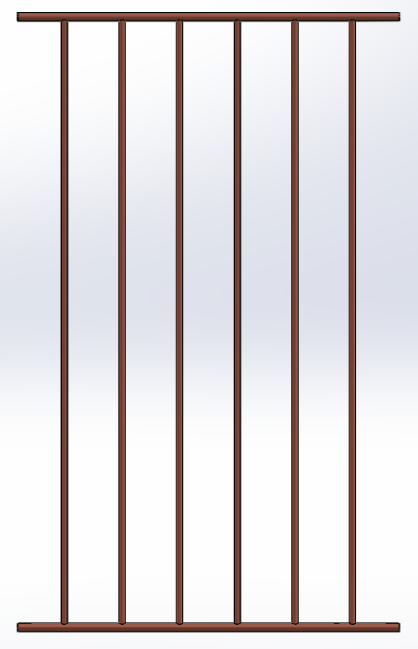** | 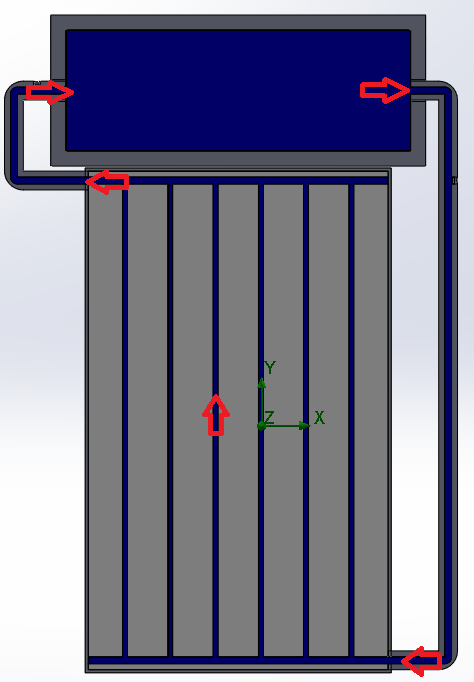 |
| --- | --- |
| **Reference Figure B7.** Investigated riser configuration one of building-integrated solar collector | **Reference Figure B8.** Investigated fluid flow of configuration one |

| **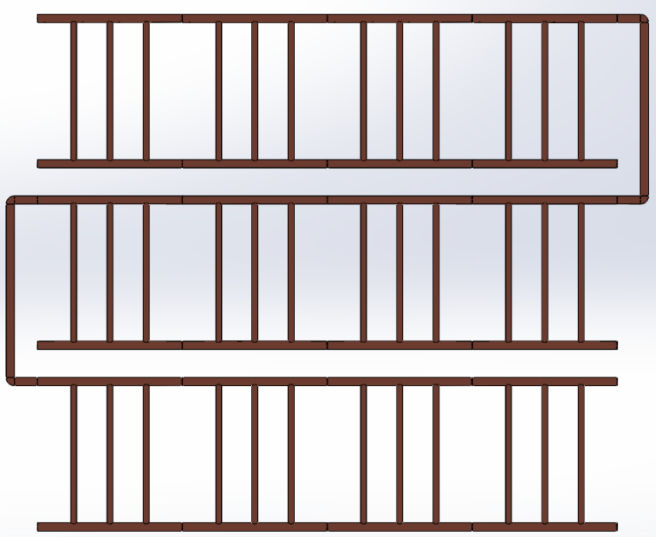** | 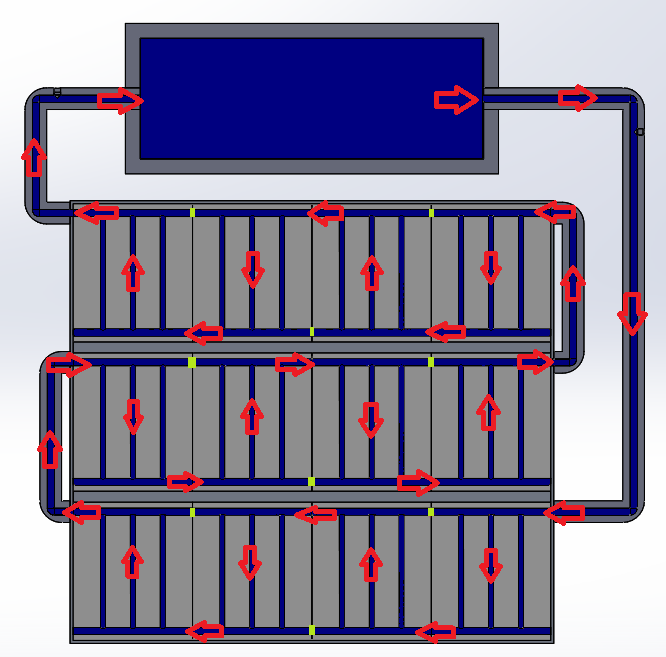 |
| --- | --- |
| **Reference Figure B9.** Investigated riser configuration two of building-integrated solar collector | **Reference Figure B10.** Investigated fluid flow of configuration two |

| **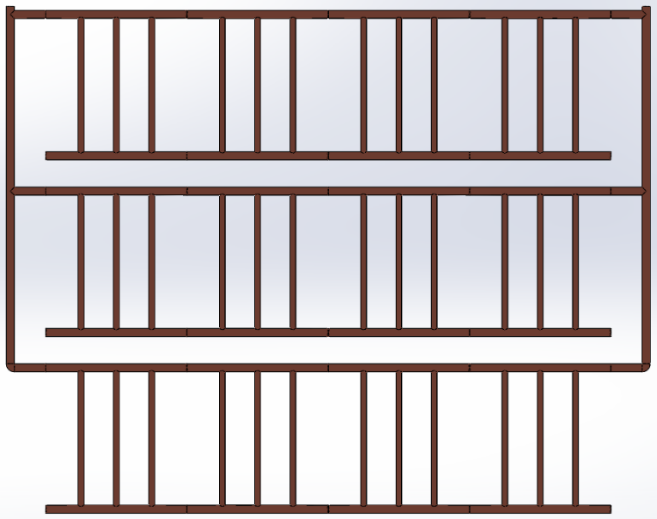** | 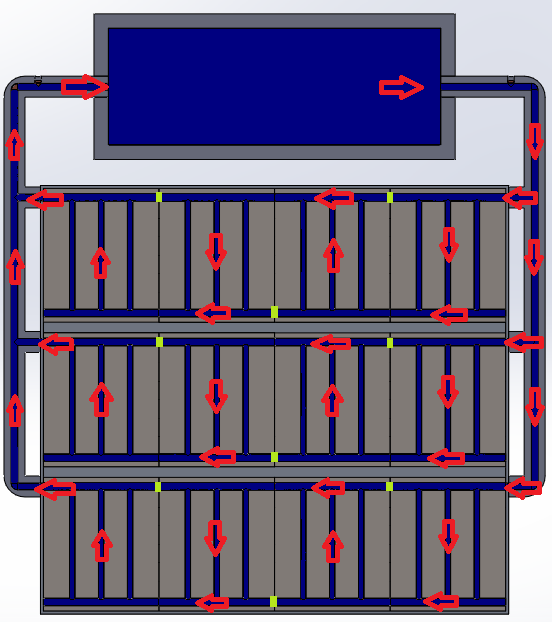 |
| --- | --- |
| **Reference Figure B11.** Investigated riser configuration three of building-integrated solar collector | **Reference Figure B12.** Investigated fluid flow of configuration three |

| 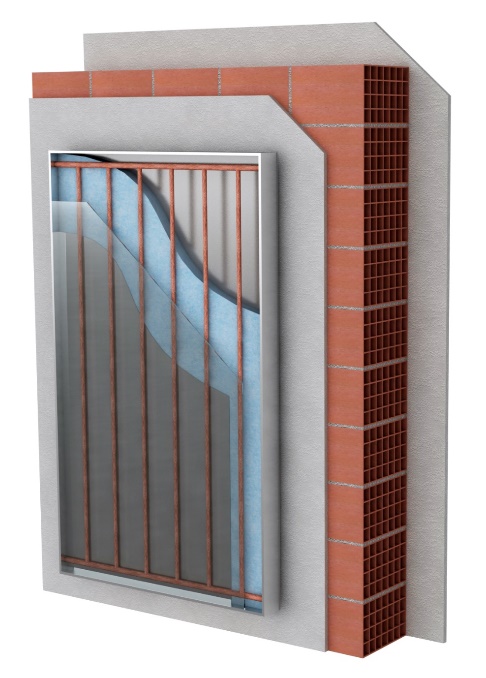 | 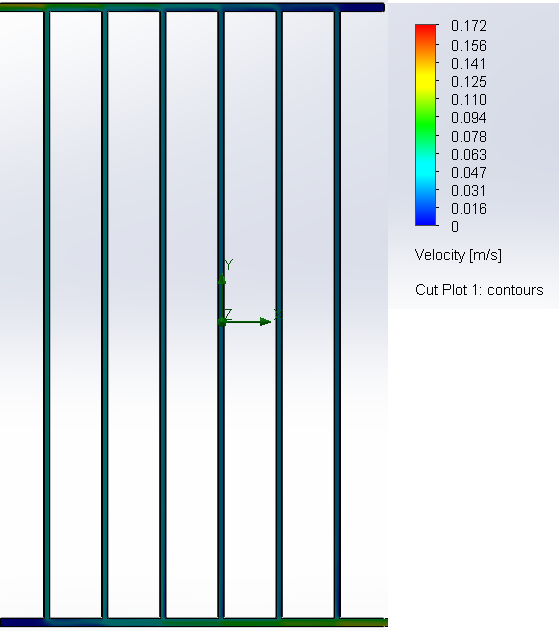 |
| --- | --- |
| **Reference Figure B13.** Building-integrated solar collector structure | **Reference Figure B14.** Fluid flow riser velocity of investigated configuration one |

| 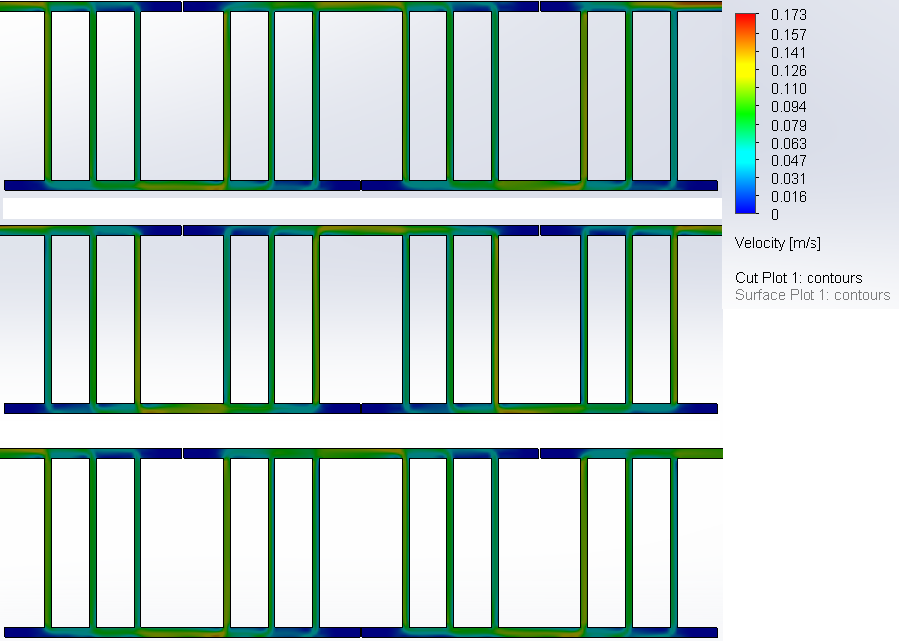 |
| --- |
| **Reference Figure B15.** Fluid flow riser velocity of investigated configuration two |

| 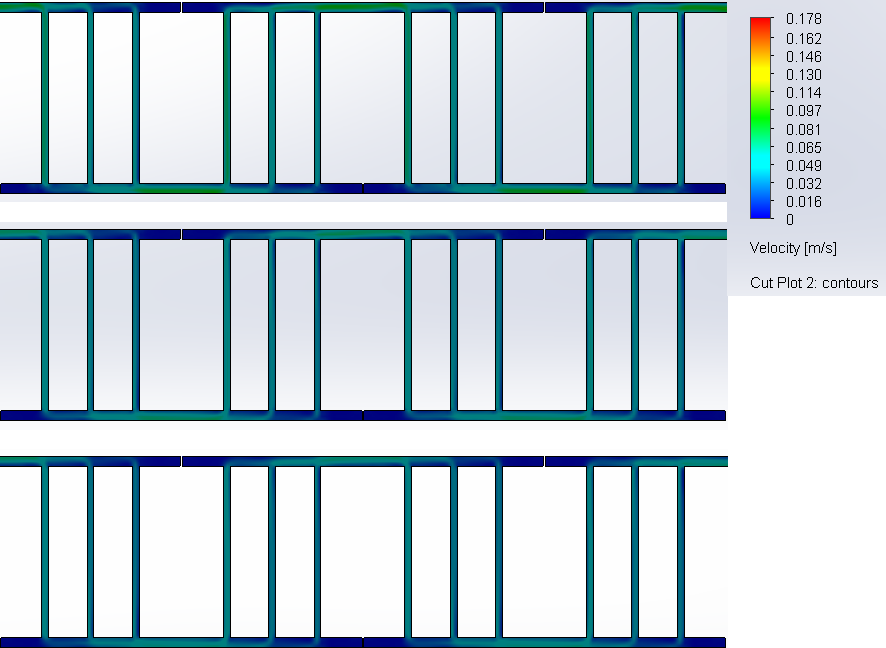 |
| --- |
| **Reference Figure B16.** Fluid flow riser velocity of investigated configuration three |

## **A.1 Uniform Riser - Configuration One –Fluid Average Temperature, Slope 0° - Reference Figure B1**

**Table 1** Fluid Average Temperature [$^{\circ}C$] Versus All Months - Uniform Riser – Configuration One - Slope 0°

| **Hour** | **November** | **January** | **March** | **August** |
| --- | --- | --- | --- | --- |
| **00:00** | 21.63 | 15.52 | 23.53 | 36.84 |
| **01:00** | 21.48 | 15.33 | 23.04 | 36.23 |
| **02:00** | 21.33 | 15.15 | 22.58 | 35.65 |
| **03:00** | 21.20 | 14.98 | 22.16 | 35.19 |
| **04:00** | 21.10 | 14.82 | 21.86 | 35.07 |
| **05:00** | 21.09 | 14.72 | 21.86 | 35.42 |
| **06:00** | 21.20 | 14.78 | 22.22 | 36.23 |
| **07:00** | 21.43 | 15.03 | 22.94 | 37.41 |
| **08:00** | 21.77 | 15.48 | 24.02 | 38.81 |
| **09:00** | 22.19 | 16.07 | 25.39 | 40.44 |
| **10:00** | 22.65 | 16.71 | 26.87 | 42.04 |
| **11:00** | 23.13 | 17.32 | 28.28 | 43.46 |
| **12:00** | 23.52 | 17.83 | 29.47 | 44.65 |
| **13:00** | 23.77 | 18.15 | 30.33 | 45.54 |
| **14:00** | 23.84 | 18.26 | 30.72 | 45.91 |
| **15:00** | 23.78 | 18.19 | 30.62 | 45.73 |
| **16:00** | 23.64 | 18.02 | 30.17 | 45.08 |
| **17:00** | 23.44 | 17.75 | 29.44 | 44.09 |
| **18:00** | 23.17 | 17.39 | 28.46 | 42.80 |
| **19:00** | 22.85 | 17.00 | 27.41 | 41.50 |
| **20:00** | 22.55 | 16.63 | 26.44 | 40.36 |
| **21:00** | 22.27 | 16.29 | 25.56 | 39.27 |
| **22:00** | 22.01 | 15.99 | 24.76 | 38.30 |
| **23:00** | 21.80 | 15.74 | 24.10 | 37.49 |

## **A.2 Multiple Riser - Configuration Two – Fluid Average Temperature, Slope 0°- Reference Figure B3**

**Table 2** Fluid Average Temperature [$^{\circ}C$] Versus All Months - Multiple Riser - Configuration Two - Slope 0°

| **Hour** | **November** | **January** | **March** | **August** |
| --- | --- | --- | --- | --- |
| **00:00** | 26.97 | 16.74 | 26.56 | 40.66 |
| **01:00** | 26.49 | 16.47 | 25.87 | 39.75 |
| **02:00** | 26.06 | 16.22 | 25.23 | 38.90 |
| **03:00** | 25.65 | 15.98 | 24.63 | 38.21 |
| **04:00** | 25.26 | 15.75 | 24.21 | 37.96 |
| **05:00** | 24.95 | 15.60 | 24.17 | 38.37 |
| **06:00** | 24.87 | 15.64 | 24.66 | 39.50 |
| **07:00** | 25.17 | 15.95 | 25.66 | 41.18 |
| **08:00** | 25.98 | 16.51 | 27.20 | 43.28 |
| **09:00** | 27.28 | 17.30 | 29.20 | 45.75 |
| **10:00** | 28.86 | 18.19 | 31.39 | 48.22 |
| **11:00** | 30.63 | 19.09 | 33.62 | 50.62 |
| **12:00** | 32.13 | 19.80 | 35.25 | 52.35 |
| **13:00** | 33.21 | 20.26 | 36.36 | 53.53 |
| **14:00** | 33.74 | 20.43 | 36.78 | 54.00 |
| **15:00** | 33.67 | 20.36 | 36.52 | 53.70 |
| **16:00** | 33.23 | 20.13 | 35.77 | 52.71 |
| **17:00** | 32.54 | 19.78 | 34.70 | 51.27 |
| **18:00** | 31.63 | 19.31 | 33.32 | 49.46 |
| **19:00** | 30.63 | 18.77 | 31.85 | 47.55 |
| **20:00** | 29.71 | 18.26 | 30.51 | 45.80 |
| **21:00** | 28.87 | 17.80 | 29.29 | 44.22 |
| **22:00** | 28.11 | 17.38 | 28.21 | 42.81 |
| **23:00** | 27.48 | 17.03 | 27.30 | 41.63 |

## **A.3 Multiple Riser - Configuration Three – Fluid Average Temperature, Slope 0°- Reference Figure B5**

**Table 3** Fluid Average Temperature [$^{\circ}C$] Versus All Months - Multiple Riser - Configuration Three - Slope 0°

| **Hour** | **November** | **January** | **March** | **August** |
| --- | --- | --- | --- | --- |
| **00:00** | 24.80 | 16.73 | 26.58 | 40.66 |
| **01:00** | 24.47 | 16.47 | 25.90 | 39.77 |
| **02:00** | 24.16 | 16.22 | 25.26 | 38.93 |
| **03:00** | 23.88 | 15.98 | 24.67 | 38.24 |
| **04:00** | 23.60 | 15.76 | 24.23 | 38.00 |
| **05:00** | 23.39 | 15.60 | 24.19 | 38.42 |
| **06:00** | 23.36 | 15.64 | 24.66 | 39.54 |
| **07:00** | 23.63 | 15.94 | 25.64 | 41.19 |
| **08:00** | 24.24 | 16.49 | 27.15 | 43.28 |
| **09:00** | 25.17 | 17.28 | 29.16 | 45.74 |
| **10:00** | 26.28 | 18.16 | 31.33 | 48.19 |
| **11:00** | 27.44 | 19.05 | 33.49 | 50.43 |
| **12:00** | 28.48 | 19.76 | 35.15 | 52.19 |
| **13:00** | 29.28 | 20.22 | 36.28 | 53.40 |
| **14:00** | 29.69 | 20.39 | 36.73 | 53.90 |
| **15:00** | 29.65 | 20.31 | 36.49 | 53.64 |
| **16:00** | 29.36 | 20.08 | 35.75 | 52.68 |
| **17:00** | 28.86 | 19.73 | 34.66 | 51.24 |
| **18:00** | 28.20 | 19.26 | 33.30 | 49.44 |
| **19:00** | 27.47 | 18.73 | 31.83 | 47.53 |
| **20:00** | 26.81 | 18.23 | 30.50 | 45.80 |
| **21:00** | 26.19 | 17.77 | 29.30 | 44.23 |
| **22:00** | 25.63 | 17.36 | 28.23 | 42.83 |
| **23:00** | 25.17 | 17.02 | 27.33 | 41.66 |

## **A.4 Uniform Riser - Configuration One – Fluid Average Temperature, Slope 45°- Reference Figure B1**

**Table 4** Fluid Average Temperature [$^{\circ}C$] Versus Orientation - Uniform Riser – Configuration One – November - Slope 45°

| **Hour** | **North** | **South** | **West** | **East** |
| --- | --- | --- | --- | --- |
| **00:00** | 21.06 | 27.22 | 24.41 | 23.58 |
| **01:00** | 20.95 | 26.74 | 24.10 | 23.32 |
| **02:00** | 20.84 | 26.30 | 23.81 | 23.07 |
| **03:00** | 20.74 | 25.89 | 23.54 | 22.84 |
| **04:00** | 20.67 | 25.59 | 23.31 | 22.79 |
| **05:00** | 20.67 | 25.65 | 23.16 | 23.14 |
| **06:00** | 20.77 | 26.19 | 23.08 | 23.92 |
| **07:00** | 20.95 | 27.20 | 23.12 | 25.03 |
| **08:00** | 21.22 | 28.55 | 23.43 | 26.16 |
| **09:00** | 21.56 | 30.15 | 24.10 | 27.18 |
| **10:00** | 21.91 | 31.86 | 25.09 | 27.95 |
| **11:00** | 22.26 | 33.40 | 26.29 | 28.38 |
| **12:00** | 22.56 | 34.57 | 27.49 | 28.56 |
| **13:00** | 22.75 | 35.22 | 28.48 | 28.52 |
| **14:00** | 22.80 | 35.25 | 28.96 | 28.30 |
| **15:00** | 22.75 | 34.84 | 28.89 | 27.95 |
| **16:00** | 22.63 | 34.18 | 28.61 | 27.52 |
| **17:00** | 22.47 | 33.32 | 28.14 | 27.01 |
| **18:00** | 22.24 | 32.26 | 27.52 | 26.42 |
| **19:00** | 22.00 | 31.16 | 26.86 | 25.80 |
| **20:00** | 21.77 | 30.16 | 26.27 | 25.24 |
| **21:00** | 21.55 | 29.25 | 25.70 | 24.73 |
| **22:00** | 21.36 | 28.44 | 25.19 | 24.27 |
| **23:00** | 21.19 | 27.77 | 24.77 | 23.90 |

**Table 5** Fluid Average Temperature [$^{\circ}C$] Versus Orientation - Uniform Riser - Configuration One – January - Slope 45°

| **Hour** | **North** | **South** | **West** | **East** |
| --- | --- | --- | --- | --- |
| **00:00** | 13.98 | 16.89 | 15.46 | 15.13 |
| **01:00** | 13.88 | 16.63 | 15.28 | 14.96 |
| **02:00** | 13.78 | 16.38 | 15.10 | 14.80 |
| **03:00** | 13.68 | 16.14 | 14.94 | 14.65 |
| **04:00** | 13.59 | 15.92 | 14.78 | 14.51 |
| **05:00** | 13.53 | 15.80 | 14.65 | 14.50 |
| **06:00** | 13.54 | 15.94 | 14.59 | 14.76 |
| **07:00** | 13.65 | 16.35 | 14.61 | 15.23 |
| **08:00** | 13.87 | 16.98 | 14.79 | 15.82 |
| **09:00** | 14.17 | 17.79 | 15.16 | 16.44 |
| **10:00** | 14.51 | 18.65 | 15.67 | 17.00 |
| **11:00** | 14.82 | 19.48 | 16.28 | 17.40 |
| **12:00** | 15.08 | 20.18 | 16.91 | 17.65 |
| **13:00** | 15.26 | 20.66 | 17.45 | 17.77 |
| **14:00** | 15.33 | 20.84 | 17.78 | 17.74 |
| **15:00** | 15.31 | 20.74 | 17.84 | 17.60 |
| **16:00** | 15.24 | 20.48 | 17.75 | 17.40 |
| **17:00** | 15.11 | 20.09 | 17.55 | 17.13 |
| **18:00** | 14.94 | 19.57 | 17.24 | 16.80 |
| **19:00** | 14.75 | 19.00 | 16.88 | 16.45 |
| **20:00** | 14.56 | 18.47 | 16.53 | 16.11 |
| **21:00** | 14.38 | 17.98 | 16.21 | 15.81 |
| **22:00** | 14.23 | 17.55 | 15.92 | 15.54 |
| **23:00** | 14.10 | 17.19 | 15.68 | 15.32 |

**Table 6** Fluid Average Temperature [$^{\circ}C$] Versus Orientation - Uniform Riser – Configuration One – March - Slope 45°

| **Hour** | **North** | **South** | **West** | **East** |
| --- | --- | --- | --- | --- |
| **00:00** | 18.93 | 24.94 | 23.46 | 22.02 |
| **01:00** | 18.71 | 24.37 | 22.98 | 21.61 |
| **02:00** | 18.50 | 23.85 | 22.53 | 21.24 |
| **03:00** | 18.30 | 23.36 | 22.11 | 20.92 |
| **04:00** | 18.17 | 23.00 | 21.75 | 20.89 |
| **05:00** | 18.18 | 23.00 | 21.49 | 21.40 |
| **06:00** | 18.33 | 23.43 | 21.33 | 22.38 |
| **07:00** | 18.63 | 24.27 | 21.30 | 23.71 |
| **08:00** | 19.07 | 25.54 | 21.59 | 25.13 |
| **09:00** | 19.62 | 27.16 | 22.29 | 26.53 |
| **10:00** | 20.20 | 28.93 | 23.39 | 27.69 |
| **11:00** | 20.76 | 30.60 | 24.77 | 28.44 |
| **12:00** | 21.25 | 32.00 | 26.27 | 28.78 |
| **13:00** | 21.63 | 33.01 | 27.80 | 28.84 |
| **14:00** | 21.85 | 33.46 | 28.94 | 28.67 |
| **15:00** | 21.88 | 33.31 | 29.45 | 28.29 |
| **16:00** | 21.75 | 32.76 | 29.35 | 27.74 |
| **17:00** | 21.48 | 31.88 | 28.85 | 27.04 |
| **18:00** | 21.09 | 30.73 | 28.04 | 26.18 |
| **19:00** | 20.65 | 29.49 | 27.11 | 25.27 |
| **20:00** | 20.22 | 28.35 | 26.26 | 24.44 |
| **21:00** | 19.84 | 27.30 | 25.41 | 23.69 |
| **22:00** | 19.49 | 26.36 | 24.65 | 23.03 |
| **23:00** | 19.19 | 25.58 | 24.00 | 22.47 |

**Table 7** Fluid Average Temperature [$^{\circ}C$] Versus Orientation - Uniform Riser – Configuration One – August - Slope 45°

| **Hour** | **North** | **South** | **West** | **East** |
| --- | --- | --- | --- | --- |
| **00:00** | 33.59 | 36.31 | 36.86 | 34.79 |
| **01:00** | 33.16 | 35.72 | 36.24 | 34.29 |
| **02:00** | 32.76 | 35.16 | 35.66 | 33.82 |
| **03:00** | 32.48 | 34.69 | 35.15 | 33.62 |
| **04:00** | 32.50 | 34.46 | 34.76 | 34.11 |
| **05:00** | 32.82 | 34.68 | 34.50 | 35.30 |
| **06:00** | 33.36 | 35.39 | 34.34 | 36.96 |
| **07:00** | 34.07 | 36.52 | 34.33 | 38.84 |
| **08:00** | 34.84 | 37.95 | 34.71 | 40.50 |
| **09:00** | 35.70 | 39.63 | 35.55 | 41.97 |
| **10:00** | 36.55 | 41.31 | 36.79 | 43.05 |
| **11:00** | 37.34 | 42.81 | 38.30 | 43.63 |
| **12:00** | 38.04 | 44.05 | 39.98 | 43.73 |
| **13:00** | 38.62 | 44.88 | 41.69 | 43.52 |
| **14:00** | 39.02 | 45.21 | 43.21 | 43.12 |
| **15:00** | 39.10 | 44.96 | 44.12 | 42.55 |
| **16:00** | 38.85 | 44.31 | 44.26 | 41.81 |
| **17:00** | 38.31 | 43.36 | 43.75 | 40.90 |
| **18:00** | 37.56 | 42.15 | 42.78 | 39.82 |
| **19:00** | 36.73 | 40.89 | 41.61 | 38.72 |
| **20:00** | 36.03 | 39.78 | 40.47 | 37.74 |
| **21:00** | 35.30 | 38.73 | 39.41 | 36.84 |
| **22:00** | 34.64 | 37.79 | 38.43 | 36.03 |
| **23:00** | 34.09 | 37.00 | 37.60 | 35.36 |

## **A.5 Multiple Riser - Configuration Two – Fluid Average Temperature, Slope 45°- Reference Figure B3**

**Table 8** Fluid Average Temperature [$^{\circ}C$] Versus Orientation - Multiple Riser - Configuration Two – November - Slope 45°

| **Hour** | **North** | **South** | **West** | **East** |
| --- | --- | --- | --- | --- |
| **00:00** | 21.63 | 30.33 | 26.46 | 25.21 |
| **01:00** | 21.48 | 29.64 | 26.01 | 24.83 |
| **02:00** | 21.33 | 29.00 | 25.60 | 24.49 |
| **03:00** | 21.20 | 28.40 | 25.21 | 24.16 |
| **04:00** | 21.10 | 27.97 | 24.88 | 24.04 |
| **05:00** | 21.09 | 27.99 | 24.64 | 24.47 |
| **06:00** | 21.20 | 28.72 | 24.52 | 25.55 |
| **07:00** | 21.43 | 30.15 | 24.53 | 27.08 |
| **08:00** | 21.77 | 32.12 | 24.87 | 28.71 |
| **09:00** | 22.19 | 34.49 | 25.73 | 30.20 |
| **10:00** | 22.65 | 37.05 | 27.09 | 31.32 |
| **11:00** | 23.13 | 39.71 | 29.00 | 32.03 |
| **12:00** | 23.52 | 41.31 | 30.75 | 32.20 |
| **13:00** | 23.77 | 42.07 | 32.09 | 32.10 |
| **14:00** | 23.84 | 41.91 | 32.67 | 31.74 |
| **15:00** | 23.78 | 41.15 | 32.61 | 31.21 |
| **16:00** | 23.64 | 40.09 | 32.22 | 30.59 |
| **17:00** | 23.44 | 38.83 | 31.59 | 29.88 |
| **18:00** | 23.17 | 37.34 | 30.78 | 29.08 |
| **19:00** | 22.85 | 35.78 | 29.86 | 28.23 |
| **20:00** | 22.55 | 34.37 | 29.00 | 27.45 |
| **21:00** | 22.27 | 33.11 | 28.20 | 26.75 |
| **22:00** | 22.01 | 31.99 | 27.50 | 26.14 |
| **23:00** | 21.80 | 31.06 | 26.90 | 25.63 |

**Table 9** Fluid Average Temperature [$^{\circ}C$] Versus Orientation - Multiple Riser - Configuration Two – January - Slope 45°

| **Hour** | **North** | **South** | **West** | **East** |
| --- | --- | --- | --- | --- |
| **00:00** | 14.55 | 18.69 | 16.66 | 16.17 |
| **01:00** | 14.41 | 18.31 | 16.40 | 15.93 |
| **02:00** | 14.28 | 17.95 | 16.16 | 15.71 |
| **03:00** | 14.15 | 17.61 | 15.92 | 15.50 |
| **04:00** | 14.03 | 17.29 | 15.70 | 15.30 |
| **05:00** | 13.94 | 17.10 | 15.51 | 15.25 |
| **06:00** | 13.94 | 17.24 | 15.42 | 15.55 |
| **07:00** | 14.06 | 17.76 | 15.43 | 16.16 |
| **08:00** | 14.32 | 18.60 | 15.63 | 16.95 |
| **09:00** | 14.70 | 19.71 | 16.08 | 17.82 |
| **10:00** | 15.14 | 20.93 | 16.75 | 18.61 |
| **11:00** | 15.57 | 22.19 | 17.59 | 19.23 |
| **12:00** | 15.92 | 23.20 | 18.46 | 19.59 |
| **13:00** | 16.17 | 23.87 | 19.21 | 19.75 |
| **14:00** | 16.28 | 24.13 | 19.69 | 19.72 |
| **15:00** | 16.28 | 24.01 | 19.82 | 19.55 |
| **16:00** | 16.19 | 23.65 | 19.73 | 19.27 |
| **17:00** | 16.04 | 23.12 | 19.48 | 18.92 |
| **18:00** | 15.82 | 22.42 | 19.08 | 18.48 |
| **19:00** | 15.57 | 21.63 | 18.59 | 17.99 |
| **20:00** | 15.32 | 20.89 | 18.12 | 17.53 |
| **21:00** | 15.09 | 20.21 | 17.68 | 17.11 |
| **22:00** | 14.88 | 19.62 | 17.29 | 16.75 |
| **23:00** | 14.70 | 19.11 | 16.95 | 16.44 |

**Table 10** Fluid Average Temperature [$^{\circ}C$] Versus Orientation - Multiple Riser - Configuration Two – March - Slope 45°

| **Hour** | **North** | **South** | **West** | **East** |
| --- | --- | --- | --- | --- |
| **00:00** | 20.13 | 28.56 | 26.56 | 24.43 |
| **01:00** | 19.83 | 27.75 | 25.88 | 23.86 |
| **02:00** | 19.54 | 26.99 | 25.24 | 23.33 |
| **03:00** | 19.28 | 26.28 | 24.64 | 22.88 |
| **04:00** | 19.10 | 25.76 | 24.13 | 22.80 |
| **05:00** | 19.08 | 25.71 | 23.75 | 23.48 |
| **06:00** | 19.27 | 26.29 | 23.51 | 24.89 |
| **07:00** | 19.65 | 27.50 | 23.42 | 26.76 |
| **08:00** | 20.21 | 29.35 | 23.74 | 28.83 |
| **09:00** | 20.92 | 31.80 | 24.67 | 30.87 |
| **10:00** | 21.70 | 34.46 | 26.19 | 32.56 |
| **11:00** | 22.50 | 37.19 | 28.29 | 33.74 |
| **12:00** | 23.16 | 39.16 | 30.48 | 34.09 |
| **13:00** | 23.67 | 40.45 | 32.55 | 34.05 |
| **14:00** | 23.97 | 40.91 | 34.14 | 33.72 |
| **15:00** | 24.01 | 40.54 | 34.91 | 33.11 |
| **16:00** | 23.83 | 39.59 | 34.78 | 32.28 |
| **17:00** | 23.49 | 38.28 | 34.10 | 31.28 |
| **18:00** | 22.99 | 36.63 | 32.99 | 30.09 |
| **19:00** | 22.40 | 34.86 | 31.68 | 28.84 |
| **20:00** | 21.84 | 33.25 | 30.41 | 27.70 |
| **21:00** | 21.32 | 31.79 | 29.24 | 26.69 |
| **22:00** | 20.86 | 30.51 | 28.18 | 25.79 |
| **23:00** | 20.46 | 29.43 | 27.28 | 25.04 |

**Table 11** Fluid Average Temperature [$^{\circ}C$] Versus Orientation - Multiple Riser - Configuration Two – August - Slope 45°

| **Hour** | **North** | **South** | **West** | **East** |
| --- | --- | --- | --- | --- |
| **00:00** | 36.13 | 40.88 | 40.76 | 37.79 |
| **01:00** | 35.51 | 39.93 | 39.86 | 37.05 |
| **02:00** | 34.94 | 39.06 | 39.01 | 36.37 |
| **03:00** | 34.51 | 38.26 | 38.26 | 36.02 |
| **04:00** | 34.48 | 37.56 | 37.71 | 36.53 |
| **05:00** | 34.86 | 37.19 | 37.33 | 38.05 |
| **06:00** | 35.61 | 37.44 | 37.09 | 40.42 |
| **07:00** | 36.57 | 38.42 | 37.03 | 43.14 |
| **08:00** | 37.64 | 40.04 | 37.55 | 45.70 |
| **09:00** | 38.87 | 42.19 | 38.83 | 47.97 |
| **10:00** | 40.12 | 44.76 | 40.72 | 49.70 |
| **11:00** | 41.42 | 47.34 | 43.11 | 50.72 |
| **12:00** | 42.42 | 49.76 | 45.67 | 50.88 |
| **13:00** | 43.21 | 51.53 | 48.15 | 50.55 |
| **14:00** | 43.73 | 52.64 | 50.22 | 49.92 |
| **15:00** | 43.88 | 52.96 | 51.48 | 49.04 |
| **16:00** | 43.56 | 52.49 | 51.55 | 47.93 |
| **17:00** | 42.85 | 51.45 | 50.65 | 46.64 |
| **18:00** | 41.84 | 50.03 | 49.14 | 45.15 |
| **19:00** | 40.66 | 48.27 | 47.42 | 43.55 |
| **20:00** | 39.54 | 46.45 | 45.79 | 42.08 |
| **21:00** | 38.50 | 44.79 | 44.26 | 40.76 |
| **22:00** | 37.57 | 43.29 | 42.89 | 39.59 |
| **23:00** | 36.77 | 41.96 | 41.73 | 38.60 |

## **A.6 Multiple Riser - Configuration Three – Fluid Average Temperature, Slope 45°- Reference Figure B5**

**Table 12** Fluid Average Temperature [$^{\circ}C$] Versus Orientation - Multiple Riser - Configuration Three – November - Slope 45°

| **Hour** | **North** | **South** | **West** | **East** |
| --- | --- | --- | --- | --- |
| **00:00** | 21.63 | 30.33 | 26.43 | 25.21 |
| **01:00** | 21.47 | 29.65 | 25.99 | 24.84 |
| **02:00** | 21.33 | 29.02 | 25.59 | 24.50 |
| **03:00** | 21.20 | 28.43 | 25.20 | 24.18 |
| **04:00** | 21.10 | 27.99 | 24.87 | 24.05 |
| **05:00** | 21.08 | 28.02 | 24.63 | 24.48 |
| **06:00** | 21.19 | 28.75 | 24.51 | 25.56 |
| **07:00** | 21.42 | 30.20 | 24.51 | 27.10 |
| **08:00** | 21.76 | 32.20 | 24.86 | 28.74 |
| **09:00** | 22.18 | 34.60 | 25.72 | 30.22 |
| **10:00** | 22.64 | 37.17 | 27.10 | 31.34 |
| **11:00** | 23.10 | 39.56 | 28.87 | 31.97 |
| **12:00** | 23.49 | 41.19 | 30.63 | 32.16 |
| **13:00** | 23.75 | 41.98 | 32.00 | 32.07 |
| **14:00** | 23.83 | 41.86 | 32.63 | 31.72 |
| **15:00** | 23.77 | 41.12 | 32.59 | 31.20 |
| **16:00** | 23.63 | 40.07 | 32.22 | 30.58 |
| **17:00** | 23.43 | 38.80 | 31.59 | 29.87 |
| **18:00** | 23.16 | 37.30 | 30.76 | 29.07 |
| **19:00** | 22.85 | 35.75 | 29.85 | 28.21 |
| **20:00** | 22.55 | 34.36 | 28.99 | 27.44 |
| **21:00** | 22.26 | 33.11 | 28.20 | 26.75 |
| **22:00** | 22.01 | 32.01 | 27.50 | 26.14 |
| **23:00** | 21.80 | 31.09 | 26.91 | 25.63 |

**Table 13** Fluid Average Temperature [$^{\circ}C$] Versus Orientation - Multiple Riser - Configuration Three – January - Slope 45°

| **Hour** | **North** | **South** | **West** | **East** |
| --- | --- | --- | --- | --- |
| **00:00** | 14.54 | 18.68 | 16.65 | 16.16 |
| **01:00** | 14.40 | 18.30 | 16.39 | 15.93 |
| **02:00** | 14.27 | 17.95 | 16.15 | 15.71 |
| **03:00** | 14.14 | 17.61 | 15.92 | 15.50 |
| **04:00** | 14.02 | 17.30 | 15.70 | 15.31 |
| **05:00** | 13.93 | 17.10 | 15.51 | 15.26 |
| **06:00** | 13.93 | 17.24 | 15.41 | 15.56 |
| **07:00** | 14.06 | 17.74 | 15.42 | 16.17 |
| **08:00** | 14.32 | 18.56 | 15.61 | 16.95 |
| **09:00** | 14.70 | 19.67 | 16.06 | 17.81 |
| **10:00** | 15.14 | 20.87 | 16.73 | 18.59 |
| **11:00** | 15.57 | 22.12 | 17.56 | 19.19 |
| **12:00** | 15.92 | 23.13 | 18.43 | 19.54 |
| **13:00** | 16.16 | 23.79 | 19.17 | 19.70 |
| **14:00** | 16.27 | 24.04 | 19.65 | 19.67 |
| **15:00** | 16.26 | 23.90 | 19.77 | 19.49 |
| **16:00** | 16.17 | 23.53 | 19.68 | 19.21 |
| **17:00** | 16.01 | 22.99 | 19.42 | 18.86 |
| **18:00** | 15.80 | 22.30 | 19.02 | 18.42 |
| **19:00** | 15.54 | 21.52 | 18.54 | 17.94 |
| **20:00** | 15.29 | 20.80 | 18.08 | 17.49 |
| **21:00** | 15.06 | 20.14 | 17.64 | 17.08 |
| **22:00** | 14.86 | 19.55 | 17.26 | 16.72 |
| **23:00** | 14.68 | 19.06 | 16.93 | 16.42 |

**Table 14** Fluid Average Temperature [$^{\circ}C$] Versus Orientation - Multiple Riser - Configuration Three – March - Slope 45°

| **Hour** | **North** | **South** | **West** | **East** |
| --- | --- | --- | --- | --- |
| **00:00** | 20.13 | 28.55 | 26.47 | 24.44 |
| **01:00** | 19.83 | 27.75 | 25.80 | 23.88 |
| **02:00** | 19.55 | 27.00 | 25.17 | 23.35 |
| **03:00** | 19.29 | 26.30 | 24.59 | 22.90 |
| **04:00** | 19.10 | 25.78 | 24.08 | 22.81 |
| **05:00** | 19.09 | 25.75 | 23.72 | 23.50 |
| **06:00** | 19.27 | 26.34 | 23.49 | 24.93 |
| **07:00** | 19.64 | 27.55 | 23.40 | 26.80 |
| **08:00** | 20.20 | 29.43 | 23.75 | 28.88 |
| **09:00** | 20.91 | 31.91 | 24.75 | 30.93 |
| **10:00** | 21.69 | 34.58 | 26.34 | 32.59 |
| **11:00** | 22.46 | 37.15 | 28.38 | 33.67 |
| **12:00** | 23.12 | 39.11 | 30.59 | 34.03 |
| **13:00** | 23.63 | 40.40 | 32.66 | 34.00 |
| **14:00** | 23.93 | 40.85 | 34.23 | 33.68 |
| **15:00** | 23.97 | 40.47 | 34.97 | 33.08 |
| **16:00** | 23.79 | 39.52 | 34.75 | 32.25 |
| **17:00** | 23.44 | 38.18 | 33.99 | 31.24 |
| **18:00** | 22.94 | 36.52 | 32.83 | 30.05 |
| **19:00** | 22.36 | 34.77 | 31.50 | 28.81 |
| **20:00** | 21.81 | 33.18 | 30.26 | 27.69 |
| **21:00** | 21.30 | 31.75 | 29.11 | 26.68 |
| **22:00** | 20.84 | 30.49 | 28.08 | 25.80 |
| **23:00** | 20.46 | 29.43 | 27.21 | 25.06 |

**Table 15** Fluid Average Temperature [$^{\circ}C$] Versus Orientation - Multiple Riser - Configuration Three – August - Slope 45°

| **Hour** | **North** | **South** | **West** | **East** |
| --- | --- | --- | --- | --- |
| **00:00** | 36.08 | 39.96 | 40.72 | 37.74 |
| **01:00** | 35.48 | 39.11 | 39.83 | 37.03 |
| **02:00** | 34.91 | 38.31 | 38.99 | 36.36 |
| **03:00** | 34.50 | 37.62 | 38.25 | 36.01 |
| **04:00** | 34.49 | 37.25 | 37.70 | 36.63 |
| **05:00** | 34.91 | 37.49 | 37.34 | 38.34 |
| **06:00** | 35.68 | 38.45 | 37.10 | 40.85 |
| **07:00** | 36.63 | 40.04 | 37.03 | 43.58 |
| **08:00** | 37.71 | 42.16 | 37.53 | 46.10 |
| **09:00** | 38.94 | 44.72 | 38.82 | 48.33 |
| **10:00** | 40.19 | 47.28 | 40.70 | 49.93 |
| **11:00** | 41.37 | 49.61 | 43.04 | 50.73 |
| **12:00** | 42.37 | 51.41 | 45.62 | 50.80 |
| **13:00** | 43.16 | 52.56 | 48.12 | 50.40 |
| **14:00** | 43.68 | 52.92 | 50.20 | 49.72 |
| **15:00** | 43.82 | 52.49 | 51.47 | 48.81 |
| **16:00** | 43.50 | 51.47 | 51.54 | 47.69 |
| **17:00** | 42.75 | 50.05 | 50.63 | 46.35 |
| **18:00** | 41.71 | 48.31 | 49.11 | 44.82 |
| **19:00** | 40.54 | 46.49 | 47.38 | 43.25 |
| **20:00** | 39.44 | 44.84 | 45.75 | 41.85 |
| **21:00** | 38.42 | 43.34 | 44.23 | 40.58 |
| **22:00** | 37.51 | 42.01 | 42.87 | 39.46 |
| **23:00** | 36.73 | 40.90 | 41.71 | 38.52 |

## **A.7 Uniform Riser - Configuration One – Fluid Average Temperature, Slope 90°- Reference Figure B1**

**Table 16** Fluid Average Temperature [$^{\circ}C$] Versus Orientation - Uniform Riser – Configuration One – November - Slope 90°

| **Hour** | **North** | **South** | **West** | **East** |
| --- | --- | --- | --- | --- |
| **00:00** | 20.50 | 26.46 | 23.11 | 22.23 |
| **01:00** | 20.41 | 26.02 | 22.88 | 22.04 |
| **02:00** | 20.34 | 25.63 | 22.66 | 21.87 |
| **03:00** | 20.26 | 25.25 | 22.46 | 21.71 |
| **04:00** | 20.21 | 25.00 | 22.28 | 21.74 |
| **05:00** | 20.20 | 25.09 | 22.15 | 22.21 |
| **06:00** | 20.25 | 25.60 | 22.07 | 23.01 |
| **07:00** | 20.38 | 26.51 | 22.03 | 24.00 |
| **08:00** | 20.57 | 27.68 | 22.06 | 24.79 |
| **09:00** | 20.81 | 29.05 | 22.25 | 25.32 |
| **10:00** | 21.06 | 30.52 | 22.74 | 25.61 |
| **11:00** | 21.31 | 31.86 | 23.53 | 25.75 |
| **12:00** | 21.53 | 32.91 | 24.53 | 25.78 |
| **13:00** | 21.66 | 33.54 | 25.49 | 25.71 |
| **14:00** | 21.71 | 33.61 | 26.07 | 25.53 |
| **15:00** | 21.67 | 33.27 | 26.22 | 25.28 |
| **16:00** | 21.60 | 32.70 | 26.13 | 24.98 |
| **17:00** | 21.49 | 31.94 | 25.87 | 24.63 |
| **18:00** | 21.34 | 31.00 | 25.46 | 24.21 |
| **19:00** | 21.18 | 30.01 | 24.99 | 23.79 |
| **20:00** | 21.02 | 29.11 | 24.54 | 23.39 |
| **21:00** | 20.86 | 28.29 | 24.11 | 23.03 |
| **22:00** | 20.71 | 27.56 | 23.72 | 22.72 |
| **23:00** | 20.60 | 26.95 | 23.40 | 22.45 |

**Table 17** Fluid Average Temperature [$^{\circ}C$] Versus Orientation - Uniform Riser – Configuration One – January - Slope 90°

| **Hour** | **North** | **South** | **West** | **East** |
| --- | --- | --- | --- | --- |
| **00:00** | 13.37 | 16.43 | 14.69 | 14.27 |
| **01:00** | 13.30 | 16.19 | 14.55 | 14.15 |
| **02:00** | 13.24 | 15.96 | 14.42 | 14.04 |
| **03:00** | 13.17 | 15.75 | 14.29 | 13.93 |
| **04:00** | 13.12 | 15.54 | 14.16 | 13.83 |
| **05:00** | 13.12 | 15.45 | 14.06 | 13.86 |
| **06:00** | 13.17 | 15.59 | 13.99 | 14.15 |
| **07:00** | 13.28 | 15.97 | 13.98 | 14.60 |
| **08:00** | 13.45 | 16.52 | 14.04 | 15.06 |
| **09:00** | 13.65 | 17.21 | 14.20 | 15.46 |
| **10:00** | 13.85 | 17.94 | 14.48 | 15.75 |
| **11:00** | 14.03 | 18.66 | 14.88 | 15.95 |
| **12:00** | 14.16 | 19.28 | 15.37 | 16.08 |
| **13:00** | 14.23 | 19.73 | 15.87 | 16.12 |
| **14:00** | 14.24 | 19.91 | 16.21 | 16.08 |
| **15:00** | 14.21 | 19.84 | 16.33 | 15.98 |
| **16:00** | 14.16 | 19.62 | 16.31 | 15.84 |
| **17:00** | 14.08 | 19.28 | 16.19 | 15.66 |
| **18:00** | 13.97 | 18.82 | 15.99 | 15.43 |
| **19:00** | 13.85 | 18.32 | 15.74 | 15.19 |
| **20:00** | 13.73 | 17.84 | 15.48 | 14.95 |
| **21:00** | 13.61 | 17.40 | 15.24 | 14.74 |
| **22:00** | 13.51 | 17.02 | 15.03 | 14.56 |
| **23:00** | 13.43 | 16.70 | 14.85 | 14.40 |

**Table 18** Fluid Average Temperature [$^{\circ}C$] Versus Orientation - Uniform Riser – Configuration One – March - Slope 90°

| **Hour** | **North** | **South** | **West** | **East** |
| --- | --- | --- | --- | --- |
| **00:00** | 17.27 | 22.38 | 21.26 | 19.65 |
| **01:00** | 17.15 | 21.96 | 20.91 | 19.38 |
| **02:00** | 17.02 | 21.57 | 20.58 | 19.13 |
| **03:00** | 16.91 | 21.20 | 20.27 | 18.94 |
| **04:00** | 16.83 | 20.93 | 20.00 | 19.07 |
| **05:00** | 16.81 | 20.92 | 19.80 | 19.73 |
| **06:00** | 16.87 | 21.23 | 19.65 | 20.75 |
| **07:00** | 17.02 | 21.84 | 19.56 | 21.92 |
| **08:00** | 17.25 | 22.76 | 19.55 | 22.92 |
| **09:00** | 17.53 | 23.93 | 19.68 | 23.64 |
| **10:00** | 17.83 | 25.20 | 20.07 | 24.01 |
| **11:00** | 18.12 | 26.41 | 20.80 | 24.18 |
| **12:00** | 18.39 | 27.42 | 21.83 | 24.21 |
| **13:00** | 18.61 | 28.17 | 23.06 | 24.15 |
| **14:00** | 18.76 | 28.52 | 24.24 | 24.00 |
| **15:00** | 18.80 | 28.46 | 24.99 | 23.74 |
| **16:00** | 18.76 | 28.08 | 25.16 | 23.39 |
| **17:00** | 18.63 | 27.47 | 24.97 | 22.94 |
| **18:00** | 18.44 | 26.64 | 24.49 | 22.38 |
| **19:00** | 18.21 | 25.74 | 23.87 | 21.80 |
| **20:00** | 17.98 | 24.90 | 23.25 | 21.26 |
| **21:00** | 17.77 | 24.13 | 22.66 | 20.77 |
| **22:00** | 17.57 | 23.45 | 22.12 | 20.33 |
| **23:00** | 17.41 | 22.87 | 21.66 | 19.97 |

**Table 19** Fluid Average Temperature [$^{\circ}C$] Versus Orientation - Uniform Riser – Configuration One – August - Slope 90°

| **Hour** | **North** | **South** | **West** | **East** |
| --- | --- | --- | --- | --- |
| **00:00** | 29.37 | 32.00 | 34.25 | 31.75 |
| **01:00** | 29.17 | 31.62 | 33.72 | 31.38 |
| **02:00** | 28.97 | 31.26 | 33.22 | 31.04 |
| **03:00** | 28.91 | 30.96 | 32.79 | 31.15 |
| **04:00** | 29.03 | 30.81 | 32.47 | 32.15 |
| **05:00** | 29.18 | 30.93 | 32.27 | 33.81 |
| **06:00** | 29.35 | 31.41 | 32.14 | 35.82 |
| **07:00** | 29.55 | 32.21 | 32.02 | 37.53 |
| **08:00** | 29.79 | 33.25 | 31.97 | 38.54 |
| **09:00** | 30.06 | 34.44 | 32.12 | 38.98 |
| **10:00** | 30.34 | 35.58 | 32.67 | 38.97 |
| **11:00** | 30.60 | 36.55 | 33.68 | 38.76 |
| **12:00** | 30.82 | 37.27 | 35.11 | 38.44 |
| **13:00** | 31.00 | 37.67 | 37.05 | 38.08 |
| **14:00** | 31.17 | 37.66 | 38.75 | 37.68 |
| **15:00** | 31.35 | 37.37 | 39.93 | 37.20 |
| **16:00** | 31.37 | 36.91 | 40.18 | 36.64 |
| **17:00** | 31.23 | 36.29 | 39.74 | 35.98 |
| **18:00** | 30.97 | 35.54 | 38.93 | 35.23 |
| **19:00** | 30.67 | 34.78 | 37.98 | 34.47 |
| **20:00** | 30.40 | 34.12 | 37.13 | 33.80 |
| **21:00** | 30.09 | 33.47 | 36.25 | 33.17 |
| **22:00** | 29.82 | 32.89 | 35.46 | 32.61 |
| **23:00** | 29.57 | 32.40 | 34.78 | 32.14 |

## **A.8 Multiple Riser - Configuration Two – Fluid Average Temperature, Slope 90°- Reference Figure B3**

**Table 20** Fluid Average Temperature [$^{\circ}C$] Versus Orientation - Multiple Riser - Configuration Two – November - Slope 90°

| **Hour** | **North** | **South** | **West** | **East** |
| --- | --- | --- | --- | --- |
| **00:00** | 20.81 | 29.21 | 24.56 | 23.27 |
| **01:00** | 20.71 | 28.59 | 24.23 | 23.01 |
| **02:00** | 20.61 | 28.02 | 23.93 | 22.78 |
| **03:00** | 20.52 | 27.48 | 23.64 | 22.55 |
| **04:00** | 20.45 | 27.12 | 23.39 | 22.58 |
| **05:00** | 20.44 | 27.24 | 23.20 | 23.22 |
| **06:00** | 20.51 | 28.01 | 23.08 | 24.41 |
| **07:00** | 20.66 | 29.40 | 23.02 | 25.80 |
| **08:00** | 20.90 | 31.20 | 23.03 | 26.94 |
| **09:00** | 21.20 | 33.28 | 23.27 | 27.67 |
| **10:00** | 21.51 | 35.49 | 23.92 | 28.05 |
| **11:00** | 21.84 | 37.66 | 25.09 | 28.23 |
| **12:00** | 22.11 | 39.06 | 26.54 | 28.22 |
| **13:00** | 22.27 | 39.74 | 27.90 | 28.08 |
| **14:00** | 22.32 | 39.59 | 28.65 | 27.79 |
| **15:00** | 22.27 | 38.89 | 28.80 | 27.41 |
| **16:00** | 22.18 | 37.95 | 28.65 | 26.98 |
| **17:00** | 22.05 | 36.81 | 28.28 | 26.49 |
| **18:00** | 21.86 | 35.46 | 27.73 | 25.95 |
| **19:00** | 21.65 | 34.07 | 27.09 | 25.37 |
| **20:00** | 21.45 | 32.81 | 26.46 | 24.83 |
| **21:00** | 21.25 | 31.68 | 25.88 | 24.34 |
| **22:00** | 21.08 | 30.69 | 25.36 | 23.92 |
| **23:00** | 20.93 | 29.86 | 24.92 | 23.56 |

**Table 21** Fluid Average Temperature [$^{\circ}C$] Versus Orientation - Multiple Riser - Configuration Two – January - Slope 90°

| **Hour** | **North** | **South** | **West** | **East** |
| --- | --- | --- | --- | --- |
| **00:00** | 13.66 | 17.99 | 15.55 | 14.94 |
| **01:00** | 13.58 | 17.65 | 15.35 | 14.78 |
| **02:00** | 13.49 | 17.33 | 15.17 | 14.62 |
| **03:00** | 13.40 | 17.02 | 14.99 | 14.47 |
| **04:00** | 13.34 | 16.74 | 14.82 | 14.34 |
| **05:00** | 13.33 | 16.59 | 14.68 | 14.36 |
| **06:00** | 13.39 | 16.78 | 14.58 | 14.75 |
| **07:00** | 13.54 | 17.29 | 14.56 | 15.35 |
| **08:00** | 13.77 | 18.07 | 14.62 | 16.00 |
| **09:00** | 14.03 | 19.04 | 14.81 | 16.56 |
| **10:00** | 14.29 | 20.08 | 15.15 | 16.97 |
| **11:00** | 14.52 | 21.15 | 15.69 | 17.25 |
| **12:00** | 14.69 | 22.02 | 16.36 | 17.41 |
| **13:00** | 14.78 | 22.61 | 17.02 | 17.46 |
| **14:00** | 14.79 | 22.84 | 17.51 | 17.41 |
| **15:00** | 14.75 | 22.72 | 17.68 | 17.27 |
| **16:00** | 14.68 | 22.40 | 17.67 | 17.07 |
| **17:00** | 14.58 | 21.92 | 17.53 | 16.82 |
| **18:00** | 14.44 | 21.30 | 17.26 | 16.52 |
| **19:00** | 14.29 | 20.60 | 16.93 | 16.19 |
| **20:00** | 14.13 | 19.94 | 16.60 | 15.87 |
| **21:00** | 13.98 | 19.34 | 16.28 | 15.59 |
| **22:00** | 13.86 | 18.82 | 15.99 | 15.34 |
| **23:00** | 13.75 | 18.37 | 15.75 | 15.12 |

**Table 22** Fluid Average Temperature [$^{\circ}C$] Versus Orientation - Multiple Riser - Configuration Two – March - Slope 90°

| **Hour** | **North** | **South** | **West** | **East** |
| --- | --- | --- | --- | --- |
| **00:00** | 17.75 | 24.95 | 23.42 | 20.76 |
| **01:00** | 17.59 | 24.36 | 22.93 | 20.41 |
| **02:00** | 17.44 | 23.80 | 22.47 | 20.15 |
| **03:00** | 17.29 | 23.28 | 22.03 | 20.30 |
| **04:00** | 17.19 | 22.91 | 21.66 | 21.22 |
| **05:00** | 17.17 | 22.89 | 21.38 | 22.73 |
| **06:00** | 17.24 | 23.32 | 21.18 | 24.41 |
| **07:00** | 17.43 | 24.20 | 21.03 | 25.87 |
| **08:00** | 17.71 | 25.53 | 20.98 | 26.89 |
| **09:00** | 18.05 | 27.25 | 21.12 | 27.38 |
| **10:00** | 18.43 | 29.09 | 21.63 | 27.57 |
| **11:00** | 18.81 | 31.02 | 22.66 | 27.54 |
| **12:00** | 19.14 | 32.41 | 24.15 | 27.40 |
| **13:00** | 19.42 | 33.33 | 25.89 | 27.13 |
| **14:00** | 19.60 | 33.67 | 27.53 | 26.72 |
| **15:00** | 19.66 | 33.44 | 28.58 | 26.19 |
| **16:00** | 19.60 | 32.79 | 28.77 | 25.55 |
| **17:00** | 19.44 | 31.87 | 28.47 | 24.81 |
| **18:00** | 19.20 | 30.71 | 27.79 | 24.01 |
| **19:00** | 18.92 | 29.46 | 26.94 | 23.27 |
| **20:00** | 18.64 | 28.31 | 26.09 | 22.61 |
| **21:00** | 18.37 | 27.27 | 25.28 | 22.02 |
| **22:00** | 18.13 | 26.35 | 24.55 | 21.53 |
| **23:00** | 17.93 | 25.57 | 23.92 | 22.02 |

**Table 23** Fluid Average Temperature [$^{\circ}C$] Versus Orientation - Multiple Riser - Configuration Two – August - Slope 90°

| **Hour** | **North** | **South** | **West** | **East** |
| --- | --- | --- | --- | --- |
| **00:00** | 30.07 | 33.94 | 36.99 | 33.66 |
| **01:00** | 29.85 | 33.47 | 36.34 | 33.20 |
| **02:00** | 29.63 | 33.02 | 35.72 | 32.77 |
| **03:00** | 29.52 | 32.63 | 35.17 | 32.74 |
| **04:00** | 29.63 | 32.39 | 34.75 | 33.81 |
| **05:00** | 29.81 | 32.41 | 34.45 | 35.79 |
| **06:00** | 30.00 | 32.81 | 34.22 | 38.18 |
| **07:00** | 30.20 | 33.61 | 33.99 | 40.29 |
| **08:00** | 30.44 | 34.76 | 33.81 | 41.73 |
| **09:00** | 30.73 | 36.18 | 33.87 | 42.47 |
| **10:00** | 31.04 | 37.63 | 34.37 | 42.58 |
| **11:00** | 31.35 | 39.02 | 35.47 | 42.37 |
| **12:00** | 31.61 | 40.00 | 37.16 | 42.01 |
| **13:00** | 31.82 | 40.58 | 39.26 | 41.59 |
| **14:00** | 32.01 | 40.70 | 41.44 | 41.10 |
| **15:00** | 32.22 | 40.45 | 43.20 | 40.51 |
| **16:00** | 32.30 | 39.94 | 43.86 | 39.82 |
| **17:00** | 32.19 | 39.24 | 43.56 | 39.02 |
| **18:00** | 31.92 | 38.36 | 42.68 | 38.09 |
| **19:00** | 31.59 | 37.43 | 41.57 | 37.14 |
| **20:00** | 31.24 | 36.56 | 40.48 | 36.26 |
| **21:00** | 30.91 | 35.76 | 39.43 | 35.46 |
| **22:00** | 30.59 | 35.05 | 38.47 | 34.76 |
| **23:00** | 30.32 | 34.44 | 37.65 | 34.17 |

## **A.9 Multiple Riser - Configuration Three – Fluid Average Temperature, Slope 90°- Reference Figure B5**

**Table 24** Fluid Average Temperature [$^{\circ}C$] Versus Orientation - Multiple Riser - Configuration Three – November - Slope 90°

| **Hour** | **North** | **South** | **West** | **East** |
| --- | --- | --- | --- | --- |
| **00:00** | 20.82 | 29.26 | 24.57 | 23.29 |
| **01:00** | 20.71 | 28.64 | 24.25 | 23.04 |
| **02:00** | 20.62 | 28.07 | 23.95 | 22.80 |
| **03:00** | 20.53 | 27.54 | 23.66 | 22.58 |
| **04:00** | 20.45 | 27.16 | 23.41 | 22.57 |
| **05:00** | 20.43 | 27.22 | 23.21 | 23.13 |
| **06:00** | 20.49 | 27.90 | 23.09 | 24.25 |
| **07:00** | 20.63 | 29.17 | 23.00 | 25.59 |
| **08:00** | 20.87 | 30.89 | 23.01 | 26.74 |
| **09:00** | 21.16 | 32.93 | 23.21 | 27.52 |
| **10:00** | 21.47 | 35.12 | 23.82 | 27.93 |
| **11:00** | 21.79 | 37.20 | 24.90 | 28.11 |
| **12:00** | 22.06 | 38.70 | 26.31 | 28.14 |
| **13:00** | 22.23 | 39.50 | 27.67 | 28.02 |
| **14:00** | 22.29 | 39.49 | 28.49 | 27.76 |
| **15:00** | 22.26 | 38.88 | 28.71 | 27.40 |
| **16:00** | 22.18 | 37.97 | 28.59 | 26.98 |
| **17:00** | 22.04 | 36.84 | 28.24 | 26.50 |
| **18:00** | 21.86 | 35.51 | 27.70 | 25.95 |
| **19:00** | 21.66 | 34.12 | 27.06 | 25.37 |
| **20:00** | 21.45 | 32.87 | 26.45 | 24.84 |
| **21:00** | 21.26 | 31.75 | 25.88 | 24.36 |
| **22:00** | 21.08 | 30.76 | 25.37 | 23.94 |
| **23:00** | 20.94 | 29.94 | 24.94 | 23.59 |

**Table 25** Fluid Average Temperature [$^{\circ}C$] Versus Orientation - Multiple Riser - Configuration Three – January - Slope 90°

| **Hour** | **North** | **South** | **West** | **East** |
| --- | --- | --- | --- | --- |
| **00:00** | 13.66 | 18.01 | 15.54 | 14.95 |
| **01:00** | 13.58 | 17.67 | 15.35 | 14.79 |
| **02:00** | 13.49 | 17.36 | 15.17 | 14.63 |
| **03:00** | 13.41 | 17.06 | 15.00 | 14.48 |
| **04:00** | 13.34 | 16.77 | 14.83 | 14.35 |
| **05:00** | 13.32 | 16.60 | 14.68 | 14.35 |
| **06:00** | 13.38 | 16.74 | 14.58 | 14.69 |
| **07:00** | 13.52 | 17.19 | 14.55 | 15.25 |
| **08:00** | 13.74 | 17.91 | 14.60 | 15.88 |
| **09:00** | 14.00 | 18.85 | 14.78 | 16.45 |
| **10:00** | 14.25 | 19.87 | 15.11 | 16.87 |
| **11:00** | 14.49 | 20.93 | 15.61 | 17.17 |
| **12:00** | 14.66 | 21.82 | 16.25 | 17.34 |
| **13:00** | 14.76 | 22.43 | 16.91 | 17.40 |
| **14:00** | 14.77 | 22.71 | 17.41 | 17.36 |
| **15:00** | 14.74 | 22.62 | 17.62 | 17.23 |
| **16:00** | 14.67 | 22.32 | 17.63 | 17.04 |
| **17:00** | 14.57 | 21.85 | 17.50 | 16.80 |
| **18:00** | 14.43 | 21.24 | 17.24 | 16.50 |
| **19:00** | 14.28 | 20.56 | 16.91 | 16.18 |
| **20:00** | 14.12 | 19.92 | 16.58 | 15.86 |
| **21:00** | 13.98 | 19.33 | 16.27 | 15.58 |
| **22:00** | 13.85 | 18.82 | 15.99 | 15.33 |
| **23:00** | 13.75 | 18.38 | 15.76 | 15.13 |

**Table 26** Fluid Average Temperature [$^{\circ}C$] Versus Orientation - Multiple Riser - Configuration Three – March - Slope 90°

| **Hour** | **North** | **South** | **West** | **East** |
| --- | --- | --- | --- | --- |
| **00:00** | 16.55 | 23.88 | 22.23 | 19.99 |
| **01:00** | 16.44 | 23.34 | 21.80 | 19.67 |
| **02:00** | 16.33 | 22.83 | 21.38 | 19.37 |
| **03:00** | 16.22 | 22.35 | 21.00 | 19.13 |
| **04:00** | 16.15 | 21.99 | 20.65 | 19.27 |
| **05:00** | 16.15 | 21.95 | 20.39 | 20.13 |
| **06:00** | 16.22 | 22.31 | 20.18 | 21.57 |
| **07:00** | 16.34 | 23.02 | 19.95 | 23.12 |
| **08:00** | 16.51 | 24.16 | 19.78 | 24.48 |
| **09:00** | 16.77 | 25.74 | 19.82 | 25.46 |
| **10:00** | 17.08 | 27.51 | 20.25 | 25.95 |
| **11:00** | 17.41 | 29.35 | 21.17 | 26.13 |
| **12:00** | 17.73 | 30.77 | 22.57 | 26.12 |
| **13:00** | 18.00 | 31.76 | 24.28 | 26.00 |
| **14:00** | 18.20 | 32.21 | 25.94 | 25.77 |
| **15:00** | 18.28 | 32.08 | 27.07 | 25.39 |
| **16:00** | 18.26 | 31.52 | 27.37 | 24.90 |
| **17:00** | 18.13 | 30.65 | 27.12 | 24.29 |
| **18:00** | 17.90 | 29.51 | 26.48 | 23.55 |
| **19:00** | 17.61 | 28.27 | 25.63 | 22.75 |
| **20:00** | 17.33 | 27.14 | 24.81 | 22.03 |
| **21:00** | 17.08 | 26.11 | 24.03 | 21.38 |
| **22:00** | 16.86 | 25.21 | 23.32 | 20.82 |
| **23:00** | 16.69 | 24.48 | 22.74 | 20.36 |

**Table 27** Fluid Average Temperature [$^{\circ}C$] Versus Orientation - Multiple Riser - Configuration Three – August - Slope 90°

| **Hour** | **North** | **South** | **West** | **East** |
| --- | --- | --- | --- | --- |
| **00:00** | 30.10 | 34.00 | 37.01 | 33.74 |
| **01:00** | 29.88 | 33.53 | 36.37 | 33.29 |
| **02:00** | 29.66 | 33.08 | 35.76 | 32.85 |
| **03:00** | 29.53 | 32.69 | 35.21 | 32.74 |
| **04:00** | 29.61 | 32.43 | 34.78 | 33.62 |
| **05:00** | 29.77 | 32.40 | 34.48 | 35.43 |
| **06:00** | 29.96 | 32.75 | 34.23 | 37.73 |
| **07:00** | 30.15 | 33.46 | 33.98 | 39.82 |
| **08:00** | 30.38 | 34.52 | 33.79 | 41.33 |
| **09:00** | 30.66 | 35.87 | 33.82 | 42.20 |
| **10:00** | 30.97 | 37.30 | 34.27 | 42.42 |
| **11:00** | 31.27 | 38.66 | 35.28 | 42.28 |
| **12:00** | 31.53 | 39.71 | 36.88 | 41.97 |
| **13:00** | 31.75 | 40.37 | 38.90 | 41.58 |
| **14:00** | 31.94 | 40.58 | 41.05 | 41.11 |
| **15:00** | 32.15 | 40.40 | 42.85 | 40.56 |
| **16:00** | 32.25 | 39.95 | 43.63 | 39.89 |
| **17:00** | 32.16 | 39.28 | 43.43 | 39.09 |
| **18:00** | 31.92 | 38.42 | 42.63 | 38.17 |
| **19:00** | 31.60 | 37.49 | 41.56 | 37.21 |
| **20:00** | 31.26 | 36.63 | 40.50 | 36.34 |
| **21:00** | 30.93 | 35.83 | 39.47 | 35.54 |
| **22:00** | 30.61 | 35.11 | 38.53 | 34.84 |
| **23:00** | 30.34 | 34.51 | 37.71 | 34.25 |

## **A.10 Hourly Heat Flux [W/m^2^] Through building masonry without BIST integration - Slope 90°**

**Table 28** Hourly Heat Flux [W/m^2^] Through building masonry without BIST integration – November – Slope 90°

| **Hour** | **North** | **South** | **West** | **East** |
| --- | --- | --- | --- | --- |
| **00:00** | -0.83 | -3.71 | -1.83 | -1.98 |
| **01:00** | -0.86 | -3.74 | -1.87 | -2.00 |
| **02:00** | -0.88 | -3.74 | -1.89 | -2.01 |
| **03:00** | -0.81 | -3.64 | -1.81 | -1.92 |
| **04:00** | -0.71 | -3.51 | -1.71 | -1.81 |
| **05:00** | -0.63 | -3.41 | -1.63 | -1.72 |
| **06:00** | 0.13 | 0.66 | -0.86 | 4.70 |
| **07:00** | 1.00 | 5.30 | 0.03 | 8.25 |
| **08:00** | 1.56 | 9.46 | 0.60 | 8.88 |
| **09:00** | 1.84 | 11.47 | 0.89 | 6.45 |
| **10:00** | 2.16 | 10.90 | 1.21 | 2.93 |
| **11:00** | 2.11 | 12.20 | 4.50 | 0.89 |
| **12:00** | 1.73 | 10.01 | 7.06 | 0.64 |
| **13:00** | 1.18 | 7.28 | 8.33 | 0.06 |
| **14:00** | 0.49 | 3.62 | 7.50 | -0.68 |
| **15:00** | -0.14 | -1.36 | 2.28 | -1.35 |
| **16:00** | -0.49 | -3.40 | -1.65 | -1.70 |
| **17:00** | -0.58 | -3.40 | -1.60 | -1.81 |
| **18:00** | -0.69 | -3.61 | -1.69 | -1.94 |
| **19:00** | -0.81 | -3.80 | -1.83 | -2.07 |
| **20:00** | -0.94 | -3.97 | -1.98 | -2.19 |
| **21:00** | -0.96 | -4.01 | -2.01 | -2.22 |
| **22:00** | -0.97 | -4.04 | -2.04 | -2.22 |
| **23:00** | -0.85 | -3.94 | -1.93 | -2.09 |

**Table 29** Hourly Heat Flux [W/m^2^] Through building masonry without BIST integration – January –Slope 90°

| **Hour** | **North** | **South** | **West** | **East** |
| --- | --- | --- | --- | --- |
| **00:00** | -1.40 | -2.62 | -1.83 | -1.85 |
| **01:00** | -1.34 | -2.55 | -1.76 | -1.79 |
| **02:00** | -1.38 | -2.62 | -1.83 | -1.83 |
| **03:00** | -1.45 | -2.69 | -1.90 | -1.89 |
| **04:00** | -1.50 | -2.72 | -1.94 | -1.94 |
| **05:00** | -1.55 | -2.75 | -1.98 | -1.98 |
| **06:00** | -1.51 | -2.70 | -1.94 | -1.93 |
| **07:00** | -1.45 | -2.52 | -1.88 | -1.64 |
| **08:00** | -0.92 | 0.81 | -1.34 | 2.37 |
| **09:00** | -0.18 | 3.46 | -0.60 | 3.69 |
| **10:00** | 0.49 | 4.55 | 0.08 | 3.07 |
| **11:00** | 0.96 | 5.21 | 0.55 | 2.12 |
| **12:00** | 1.08 | 4.69 | 1.61 | 0.57 |
| **13:00** | 0.83 | 4.41 | 2.57 | 0.37 |
| **14:00** | 0.72 | 4.19 | 3.76 | 0.28 |
| **15:00** | 0.28 | 2.04 | 3.12 | -0.18 |
| **16:00** | -0.33 | 0.23 | 2.10 | -0.80 |
| **17:00** | -0.77 | -2.08 | -1.30 | -1.24 |
| **18:00** | -0.82 | -2.09 | -1.28 | -1.30 |
| **19:00** | -0.88 | -2.14 | -1.31 | -1.37 |
| **20:00** | -0.95 | -2.23 | -1.39 | -1.44 |
| **21:00** | -1.03 | -2.33 | -1.47 | -1.52 |
| **22:00** | -1.11 | -2.42 | -1.56 | -1.60 |
| **23:00** | -1.19 | -2.51 | -1.65 | -1.68 |

**Table 30** Hourly Heat Flux [W/m^2^] Through building masonry without BIST integration – March –Slope 90°

| **Hour** | **North** | **South** | **West** | **East** |
| --- | --- | --- | --- | --- |
| **00:00** | -1.39 | -4.16 | -1.78 | -3.18 |
| **01:00** | -1.38 | -4.17 | -1.79 | -3.16 |
| **02:00** | -1.55 | -4.33 | -1.95 | -3.32 |
| **03:00** | -1.64 | -4.41 | -2.04 | -3.39 |
| **04:00** | -1.41 | -4.16 | -1.81 | -3.14 |
| **05:00** | -1.19 | -3.93 | -1.60 | -2.91 |
| **06:00** | -0.98 | -3.59 | -1.38 | -1.12 |
| **07:00** | -0.50 | -0.59 | -0.90 | 6.71 |
| **08:00** | 0.45 | 2.95 | 0.06 | 9.82 |
| **09:00** | 1.35 | 5.80 | 0.96 | 9.35 |
| **10:00** | 1.78 | 8.49 | 1.40 | 7.84 |
| **11:00** | 2.05 | 9.66 | 1.67 | 4.41 |
| **12:00** | 2.15 | 9.36 | 2.73 | 0.41 |
| **13:00** | 1.57 | 8.33 | 3.36 | 0.22 |
| **14:00** | 1.15 | 6.34 | 4.23 | -0.03 |
| **15:00** | 0.68 | 4.02 | 3.56 | -0.47 |
| **16:00** | -0.07 | 0.74 | 2.39 | -1.24 |
| **17:00** | -0.65 | -2.16 | -1.15 | -2.17 |
| **18:00** | -0.95 | -3.82 | -1.38 | -2.88 |
| **19:00** | -1.05 | -3.89 | -1.45 | -2.99 |
| **20:00** | -1.15 | -4.05 | -1.56 | -3.10 |
| **21:00** | -1.26 | -4.22 | -1.67 | -3.21 |
| **22:00** | -1.27 | -4.25 | -1.68 | -3.21 |
| **23:00** | -1.26 | -4.28 | -1.69 | -3.21 |

**Table 31** Hourly Heat Flux [W/m^2^] Through building masonry without BIST integration – August –Slope 90°

| **Hour** | **North** | **South** | **West** | **East** |
| --- | --- | --- | --- | --- |
| **00:00** | -0.65 | -2.15 | -2.58 | -1.85 |
| **01:00** | -0.70 | -2.20 | -2.66 | -1.79 |
| **02:00** | -0.82 | -2.31 | -2.80 | -1.83 |
| **03:00** | -0.95 | -2.43 | -2.94 | -1.89 |
| **04:00** | -1.07 | -2.54 | -3.06 | -1.94 |
| **05:00** | 1.66 | -1.41 | -1.95 | -1.98 |
| **06:00** | 1.60 | -0.06 | -1.15 | -1.93 |
| **07:00** | 1.42 | 3.06 | -0.49 | -1.64 |
| **08:00** | 1.80 | 5.64 | -0.12 | 2.37 |
| **09:00** | 2.17 | 7.65 | 0.26 | 3.69 |
| **10:00** | 2.57 | 8.74 | 0.69 | 3.07 |
| **11:00** | 2.77 | 8.39 | 3.66 | 2.12 |
| **12:00** | 2.67 | 7.32 | 7.00 | 0.57 |
| **13:00** | 2.39 | 6.22 | 10.45 | 0.37 |
| **14:00** | 2.13 | 3.93 | 11.90 | 0.28 |
| **15:00** | 1.79 | 1.33 | 11.91 | -0.18 |
| **16:00** | 2.77 | -0.06 | 10.48 | -0.80 |
| **17:00** | 1.76 | -0.88 | 2.70 | -1.24 |
| **18:00** | 0.01 | -1.39 | -2.08 | -1.30 |
| **19:00** | -0.16 | -1.70 | -2.15 | -1.37 |
| **20:00** | -0.39 | -1.98 | -2.38 | -1.44 |
| **21:00** | -0.66 | -2.24 | -2.67 | -1.52 |
| **22:00** | -0.90 | -2.50 | -2.94 | -1.60 |
| **23:00** | -0.97 | -2.58 | -3.05 | -1.68 |

## **A.11 Uniform Riser - Configuration One – Hourly Heat Flux [W/m^2^] Through Building Masonry with BIST - Slope 90°**

**Table 32** Hourly Heat Flux [W/m^2^] Through building masonry with BIST – Uniform Riser - Configuration One – November – All Orientations - Slope 90°

| **Hour** | **North** | **South** | **West** | **East** |
| --- | --- | --- | --- | --- |
| **00:00** | 0.48 | 4.95 | 1.77 | 1.65 |
| **01:00** | 0.46 | 4.68 | 1.69 | 1.57 |
| **02:00** | 0.44 | 4.42 | 1.61 | 1.49 |
| **03:00** | 0.42 | 4.17 | 1.53 | 1.41 |
| **04:00** | 0.40 | 3.94 | 1.45 | 1.34 |
| **05:00** | 0.38 | 3.78 | 1.37 | 1.34 |
| **06:00** | 0.37 | 3.84 | 1.30 | 1.51 |
| **07:00** | 0.37 | 4.19 | 1.25 | 1.81 |
| **08:00** | 0.37 | 4.82 | 1.21 | 2.13 |
| **09:00** | 0.39 | 5.56 | 1.18 | 2.40 |
| **10:00** | 0.41 | 6.32 | 1.21 | 2.56 |
| **11:00** | 0.44 | 7.08 | 1.33 | 2.60 |
| **12:00** | 0.48 | 7.71 | 1.58 | 2.59 |
| **13:00** | 0.52 | 8.14 | 1.93 | 2.57 |
| **14:00** | 0.56 | 8.27 | 2.24 | 2.52 |
| **15:00** | 0.57 | 8.09 | 2.42 | 2.45 |
| **16:00** | 0.57 | 7.75 | 2.45 | 2.35 |
| **17:00** | 0.56 | 7.36 | 2.41 | 2.25 |
| **18:00** | 0.55 | 6.97 | 2.34 | 2.15 |
| **19:00** | 0.54 | 6.59 | 2.24 | 2.07 |
| **20:00** | 0.55 | 6.23 | 2.15 | 2.00 |
| **21:00** | 0.54 | 5.89 | 2.05 | 1.92 |
| **22:00** | 0.53 | 5.56 | 1.95 | 1.83 |
| **23:00** | 0.51 | 5.24 | 1.85 | 1.74 |

**Table 33** Hourly Heat Flux [W/m^2^] Through building masonry with BIST – Uniform Riser - Configuration One – January – All Orientations - Slope 90°

| **Hour** | **North** | **South** | **West** | **East** |
| --- | --- | --- | --- | --- |
| **00:00** | 0.58 | 2.86 | 1.45 | 1.27 |
| **01:00** | 0.55 | 2.71 | 1.38 | 1.21 |
| **02:00** | 0.53 | 2.57 | 1.32 | 1.15 |
| **03:00** | 0.51 | 2.45 | 1.26 | 1.11 |
| **04:00** | 0.49 | 2.33 | 1.21 | 1.06 |
| **05:00** | 0.48 | 2.22 | 1.16 | 1.02 |
| **06:00** | 0.46 | 2.11 | 1.10 | 0.97 |
| **07:00** | 0.46 | 2.06 | 1.06 | 1.01 |
| **08:00** | 0.48 | 2.17 | 1.04 | 1.19 |
| **09:00** | 0.51 | 2.40 | 1.04 | 1.45 |
| **10:00** | 0.57 | 2.71 | 1.06 | 1.68 |
| **11:00** | 0.63 | 3.06 | 1.10 | 1.84 |
| **12:00** | 0.69 | 3.40 | 1.19 | 1.93 |
| **13:00** | 0.73 | 3.72 | 1.35 | 1.96 |
| **14:00** | 0.77 | 3.96 | 1.55 | 1.94 |
| **15:00** | 0.79 | 4.11 | 1.76 | 1.90 |
| **16:00** | 0.78 | 4.14 | 1.89 | 1.84 |
| **17:00** | 0.76 | 4.05 | 1.93 | 1.77 |
| **18:00** | 0.73 | 3.90 | 1.90 | 1.69 |
| **19:00** | 0.70 | 3.72 | 1.84 | 1.61 |
| **20:00** | 0.68 | 3.54 | 1.76 | 1.54 |
| **21:00** | 0.65 | 3.36 | 1.68 | 1.46 |
| **22:00** | 0.63 | 3.18 | 1.60 | 1.40 |
| **23:00** | 0.60 | 3.02 | 1.53 | 1.33 |

**Table 34** Hourly Heat Flux [W/m^2^] Through building masonry with BIST – Uniform Riser - Configuration One – March – All Orientations - Slope 90°

| **Hour** | **North** | **South** | **West** | **East** |
| --- | --- | --- | --- | --- |
| **00:00** | 0.93 | 4.71 | 3.32 | 2.82 |
| **01:00** | 0.90 | 4.47 | 3.16 | 2.68 |
| **02:00** | 0.88 | 4.24 | 3.02 | 2.55 |
| **03:00** | 0.85 | 4.02 | 2.87 | 2.41 |
| **04:00** | 0.81 | 3.80 | 2.72 | 2.27 |
| **05:00** | 0.76 | 3.59 | 2.57 | 2.17 |
| **06:00** | 0.72 | 3.42 | 2.42 | 2.33 |
| **07:00** | 0.69 | 3.38 | 2.30 | 2.83 |
| **08:00** | 0.67 | 3.50 | 2.21 | 3.41 |
| **09:00** | 0.69 | 3.80 | 2.13 | 3.98 |
| **10:00** | 0.75 | 4.26 | 2.09 | 4.43 |
| **11:00** | 0.81 | 4.83 | 2.10 | 4.68 |
| **12:00** | 0.89 | 5.40 | 2.23 | 4.68 |
| **13:00** | 0.97 | 5.93 | 2.56 | 4.59 |
| **14:00** | 1.05 | 6.36 | 3.06 | 4.47 |
| **15:00** | 1.12 | 6.62 | 3.64 | 4.35 |
| **16:00** | 1.17 | 6.87 | 4.18 | 4.20 |
| **17:00** | 1.18 | 6.78 | 4.52 | 4.03 |
| **18:00** | 1.16 | 6.51 | 4.49 | 3.84 |
| **19:00** | 1.13 | 6.20 | 4.33 | 3.66 |
| **20:00** | 1.09 | 5.88 | 4.12 | 3.48 |
| **21:00** | 1.06 | 5.57 | 3.91 | 3.30 |
| **22:00** | 1.02 | 5.27 | 3.69 | 3.14 |
| **23:00** | 0.98 | 4.98 | 3.49 | 2.97 |

**Table 35** Hourly Heat Flux [W/m^2^] Through building masonry with BIST – Uniform Riser - Configuration One – August – All Orientations - Slope 90°

| **Hour** | **North** | **South** | **West** | **East** |
| --- | --- | --- | --- | --- |
| **00:00** | 1.15 | 3.28 | 4.10 | 3.08 |
| **01:00** | 1.12 | 3.13 | 3.88 | 2.93 |
| **02:00** | 1.08 | 2.98 | 3.68 | 2.79 |
| **03:00** | 1.04 | 2.84 | 3.50 | 2.87 |
| **04:00** | 1.06 | 2.70 | 3.33 | 3.32 |
| **05:00** | 1.11 | 2.58 | 3.18 | 4.04 |
| **06:00** | 1.15 | 2.53 | 3.04 | 4.82 |
| **07:00** | 1.15 | 2.65 | 2.92 | 5.50 |
| **08:00** | 1.17 | 2.94 | 2.83 | 5.96 |
| **09:00** | 1.19 | 3.39 | 2.75 | 6.08 |
| **10:00** | 1.22 | 3.89 | 2.74 | 5.96 |
| **11:00** | 1.27 | 4.36 | 2.90 | 5.78 |
| **12:00** | 1.32 | 4.76 | 3.27 | 5.56 |
| **13:00** | 1.36 | 5.04 | 3.82 | 5.34 |
| **14:00** | 1.38 | 5.14 | 4.47 | 5.12 |
| **15:00** | 1.41 | 5.07 | 5.11 | 4.88 |
| **16:00** | 1.46 | 4.90 | 5.57 | 4.63 |
| **17:00** | 1.47 | 4.68 | 5.69 | 4.39 |
| **18:00** | 1.43 | 4.45 | 5.56 | 4.17 |
| **19:00** | 1.38 | 4.23 | 5.34 | 3.96 |
| **20:00** | 1.33 | 4.02 | 5.08 | 3.76 |
| **21:00** | 1.29 | 3.82 | 4.82 | 3.58 |
| **22:00** | 1.25 | 3.63 | 4.57 | 3.41 |
| **23:00** | 1.20 | 3.45 | 4.33 | 3.24 |

## **A.12 Multiple Riser - Configuration Two – Hourly Heat Flux [W/m^2^] Through Building Masonry with BIST - Slope 90°**

**Table 36** Hourly Heat Flux [W/m^2^] Through building masonry with BIST – Multiple Riser - Configuration Two – November – All Orientations - Slope 90°

| **Hour** | **North** | **South** | **West** | **East** |
| --- | --- | --- | --- | --- |
| **00:00** | 0.83 | 7.81 | 3.09 | 2.93 |
| **01:00** | 0.80 | 7.39 | 2.95 | 2.78 |
| **02:00** | 0.77 | 6.99 | 2.80 | 2.64 |
| **03:00** | 0.73 | 6.60 | 2.66 | 2.49 |
| **04:00** | 0.68 | 6.22 | 2.52 | 2.34 |
| **05:00** | 0.62 | 5.93 | 2.36 | 2.34 |
| **06:00** | 0.56 | 5.89 | 2.22 | 2.63 |
| **07:00** | 0.52 | 6.19 | 2.11 | 3.25 |
| **08:00** | 0.50 | 6.84 | 2.03 | 3.92 |
| **09:00** | 0.51 | 7.68 | 1.97 | 4.41 |
| **10:00** | 0.54 | 8.58 | 2.01 | 4.59 |
| **11:00** | 0.60 | 9.83 | 2.27 | 4.62 |
| **12:00** | 0.70 | 10.89 | 2.76 | 4.60 |
| **13:00** | 0.82 | 11.69 | 3.56 | 4.57 |
| **14:00** | 0.91 | 12.04 | 4.21 | 4.48 |
| **15:00** | 0.94 | 11.93 | 4.36 | 4.35 |
| **16:00** | 0.95 | 11.55 | 4.25 | 4.19 |
| **17:00** | 0.95 | 11.10 | 4.10 | 4.03 |
| **18:00** | 0.95 | 10.63 | 3.96 | 3.87 |
| **19:00** | 0.95 | 10.14 | 3.82 | 3.72 |
| **20:00** | 0.94 | 9.66 | 3.68 | 3.56 |
| **21:00** | 0.93 | 9.18 | 3.54 | 3.41 |
| **22:00** | 0.90 | 8.71 | 3.39 | 3.25 |
| **23:00** | 0.86 | 8.24 | 3.24 | 3.08 |

**Table 37** Hourly Heat Flux [W/m^2^] Through building masonry with BIST – Multiple Riser - Configuration Two – January – All Orientations - Slope 90°

| **Hour** | **North** | **South** | **West** | **East** |
| --- | --- | --- | --- | --- |
| **00:00** | 0.79 | 4.32 | 2.09 | 1.76 |
| **01:00** | 0.76 | 4.11 | 2.00 | 1.68 |
| **02:00** | 0.74 | 3.91 | 1.93 | 1.61 |
| **03:00** | 0.72 | 3.73 | 1.85 | 1.54 |
| **04:00** | 0.71 | 3.56 | 1.78 | 1.49 |
| **05:00** | 0.68 | 3.38 | 1.72 | 1.43 |
| **06:00** | 0.65 | 3.21 | 1.64 | 1.37 |
| **07:00** | 0.63 | 3.12 | 1.58 | 1.41 |
| **08:00** | 0.63 | 3.22 | 1.53 | 1.61 |
| **09:00** | 0.65 | 3.49 | 1.50 | 1.90 |
| **10:00** | 0.69 | 3.87 | 1.49 | 2.21 |
| **11:00** | 0.74 | 4.30 | 1.52 | 2.41 |
| **12:00** | 0.79 | 4.72 | 1.62 | 2.48 |
| **13:00** | 0.83 | 5.26 | 1.80 | 2.48 |
| **14:00** | 0.86 | 5.70 | 2.05 | 2.47 |
| **15:00** | 0.89 | 5.96 | 2.33 | 2.44 |
| **16:00** | 0.91 | 6.03 | 2.52 | 2.38 |
| **17:00** | 0.91 | 5.89 | 2.57 | 2.31 |
| **18:00** | 0.90 | 5.70 | 2.55 | 2.23 |
| **19:00** | 0.89 | 5.47 | 2.49 | 2.15 |
| **20:00** | 0.87 | 5.24 | 2.42 | 2.08 |
| **21:00** | 0.86 | 5.01 | 2.34 | 2.00 |
| **22:00** | 0.85 | 4.78 | 2.27 | 1.92 |
| **23:00** | 0.82 | 4.55 | 2.19 | 1.84 |

**Table 38** Hourly Heat Flux [W/m^2^] Through building masonry with BIST – Multiple Riser - Configuration Two – March – All Orientations - Slope 90°

| **Hour** | **North** | **South** | **West** | **East** |
| --- | --- | --- | --- | --- |
| **00:00** | 1.31 | 7.90 | 5.78 | 4.53 |
| **01:00** | 1.28 | 7.51 | 5.53 | 4.31 |
| **02:00** | 1.25 | 7.14 | 5.29 | 4.11 |
| **03:00** | 1.21 | 6.76 | 5.04 | 3.91 |
| **04:00** | 1.15 | 6.39 | 4.78 | 3.70 |
| **05:00** | 1.08 | 6.03 | 4.52 | 3.53 |
| **06:00** | 1.01 | 5.73 | 4.27 | 3.59 |
| **07:00** | 0.96 | 5.61 | 4.04 | 4.05 |
| **08:00** | 0.92 | 5.75 | 3.84 | 4.73 |
| **09:00** | 0.91 | 6.19 | 3.70 | 5.43 |
| **10:00** | 0.94 | 6.87 | 3.57 | 5.99 |
| **11:00** | 0.99 | 7.66 | 3.51 | 6.39 |
| **12:00** | 1.06 | 8.64 | 3.66 | 6.48 |
| **13:00** | 1.14 | 9.53 | 4.09 | 6.48 |
| **14:00** | 1.24 | 10.23 | 4.78 | 6.44 |
| **15:00** | 1.35 | 10.69 | 5.60 | 6.36 |
| **16:00** | 1.43 | 10.86 | 6.35 | 6.25 |
| **17:00** | 1.48 | 10.75 | 7.03 | 6.09 |
| **18:00** | 1.49 | 10.46 | 7.16 | 5.92 |
| **19:00** | 1.48 | 10.06 | 7.07 | 5.70 |
| **20:00** | 1.46 | 9.63 | 6.88 | 5.47 |
| **21:00** | 1.43 | 9.20 | 6.63 | 5.24 |
| **22:00** | 1.40 | 8.76 | 6.35 | 5.00 |
| **23:00** | 1.36 | 8.32 | 6.06 | 4.77 |

**Table 39** Hourly Heat Flux [W/m^2^] Through building masonry with BIST – Multiple Riser - Configuration Two – August – All Orientations - Slope 90°

| **Hour** | **North** | **South** | **West** | **East** |
| --- | --- | --- | --- | --- |
| **00:00** | 1.65 | 4.95 | 6.86 | 4.85 |
| **01:00** | 1.60 | 4.73 | 6.54 | 4.62 |
| **02:00** | 1.56 | 4.52 | 6.24 | 4.40 |
| **03:00** | 1.52 | 4.32 | 5.96 | 4.44 |
| **04:00** | 1.50 | 4.10 | 5.67 | 4.99 |
| **05:00** | 1.52 | 3.91 | 5.39 | 5.92 |
| **06:00** | 1.53 | 3.83 | 5.13 | 6.93 |
| **07:00** | 1.52 | 3.94 | 4.91 | 7.95 |
| **08:00** | 1.51 | 4.26 | 4.72 | 8.61 |
| **09:00** | 1.52 | 4.78 | 4.54 | 8.83 |
| **10:00** | 1.55 | 5.37 | 4.46 | 8.75 |
| **11:00** | 1.61 | 6.01 | 4.62 | 8.55 |
| **12:00** | 1.67 | 6.55 | 5.17 | 8.30 |
| **13:00** | 1.71 | 6.96 | 6.01 | 8.02 |
| **14:00** | 1.74 | 7.15 | 6.99 | 7.73 |
| **15:00** | 1.78 | 7.10 | 8.04 | 7.41 |
| **16:00** | 1.85 | 6.93 | 8.78 | 7.07 |
| **17:00** | 1.89 | 6.69 | 8.99 | 6.75 |
| **18:00** | 1.88 | 6.43 | 8.84 | 6.45 |
| **19:00** | 1.84 | 6.17 | 8.55 | 6.16 |
| **20:00** | 1.81 | 5.91 | 8.22 | 5.88 |
| **21:00** | 1.78 | 5.66 | 7.88 | 5.61 |
| **22:00** | 1.74 | 5.42 | 7.53 | 5.35 |
| **23:00** | 1.70 | 5.18 | 7.19 | 5.10 |

## **A.13 Multiple Riser - Configuration Three – Hourly Heat Flux [W/m^2^] Through Building Masonry with BIST - Slope 90°**

**Table 40** Hourly Heat Flux [W/m^2^] Through building masonry with BIST – Multiple Riser - Configuration Three – November – All Orientations - Slope 90°

| **Hour** | **North** | **South** | **West** | **East** |
| --- | --- | --- | --- | --- |
| **00:00** | 0.83 | 7.93 | 3.39 | 2.87 |
| **01:00** | 0.80 | 7.51 | 3.24 | 2.73 |
| **02:00** | 0.76 | 7.10 | 3.08 | 2.59 |
| **03:00** | 0.72 | 6.70 | 2.93 | 2.44 |
| **04:00** | 0.68 | 6.32 | 2.77 | 2.30 |
| **05:00** | 0.62 | 6.03 | 2.60 | 2.30 |
| **06:00** | 0.57 | 5.99 | 2.44 | 2.61 |
| **07:00** | 0.53 | 6.31 | 2.31 | 3.16 |
| **08:00** | 0.53 | 7.12 | 2.21 | 3.74 |
| **09:00** | 0.55 | 8.08 | 2.15 | 4.21 |
| **10:00** | 0.58 | 9.10 | 2.17 | 4.39 |
| **11:00** | 0.63 | 10.22 | 2.42 | 4.43 |
| **12:00** | 0.73 | 11.21 | 2.92 | 4.43 |
| **13:00** | 0.84 | 11.96 | 3.59 | 4.41 |
| **14:00** | 0.92 | 12.28 | 4.17 | 4.34 |
| **15:00** | 0.95 | 12.14 | 4.51 | 4.21 |
| **16:00** | 0.96 | 11.76 | 4.55 | 4.07 |
| **17:00** | 0.96 | 11.31 | 4.47 | 3.92 |
| **18:00** | 0.96 | 10.82 | 4.34 | 3.77 |
| **19:00** | 0.96 | 10.33 | 4.20 | 3.63 |
| **20:00** | 0.94 | 9.83 | 4.05 | 3.48 |
| **21:00** | 0.93 | 9.34 | 3.88 | 3.33 |
| **22:00** | 0.89 | 8.85 | 3.71 | 3.18 |
| **23:00** | 0.86 | 8.38 | 3.55 | 3.02 |

**Table 41** Hourly Heat Flux [W/m^2^] Through building masonry with BIST – Multiple Riser - Configuration Three – January – All Orientations - Slope 90°

| **Hour** | **North** | **South** | **West** | **East** |
| --- | --- | --- | --- | --- |
| **00:00** | 0.80 | 4.23 | 2.04 | 1.85 |
| **01:00** | 0.77 | 4.03 | 1.95 | 1.76 |
| **02:00** | 0.75 | 3.83 | 1.88 | 1.69 |
| **03:00** | 0.73 | 3.65 | 1.80 | 1.62 |
| **04:00** | 0.71 | 3.48 | 1.73 | 1.56 |
| **05:00** | 0.69 | 3.31 | 1.66 | 1.49 |
| **06:00** | 0.67 | 3.15 | 1.59 | 1.42 |
| **07:00** | 0.65 | 3.06 | 1.53 | 1.48 |
| **08:00** | 0.65 | 3.16 | 1.49 | 1.73 |
| **09:00** | 0.67 | 3.44 | 1.47 | 2.01 |
| **10:00** | 0.70 | 3.84 | 1.46 | 2.27 |
| **11:00** | 0.74 | 4.27 | 1.49 | 2.48 |
| **12:00** | 0.80 | 4.70 | 1.58 | 2.56 |
| **13:00** | 0.84 | 5.09 | 1.76 | 2.58 |
| **14:00** | 0.88 | 5.51 | 2.00 | 2.57 |
| **15:00** | 0.92 | 5.77 | 2.26 | 2.55 |
| **16:00** | 0.93 | 5.84 | 2.42 | 2.49 |
| **17:00** | 0.93 | 5.73 | 2.47 | 2.42 |
| **18:00** | 0.92 | 5.54 | 2.45 | 2.34 |
| **19:00** | 0.90 | 5.33 | 2.40 | 2.26 |
| **20:00** | 0.88 | 5.10 | 2.34 | 2.18 |
| **21:00** | 0.86 | 4.88 | 2.27 | 2.10 |
| **22:00** | 0.85 | 4.67 | 2.20 | 2.02 |
| **23:00** | 0.83 | 4.45 | 2.13 | 1.94 |

**Table 42** Hourly Heat Flux [W/m^2^] Through building masonry with BIST – Multiple Riser - Configuration Three – March – All Orientations - Slope 90°

| **Hour** | **North** | **South** | **West** | **East** |
| --- | --- | --- | --- | --- |
| **00:00** | 1.31 | 7.62 | 5.54 | 3.91 |
| **01:00** | 1.28 | 7.25 | 5.29 | 3.73 |
| **02:00** | 1.25 | 6.89 | 5.06 | 3.57 |
| **03:00** | 1.20 | 6.53 | 4.82 | 3.39 |
| **04:00** | 1.14 | 6.17 | 4.57 | 3.20 |
| **05:00** | 1.07 | 5.82 | 4.32 | 3.04 |
| **06:00** | 0.99 | 5.52 | 4.07 | 3.07 |
| **07:00** | 0.92 | 5.40 | 3.84 | 3.44 |
| **08:00** | 0.87 | 5.52 | 3.64 | 4.00 |
| **09:00** | 0.87 | 5.98 | 3.50 | 4.58 |
| **10:00** | 0.89 | 6.69 | 3.39 | 5.06 |
| **11:00** | 0.93 | 7.50 | 3.35 | 5.37 |
| **12:00** | 0.99 | 8.44 | 3.52 | 5.45 |
| **13:00** | 1.07 | 9.27 | 3.97 | 5.46 |
| **14:00** | 1.16 | 9.93 | 4.68 | 5.43 |
| **15:00** | 1.25 | 10.37 | 5.48 | 5.38 |
| **16:00** | 1.33 | 10.52 | 6.38 | 5.30 |
| **17:00** | 1.38 | 10.38 | 6.87 | 5.19 |
| **18:00** | 1.41 | 10.08 | 6.92 | 5.04 |
| **19:00** | 1.43 | 9.69 | 6.78 | 4.87 |
| **20:00** | 1.42 | 9.29 | 6.58 | 4.68 |
| **21:00** | 1.41 | 8.87 | 6.33 | 4.50 |
| **22:00** | 1.38 | 8.44 | 6.07 | 4.30 |
| **23:00** | 1.35 | 8.02 | 5.80 | 4.11 |

**Table 43** Hourly Heat Flux [W/m^2^] Through building masonry with BIST – Multiple Riser - Configuration Three – August – All Orientations - Slope 90°

| **Hour** | **North** | **South** | **West** | **East** |
| --- | --- | --- | --- | --- |
| **00:00** | 1.73 | 4.78 | 6.66 | 4.81 |
| **01:00** | 1.69 | 4.56 | 6.35 | 4.58 |
| **02:00** | 1.64 | 4.36 | 6.06 | 4.36 |
| **03:00** | 1.60 | 4.17 | 5.78 | 4.38 |
| **04:00** | 1.58 | 3.97 | 5.50 | 4.85 |
| **05:00** | 1.60 | 3.78 | 5.22 | 5.67 |
| **06:00** | 1.60 | 3.68 | 4.97 | 6.76 |
| **07:00** | 1.59 | 3.76 | 4.75 | 7.70 |
| **08:00** | 1.58 | 4.03 | 4.55 | 8.36 |
| **09:00** | 1.58 | 4.46 | 4.38 | 8.59 |
| **10:00** | 1.62 | 4.98 | 4.30 | 8.53 |
| **11:00** | 1.68 | 5.51 | 4.44 | 8.35 |
| **12:00** | 1.73 | 6.09 | 4.88 | 8.12 |
| **13:00** | 1.77 | 6.52 | 5.59 | 7.86 |
| **14:00** | 1.80 | 6.74 | 6.60 | 7.58 |
| **15:00** | 1.84 | 6.73 | 7.62 | 7.27 |
| **16:00** | 1.91 | 6.59 | 8.39 | 6.96 |
| **17:00** | 1.96 | 6.39 | 8.63 | 6.65 |
| **18:00** | 1.95 | 6.16 | 8.51 | 6.36 |
| **19:00** | 1.92 | 5.92 | 8.24 | 6.08 |
| **20:00** | 1.89 | 5.69 | 7.94 | 5.81 |
| **21:00** | 1.87 | 5.46 | 7.63 | 5.55 |
| **22:00** | 1.83 | 5.23 | 7.30 | 5.30 |
| **23:00** | 1.79 | 5.00 | 6.97 | 5.06 |

## **A.14 Solar Radiation [W/m^2^] - Slope 0°**

**Table 44** Hourly Solar Radiation [W/m^2^] Versus All Months - Slope 0°

| **Hour** | **November** | **January** | **March** | **August** |
| --- | --- | --- | --- | --- |
| **00:00** | 0.00 | 0.00 | 0.00 | 0.00 |
| **01:00** | 0.00 | 0.00 | 0.00 | 0.00 |
| **02:00** | 0.00 | 0.00 | 0.00 | 0.00 |
| **03:00** | 0.00 | 0.00 | 0.00 | 0.00 |
| **04:00** | 0.00 | 0.00 | 0.00 | 0.00 |
| **05:00** | 0.00 | 0.00 | 0.00 | 0.00 |
| **06:00** | 0.00 | 0.00 | 12.18 | 139.31 |
| **07:00** | 80.58 | 1.14 | 157.41 | 349.05 |
| **08:00** | 244.36 | 94.71 | 346.19 | 554.44 |
| **09:00** | 404.56 | 203.48 | 500.28 | 728.59 |
| **10:00** | 510.53 | 271.97 | 662.06 | 854.80 |
| **11:00** | 519.57 | 305.45 | 728.90 | 909.26 |
| **12:00** | 541.30 | 293.65 | 722.65 | 862.71 |
| **13:00** | 465.41 | 254.55 | 666.52 | 786.30 |
| **14:00** | 353.84 | 216.71 | 568.33 | 729.44 |
| **15:00** | 206.04 | 134.65 | 429.40 | 570.36 |
| **16:00** | 43.82 | 51.12 | 248.68 | 386.56 |
| **17:00** | 0.00 | 0.00 | 85.47 | 218.75 |
| **18:00** | 0.00 | 0.00 | 0.00 | 39.06 |
| **19:00** | 0.00 | 0.00 | 0.00 | 0.00 |
| **20:00** | 0.00 | 0.00 | 0.00 | 0.00 |
| **21:00** | 0.00 | 0.00 | 0.00 | 0.00 |
| **22:00** | 0.00 | 0.00 | 0.00 | 0.00 |
| **23:00** | 0.00 | 0.00 | 0.00 | 0.00 |

## **A.15 Solar Radiation [W/m^2^] - Slope 45°**

**Table 45** Hourly Solar Radiation [W/m^2^] Versus Orientation - November - Slope 45°

| **Hour** | **North** | **South** | **West** | **East** |
| --- | --- | --- | --- | --- |
| **00:00** | 0.00 | 0.00 | 0.00 | 0.00 |
| **01:00** | 0.00 | 0.00 | 0.00 | 0.00 |
| **02:00** | 0.00 | 0.00 | 0.00 | 0.00 |
| **03:00** | 0.00 | 0.00 | 0.00 | 0.00 |
| **04:00** | 0.00 | 0.00 | 0.00 | 0.00 |
| **05:00** | 0.00 | 0.00 | 0.00 | 0.00 |
| **06:00** | 0.00 | 0.00 | 0.00 | 0.00 |
| **07:00** | 33.97 | 175.08 | 33.97 | 263.03 |
| **08:00** | 79.73 | 431.03 | 79.73 | 482.02 |
| **09:00** | 98.42 | 679.00 | 98.42 | 602.46 |
| **10:00** | 114.10 | 821.91 | 212.98 | 581.76 |
| **11:00** | 132.27 | 802.63 | 362.15 | 452.57 |
| **12:00** | 117.78 | 860.92 | 507.05 | 333.88 |
| **13:00** | 118.13 | 745.64 | 563.55 | 166.58 |
| **14:00** | 103.18 | 589.01 | 554.54 | 103.18 |
| **15:00** | 73.58 | 375.89 | 447.49 | 73.58 |
| **16:00** | 23.80 | 100.74 | 165.57 | 23.80 |
| **17:00** | 0.00 | 0.00 | 0.00 | 0.00 |
| **18:00** | 0.00 | 0.00 | 0.00 | 0.00 |
| **19:00** | 0.00 | 0.00 | 0.00 | 0.00 |
| **20:00** | 0.00 | 0.00 | 0.00 | 0.00 |
| **21:00** | 0.00 | 0.00 | 0.00 | 0.00 |
| **22:00** | 0.00 | 0.00 | 0.00 | 0.00 |
| **23:00** | 0.00 | 0.00 | 0.00 | 0.00 |

**Table 46** Hourly Solar Radiation [W/m^2^] Versus Orientation - January - Slope 45°

| **Hour** | **North** | **South** | **West** | **East** |
| --- | --- | --- | --- | --- |
| **00:00** | 0.00 | 0.00 | 0.00 | 0.00 |
| **01:00** | 0.00 | 0.00 | 0.00 | 0.00 |
| **02:00** | 0.00 | 0.00 | 0.00 | 0.00 |
| **03:00** | 0.00 | 0.00 | 0.00 | 0.00 |
| **04:00** | 0.00 | 0.00 | 0.00 | 0.00 |
| **05:00** | 0.00 | 0.00 | 0.00 | 0.00 |
| **06:00** | 0.00 | 0.00 | 0.00 | 0.00 |
| **07:00** | 0.00 | 4.53 | 0.39 | 8.89 |
| **08:00** | 43.50 | 172.84 | 43.50 | 204.51 |
| **09:00** | 85.25 | 325.21 | 85.25 | 308.98 |
| **10:00** | 121.07 | 396.59 | 130.02 | 316.93 |
| **11:00** | 139.20 | 430.07 | 211.03 | 291.83 |
| **12:00** | 142.00 | 399.53 | 254.31 | 232.53 |
| **13:00** | 110.03 | 367.00 | 267.54 | 149.44 |
| **14:00** | 89.91 | 334.58 | 287.45 | 89.91 |
| **15:00** | 65.21 | 213.59 | 222.23 | 65.21 |
| **16:00** | 24.85 | 106.87 | 145.35 | 24.85 |
| **17:00** | 0.00 | 0.00 | 0.00 | 0.00 |
| **18:00** | 0.00 | 0.00 | 0.00 | 0.00 |
| **19:00** | 0.00 | 0.00 | 0.00 | 0.00 |
| **20:00** | 0.00 | 0.00 | 0.00 | 0.00 |
| **21:00** | 0.00 | 0.00 | 0.00 | 0.00 |
| **22:00** | 0.00 | 0.00 | 0.00 | 0.00 |
| **23:00** | 0.00 | 0.00 | 0.00 | 0.00 |

**Table 47** Hourly Solar Radiation [W/m^2^] Versus Orientation - March - Slope 45°

| **Hour** | **North** | **South** | **West** | **East** |
| --- | --- | --- | --- | --- |
| **00:00** | 0.00 | 0.00 | 0.00 | 0.00 |
| **01:00** | 0.00 | 0.00 | 0.00 | 0.00 |
| **02:00** | 0.00 | 0.00 | 0.00 | 0.00 |
| **03:00** | 0.00 | 0.00 | 0.00 | 0.00 |
| **04:00** | 0.00 | 0.00 | 0.00 | 0.00 |
| **05:00** | 0.00 | 0.00 | 0.00 | 0.00 |
| **06:00** | 10.80 | 10.37 | 5.84 | 63.01 |
| **07:00** | 75.30 | 187.29 | 55.63 | 415.14 |
| **08:00** | 135.58 | 418.99 | 95.08 | 636.71 |
| **09:00** | 187.44 | 604.55 | 128.49 | 705.84 |
| **10:00** | 229.29 | 805.99 | 274.21 | 756.82 |
| **11:00** | 233.06 | 896.13 | 450.71 | 676.46 |
| **12:00** | 248.26 | 875.73 | 595.13 | 529.39 |
| **13:00** | 215.79 | 815.81 | 684.75 | 349.74 |
| **14:00** | 197.88 | 685.97 | 714.25 | 174.26 |
| **15:00** | 158.83 | 517.39 | 668.79 | 126.73 |
| **16:00** | 108.17 | 295.06 | 504.77 | 89.70 |
| **17:00** | 43.39 | 101.17 | 306.72 | 35.29 |
| **18:00** | 0.00 | 0.00 | 0.00 | 0.00 |
| **19:00** | 0.00 | 0.00 | 0.00 | 0.00 |
| **20:00** | 0.00 | 0.00 | 0.00 | 0.00 |
| **21:00** | 0.00 | 0.00 | 0.00 | 0.00 |
| **22:00** | 0.00 | 0.00 | 0.00 | 0.00 |
| **23:00** | 0.00 | 0.00 | 0.00 | 0.00 |

**Table 48** Hourly Solar Radiation [W/m^2^] Versus Orientation - August - Slope 45°

| **Hour** | **North** | **South** | **West** | **East** |
| --- | --- | --- | --- | --- |
| **00:00** | 0.00 | 0.00 | 0.00 | 0.00 |
| **01:00** | 0.00 | 0.00 | 0.00 | 0.00 |
| **02:00** | 0.00 | 0.00 | 0.00 | 0.00 |
| **03:00** | 0.00 | 0.00 | 0.00 | 0.00 |
| **04:00** | 0.00 | 0.00 | 0.00 | 0.00 |
| **05:00** | 0.00 | 0.00 | 0.00 | 0.00 |
| **06:00** | 162.81 | 69.86 | 56.04 | 410.69 |
| **07:00** | 283.77 | 274.02 | 90.69 | 730.54 |
| **08:00** | 362.81 | 503.66 | 111.05 | 897.36 |
| **09:00** | 418.18 | 707.30 | 164.33 | 954.23 |
| **10:00** | 455.50 | 857.33 | 387.18 | 920.97 |
| **11:00** | 472.89 | 924.00 | 589.82 | 805.16 |
| **12:00** | 458.22 | 876.27 | 714.48 | 620.79 |
| **13:00** | 427.91 | 788.96 | 785.31 | 434.60 |
| **14:00** | 406.67 | 716.90 | 881.29 | 247.79 |
| **15:00** | 347.75 | 532.69 | 835.86 | 127.41 |
| **16:00** | 273.64 | 325.28 | 723.27 | 96.58 |
| **17:00** | 207.35 | 136.22 | 568.18 | 71.37 |
| **18:00** | 73.45 | 19.58 | 193.46 | 19.58 |
| **19:00** | 0.00 | 0.00 | 0.00 | 0.00 |
| **20:00** | 0.00 | 0.00 | 0.00 | 0.00 |
| **21:00** | 0.00 | 0.00 | 0.00 | 0.00 |
| **22:00** | 0.00 | 0.00 | 0.00 | 0.00 |
| **23:00** | 0.00 | 0.00 | 0.00 | 0.00 |

## **A.16 Solar Radiation [W/m^2^] - Slope 90°**

**Table 49** Hourly Solar Radiation [W/m^2^] Versus Orientation - November - Slope 90°

| **Hour** | **North** | **South** | **West** | **East** |
| --- | --- | --- | --- | --- |
| **00:00** | 0.00 | 0.00 | 0.00 | 0.00 |
| **01:00** | 0.00 | 0.00 | 0.00 | 0.00 |
| **02:00** | 0.00 | 0.00 | 0.00 | 0.00 |
| **03:00** | 0.00 | 0.00 | 0.00 | 0.00 |
| **04:00** | 0.00 | 0.00 | 0.00 | 0.00 |
| **05:00** | 0.00 | 0.00 | 0.00 | 0.00 |
| **06:00** | 19.24 | 182.47 | 19.24 | 306.85 |
| **07:00** | 45.16 | 402.51 | 45.16 | 474.61 |
| **08:00** | 55.75 | 604.27 | 55.75 | 496.03 |
| **09:00** | 64.63 | 709.51 | 64.63 | 369.89 |
| **10:00** | 74.92 | 680.61 | 74.92 | 185.55 |
| **11:00** | 66.71 | 736.36 | 235.92 | 66.71 |
| **12:00** | 66.91 | 646.70 | 389.18 | 66.91 |
| **13:00** | 58.44 | 528.00 | 479.24 | 58.44 |
| **14:00** | 41.68 | 359.22 | 460.47 | 41.68 |
| **15:00** | 13.48 | 109.22 | 200.90 | 13.48 |
| **16:00** | 0.00 | 0.00 | 0.00 | 0.00 |
| **17:00** | 0.00 | 0.00 | 0.00 | 0.00 |
| **18:00** | 0.00 | 0.00 | 0.00 | 0.00 |
| **19:00** | 0.00 | 0.00 | 0.00 | 0.00 |
| **20:00** | 0.00 | 0.00 | 0.00 | 0.00 |
| **21:00** | 0.00 | 0.00 | 0.00 | 0.00 |
| **22:00** | 0.00 | 0.00 | 0.00 | 0.00 |
| **23:00** | 0.00 | 0.00 | 0.00 | 0.00 |

**Table 50** Hourly Solar Radiation [W/m^2^] Versus Orientation - January - Slope 90°

| **Hour** | **North** | **South** | **West** | **East** |
| --- | --- | --- | --- | --- |
| **00:00** | 0.00 | 0.00 | 0.00 | 0.00 |
| **01:00** | 0.00 | 0.00 | 0.00 | 0.00 |
| **02:00** | 0.00 | 0.00 | 0.00 | 0.00 |
| **03:00** | 0.00 | 0.00 | 0.00 | 0.00 |
| **04:00** | 0.00 | 0.00 | 0.00 | 0.00 |
| **05:00** | 0.00 | 0.00 | 0.00 | 0.00 |
| **06:00** | 0.00 | 0.00 | 0.00 | 0.00 |
| **07:00** | 0.22 | 5.47 | 0.22 | 11.63 |
| **08:00** | 24.64 | 169.11 | 24.64 | 213.89 |
| **09:00** | 48.29 | 294.51 | 48.29 | 271.56 |
| **10:00** | 68.58 | 342.25 | 68.58 | 229.59 |
| **11:00** | 78.84 | 363.64 | 78.84 | 168.14 |
| **12:00** | 80.43 | 333.35 | 127.98 | 80.43 |
| **13:00** | 62.33 | 313.48 | 172.83 | 62.33 |
| **14:00** | 50.93 | 296.32 | 229.66 | 50.93 |
| **15:00** | 36.94 | 196.24 | 208.47 | 36.94 |
| **16:00** | 14.08 | 111.23 | 165.65 | 14.08 |
| **17:00** | 0.00 | 0.00 | 0.00 | 0.00 |
| **18:00** | 0.00 | 0.00 | 0.00 | 0.00 |
| **19:00** | 0.00 | 0.00 | 0.00 | 0.00 |
| **20:00** | 0.00 | 0.00 | 0.00 | 0.00 |
| **21:00** | 0.00 | 0.00 | 0.00 | 0.00 |
| **22:00** | 0.00 | 0.00 | 0.00 | 0.00 |
| **23:00** | 0.00 | 0.00 | 0.00 | 0.00 |

**Table 51** Hourly Solar Radiation [W/m^2^] Versus Orientation - March - Slope 90°

| **Hour** | **North** | **South** | **West** | **East** |
| --- | --- | --- | --- | --- |
| **00:00** | 0.00 | 0.00 | 0.00 | 0.00 |
| **01:00** | 0.00 | 0.00 | 0.00 | 0.00 |
| **02:00** | 0.00 | 0.00 | 0.00 | 0.00 |
| **03:00** | 0.00 | 0.00 | 0.00 | 0.00 |
| **04:00** | 0.00 | 0.00 | 0.00 | 0.00 |
| **05:00** | 0.00 | 0.00 | 0.00 | 0.00 |
| **06:00** | 5.71 | 4.77 | 3.31 | 79.57 |
| **07:00** | 31.51 | 132.82 | 31.51 | 455.04 |
| **08:00** | 53.85 | 291.41 | 53.85 | 599.31 |
| **09:00** | 74.32 | 417.08 | 74.32 | 560.33 |
| **10:00** | 86.83 | 554.64 | 86.83 | 485.10 |
| **11:00** | 83.45 | 615.37 | 83.45 | 304.70 |
| **12:00** | 92.26 | 599.39 | 202.56 | 92.26 |
| **13:00** | 81.67 | 560.68 | 375.33 | 81.67 |
| **14:00** | 78.90 | 470.63 | 510.64 | 78.90 |
| **15:00** | 71.78 | 361.72 | 575.83 | 71.78 |
| **16:00** | 50.81 | 209.20 | 505.78 | 50.81 |
| **17:00** | 19.99 | 73.48 | 364.18 | 19.99 |
| **18:00** | 0.00 | 0.00 | 0.00 | 0.00 |
| **19:00** | 0.00 | 0.00 | 0.00 | 0.00 |
| **20:00** | 0.00 | 0.00 | 0.00 | 0.00 |
| **21:00** | 0.00 | 0.00 | 0.00 | 0.00 |
| **22:00** | 0.00 | 0.00 | 0.00 | 0.00 |
| **23:00** | 0.00 | 0.00 | 0.00 | 0.00 |

**Table 52** Hourly Solar Radiation [W/m^2^] Versus Orientation - August - Slope 90°

| **Hour** | **North** | **South** | **West** | **East** |
| --- | --- | --- | --- | --- |
| **00:00** | 0.00 | 0.00 | 0.00 | 0.00 |
| **01:00** | 0.00 | 0.00 | 0.00 | 0.00 |
| **02:00** | 0.00 | 0.00 | 0.00 | 0.00 |
| **03:00** | 0.00 | 0.00 | 0.00 | 0.00 |
| **04:00** | 0.00 | 0.00 | 0.00 | 0.00 |
| **05:00** | 115.84 | 31.74 | 31.74 | 466.39 |
| **06:00** | 92.97 | 77.98 | 51.37 | 726.90 |
| **07:00** | 62.90 | 214.28 | 62.90 | 771.06 |
| **08:00** | 71.52 | 339.52 | 71.52 | 688.73 |
| **09:00** | 79.49 | 435.43 | 79.49 | 525.42 |
| **10:00** | 90.07 | 484.36 | 90.07 | 316.29 |
| **11:00** | 101.42 | 469.15 | 240.35 | 101.42 |
| **12:00** | 97.69 | 417.95 | 412.79 | 97.69 |
| **13:00** | 84.54 | 360.69 | 593.17 | 84.54 |
| **14:00** | 72.17 | 246.57 | 675.31 | 72.17 |
| **15:00** | 56.02 | 114.25 | 682.49 | 54.71 |
| **16:00** | 106.74 | 40.42 | 617.03 | 40.42 |
| **17:00** | 73.41 | 11.09 | 243.13 | 11.09 |
| **18:00** | 0.00 | 0.00 | 0.00 | 0.00 |
| **19:00** | 0.00 | 0.00 | 0.00 | 0.00 |
| **20:00** | 0.00 | 0.00 | 0.00 | 0.00 |
| **21:00** | 0.00 | 0.00 | 0.00 | 0.00 |
| **22:00** | 0.00 | 0.00 | 0.00 | 0.00 |
| **23:00** | 0.00 | 0.00 | 0.00 | 0.00 |

## **A.17 Ambient Temperature [**$\boldsymbol{C^{\circ}}$**] – All Months**

**Table 53** Ambient Temperature [$C^{\circ}$] Versus Months

| **Hour** | **November** | **January** | **March** | **August** |
| --- | --- | --- | --- | --- |
| **00:00** | 19.08 | 12.22 | 15.15 | 26.55 |
| **01:00** | 19.05 | 12.10 | 14.98 | 26.36 |
| **02:00** | 19.01 | 11.98 | 14.81 | 26.17 |
| **03:00** | 18.97 | 11.87 | 14.63 | 25.98 |
| **04:00** | 19.10 | 11.92 | 14.98 | 26.73 |
| **05:00** | 19.23 | 11.96 | 15.31 | 27.48 |
| **06:00** | 19.35 | 12.01 | 15.65 | 28.23 |
| **07:00** | 19.98 | 12.47 | 16.42 | 28.59 |
| **08:00** | 20.61 | 12.93 | 17.19 | 28.95 |
| **09:00** | 21.24 | 13.39 | 17.95 | 29.31 |
| **10:00** | 21.44 | 13.55 | 18.07 | 29.32 |
| **11:00** | 21.66 | 13.72 | 18.19 | 29.33 |
| **12:00** | 21.86 | 13.89 | 18.31 | 29.35 |
| **13:00** | 21.23 | 13.58 | 17.94 | 29.31 |
| **14:00** | 20.60 | 13.28 | 17.59 | 29.27 |
| **15:00** | 19.98 | 12.97 | 17.22 | 29.24 |
| **16:00** | 19.82 | 12.85 | 16.69 | 28.80 |
| **17:00** | 19.67 | 12.73 | 16.16 | 28.37 |
| **18:00** | 19.51 | 12.60 | 15.63 | 27.93 |
| **19:00** | 19.32 | 12.46 | 15.45 | 27.56 |
| **20:00** | 19.13 | 12.32 | 15.28 | 27.18 |
| **21:00** | 18.93 | 12.17 | 15.10 | 26.81 |
| **22:00** | 18.90 | 12.25 | 15.10 | 26.71 |
| **23:00** | 18.88 | 12.33 | 15.09 | 26.60 |

## **B.1 Fluid Average Temperature – Configuration Comparison - Slope 0°**

| **Graph – November – Slope 0°** |
| --- |
| 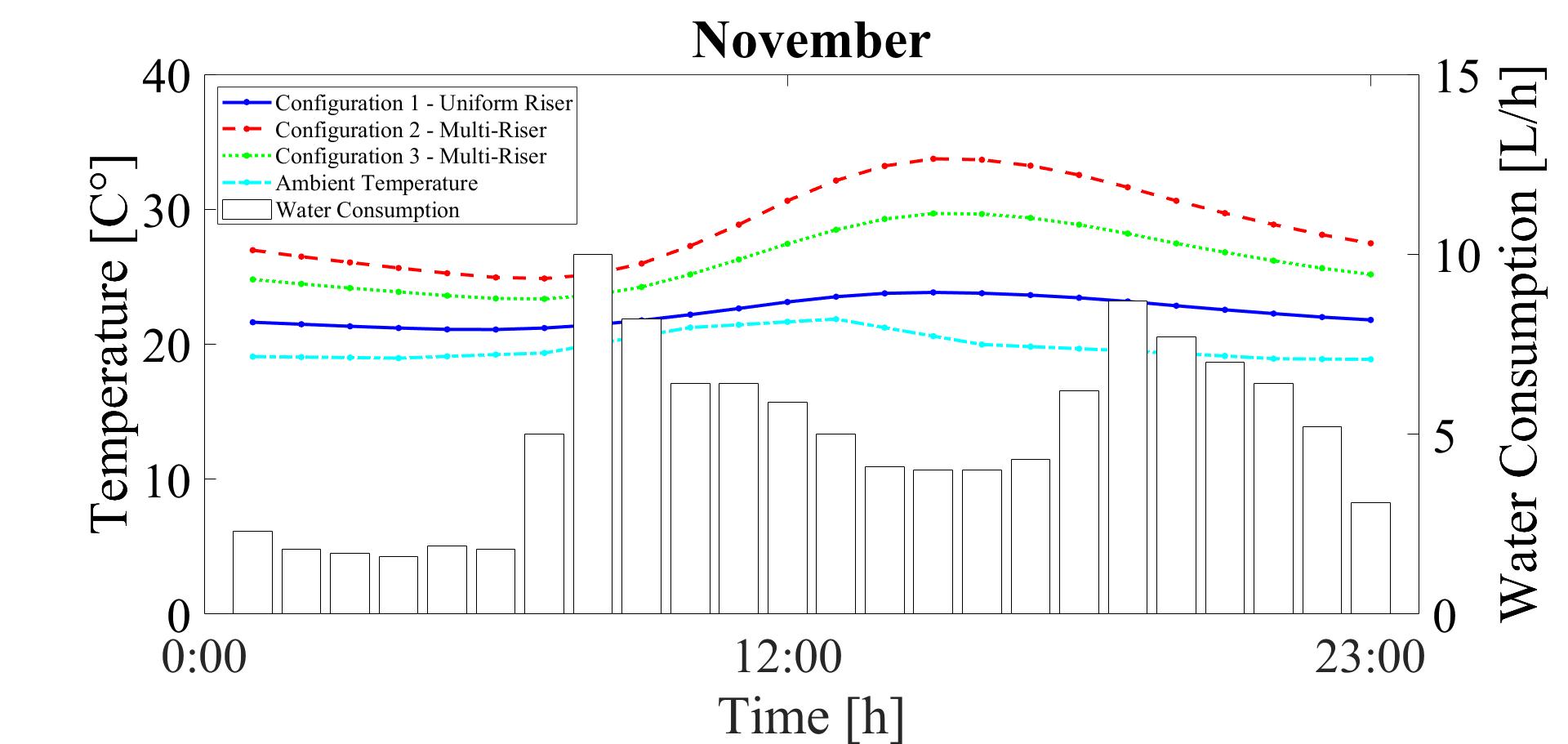 |
| **Graph – January - Slope 0°** |
| 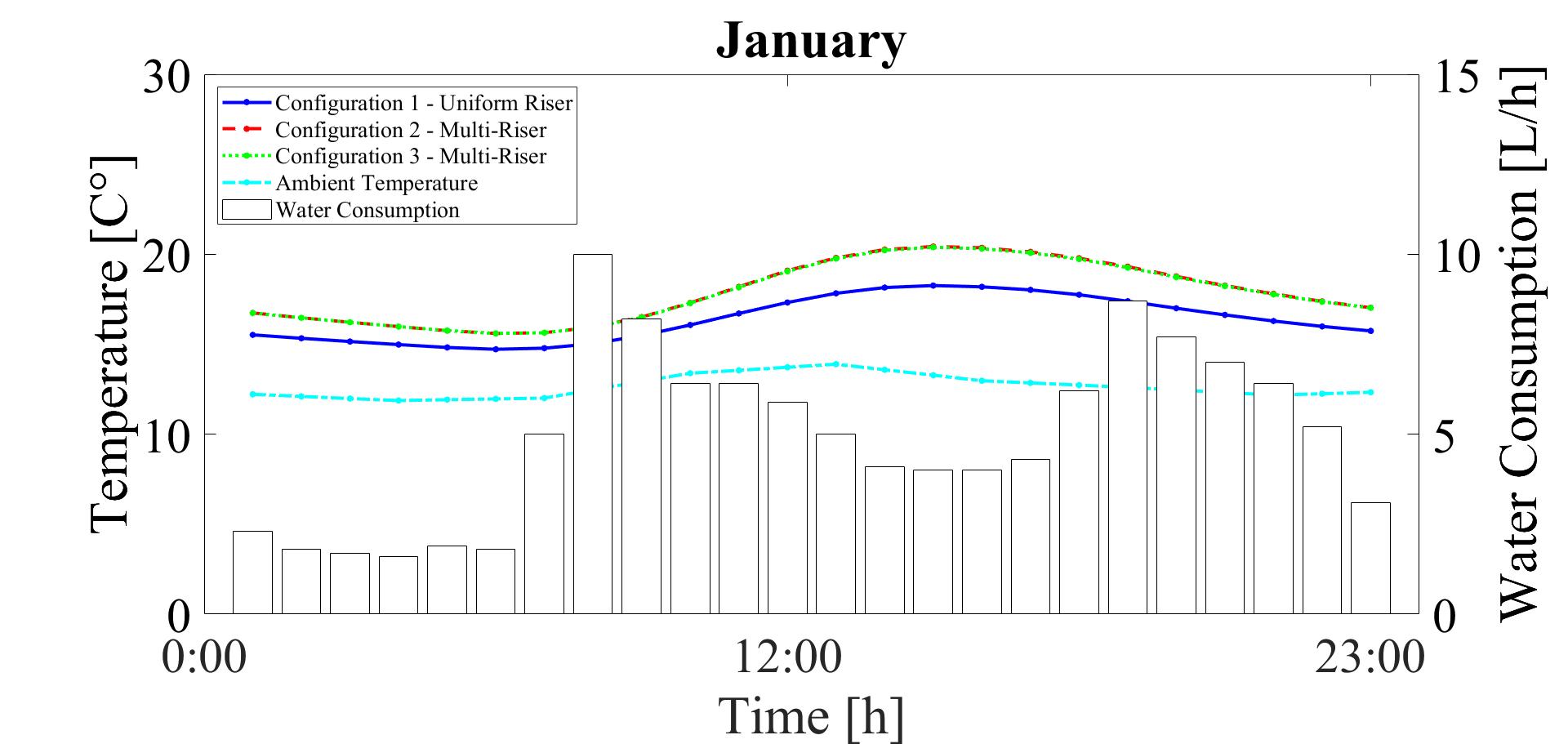 |

**Figure B1- 1** Fluid Average Temperature –System comparison - November and January – Slope 0°

| **Graph – March – Slope 0°** |
| --- |
| 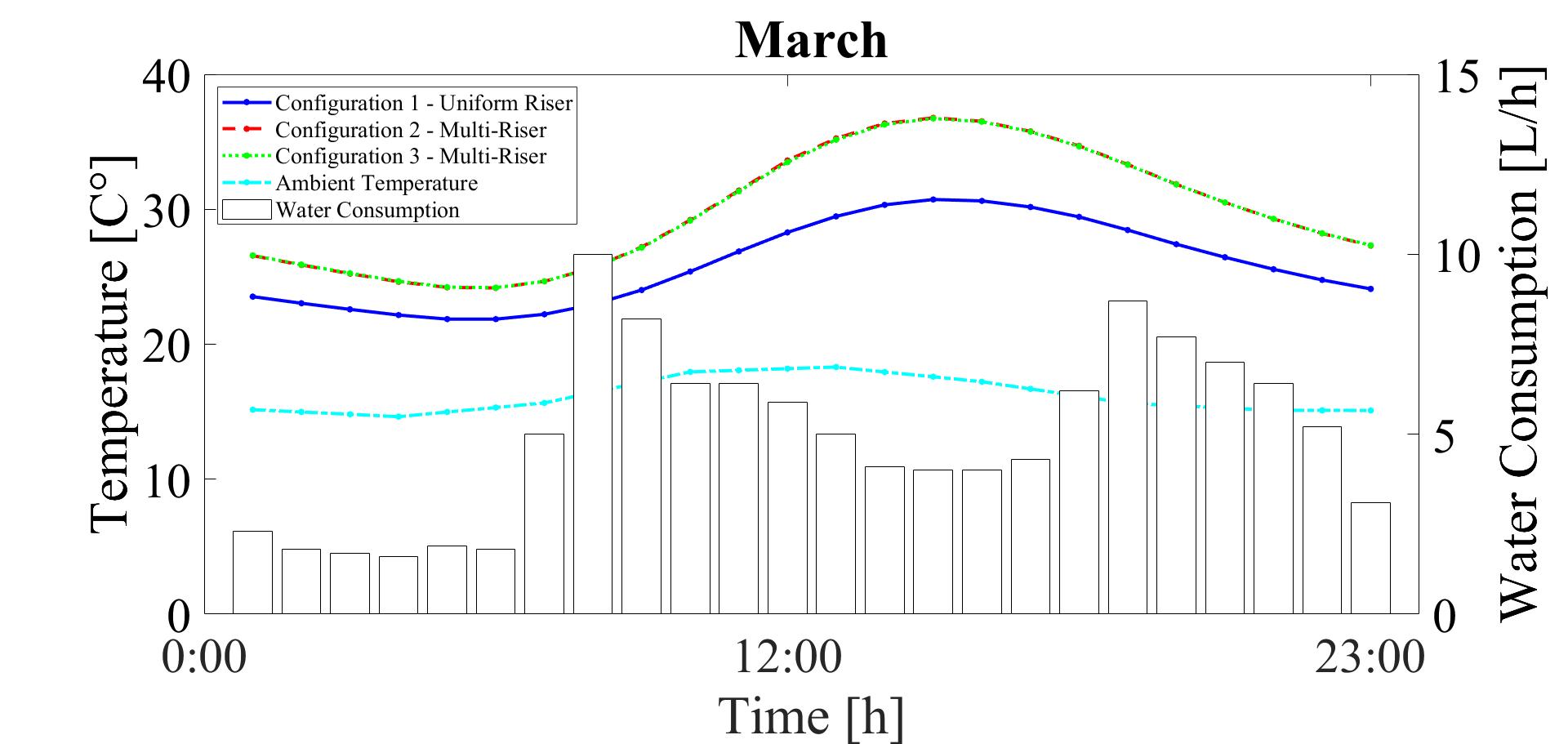 |
| **Graph – August - Slope 0°** |
| 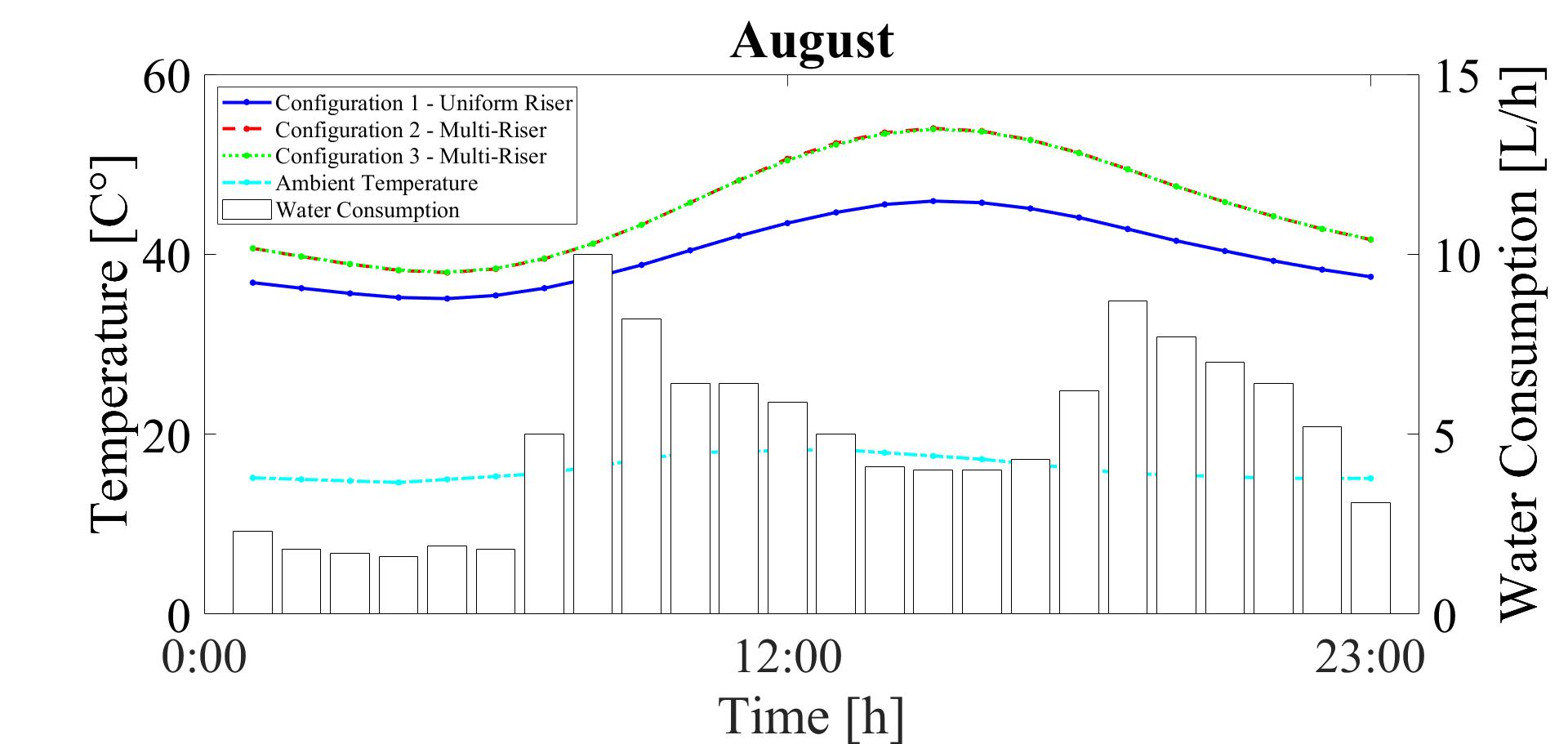 |

**Figure B1-2** Fluid Average Temperature –System comparison - March and August – Slope 0°

## **B.2 Fluid Average Temperature - Configuration Comparison - Slope 45°**

| **Graph – November - North – Slope 45°** |
| --- |
| 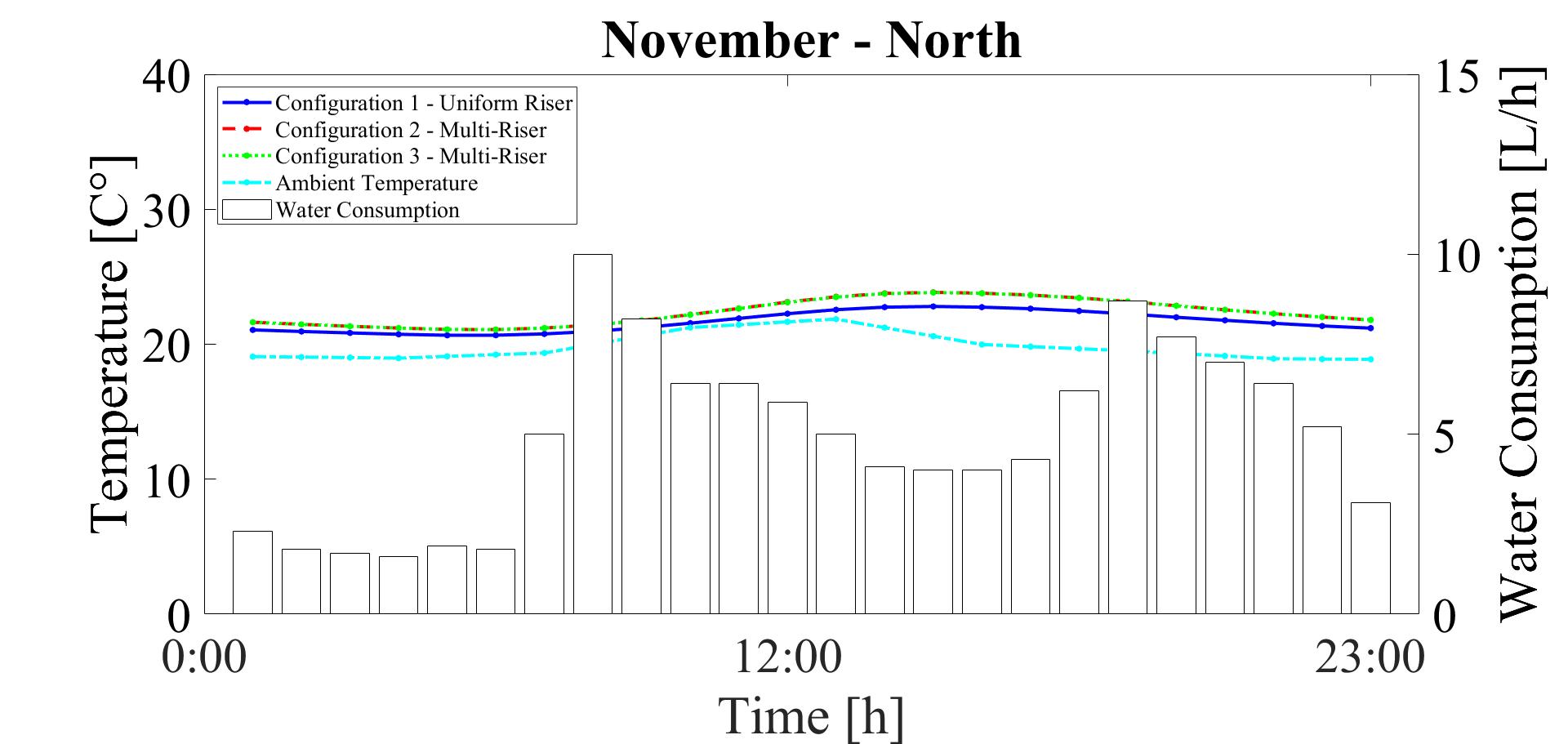 |
| **Graph – November - South – Slope 45°** |
| 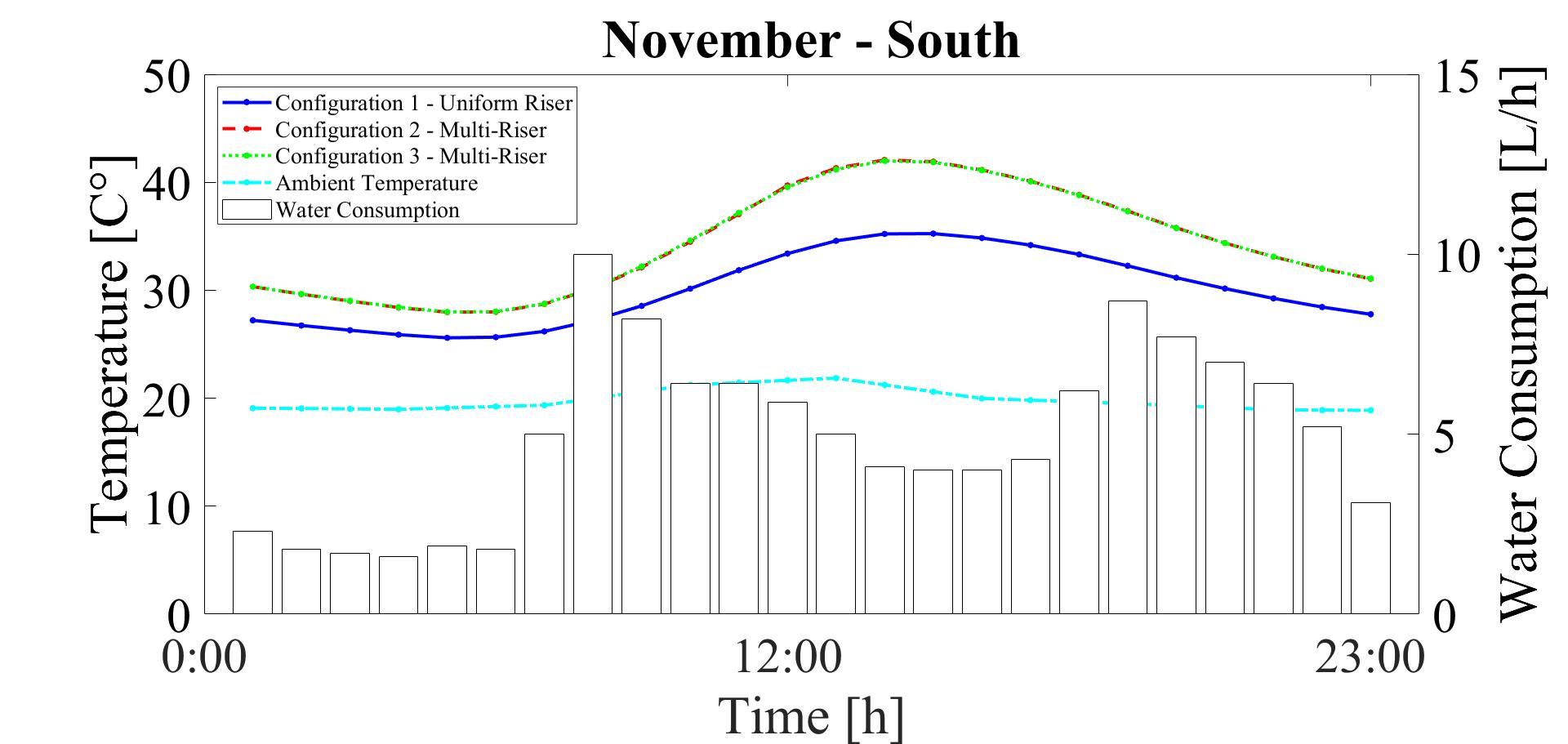 |

**Figure B2-1** Fluid Average Temperature - System comparison - November – North and South – Slope 45°

| **Graph – November - West – Slope 45°** |
| --- |
| 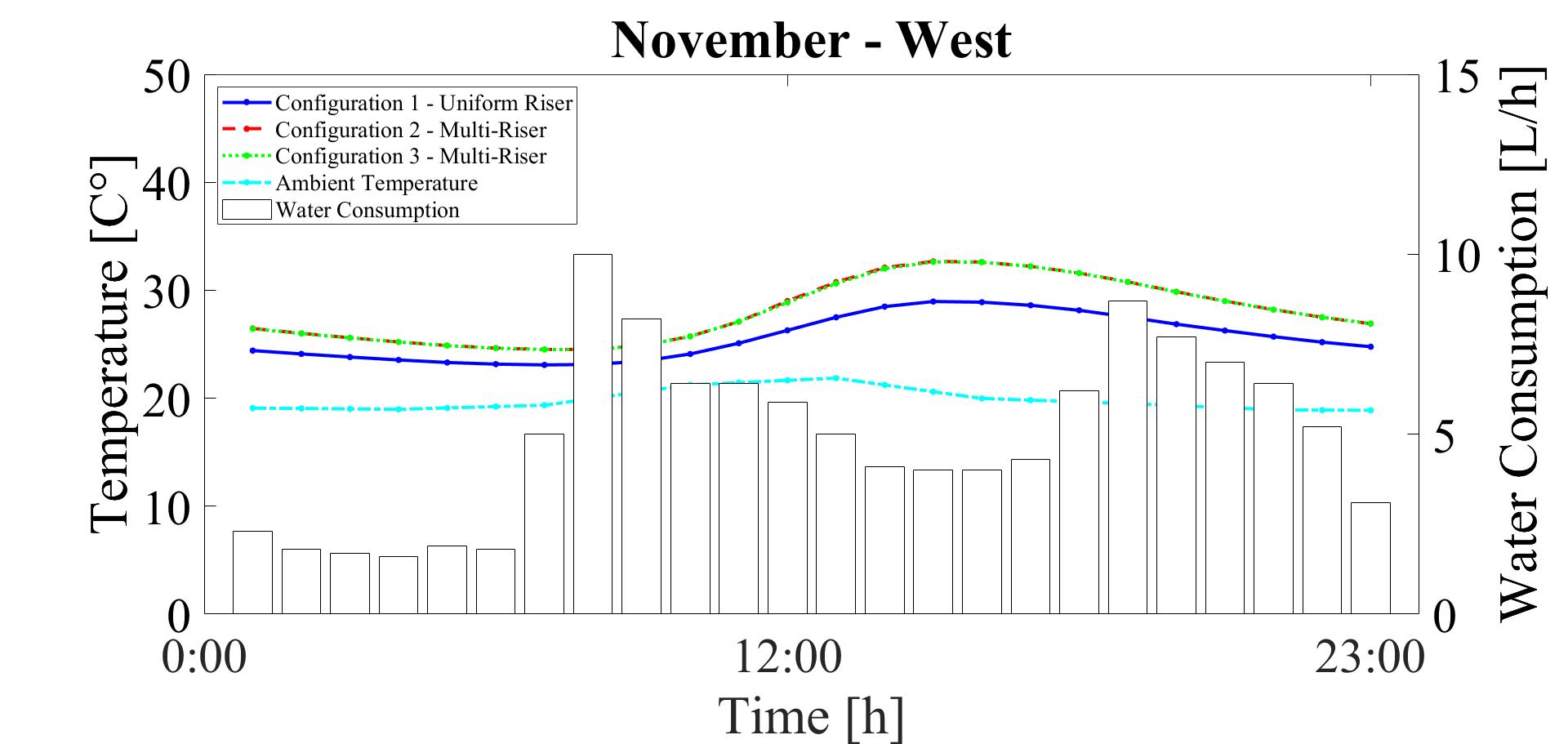 |
| **Graph – November - East – Slope 45°** |
| 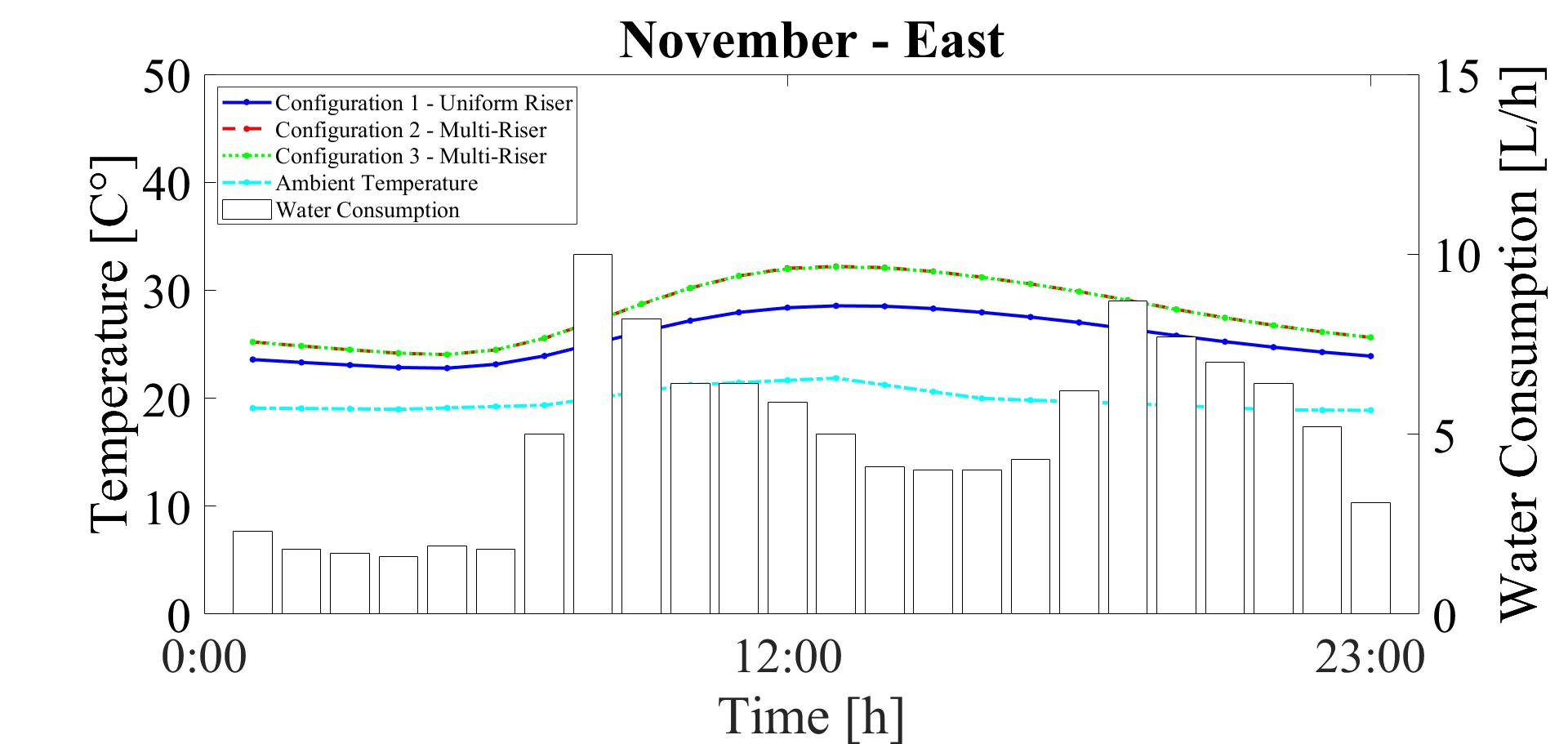 |

**Figure B2-2** Fluid Average Temperature - System comparison - November – West and East – Slope 45°

| **Graph – January - North – Slope 45°** |
| --- |
| 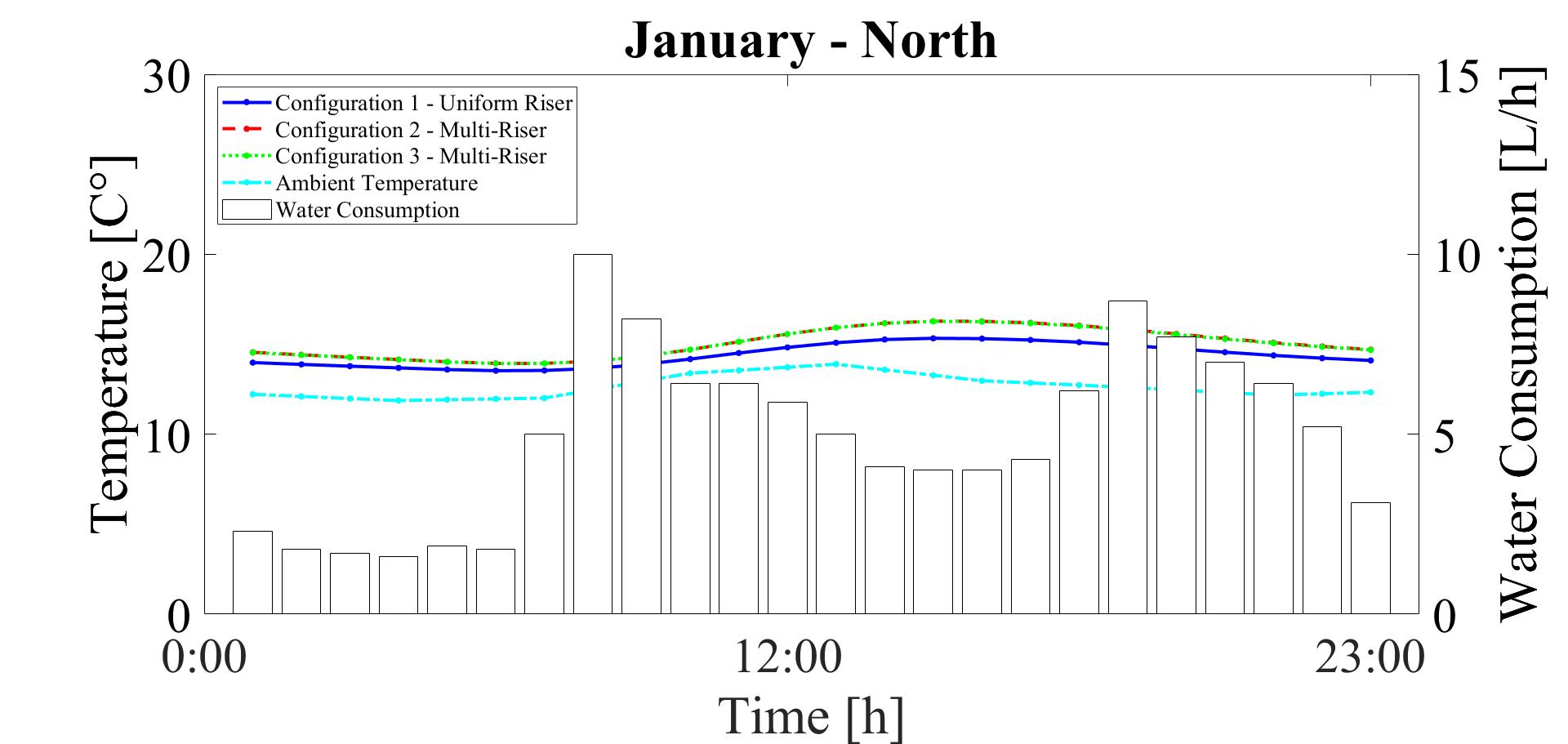 |
| **Graph – January - South – Slope 45°** |
| 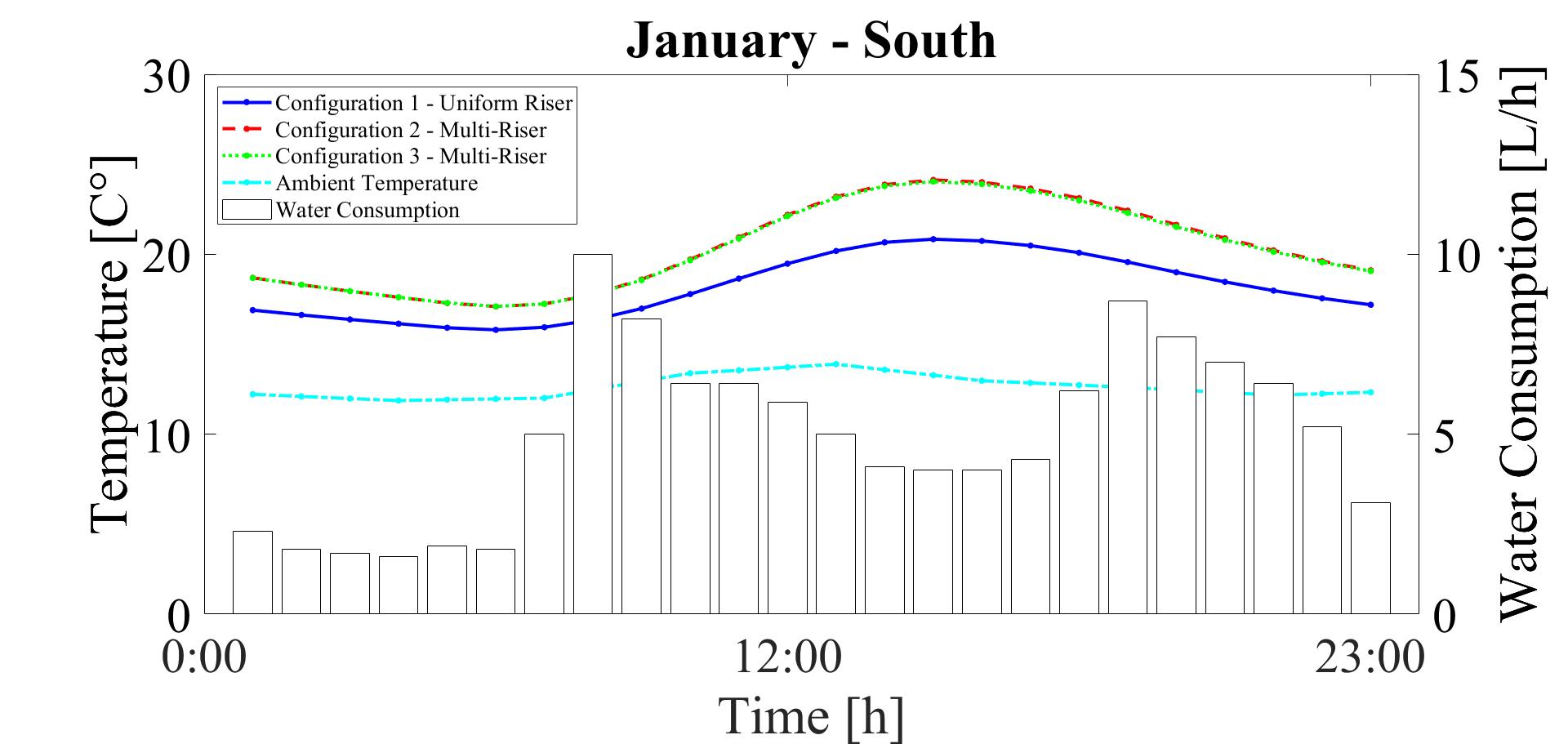 |

**Figure B2-3** Fluid Average Temperature - System comparison - January – North and South – Slope 45°

| **Graph – January - West – Slope 45°** |
| --- |
| 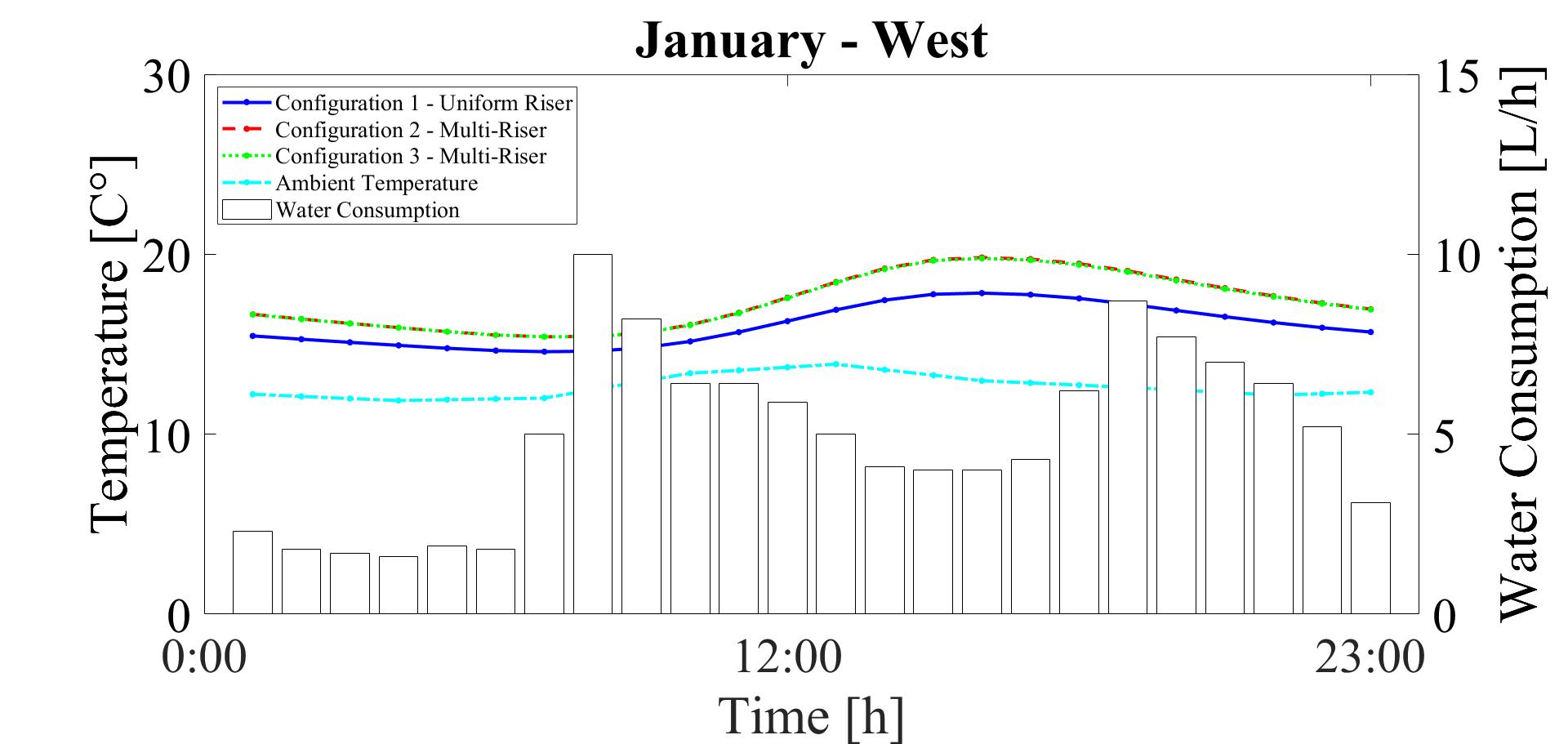 |
| **Graph – January - East – Slope 45°** |
| 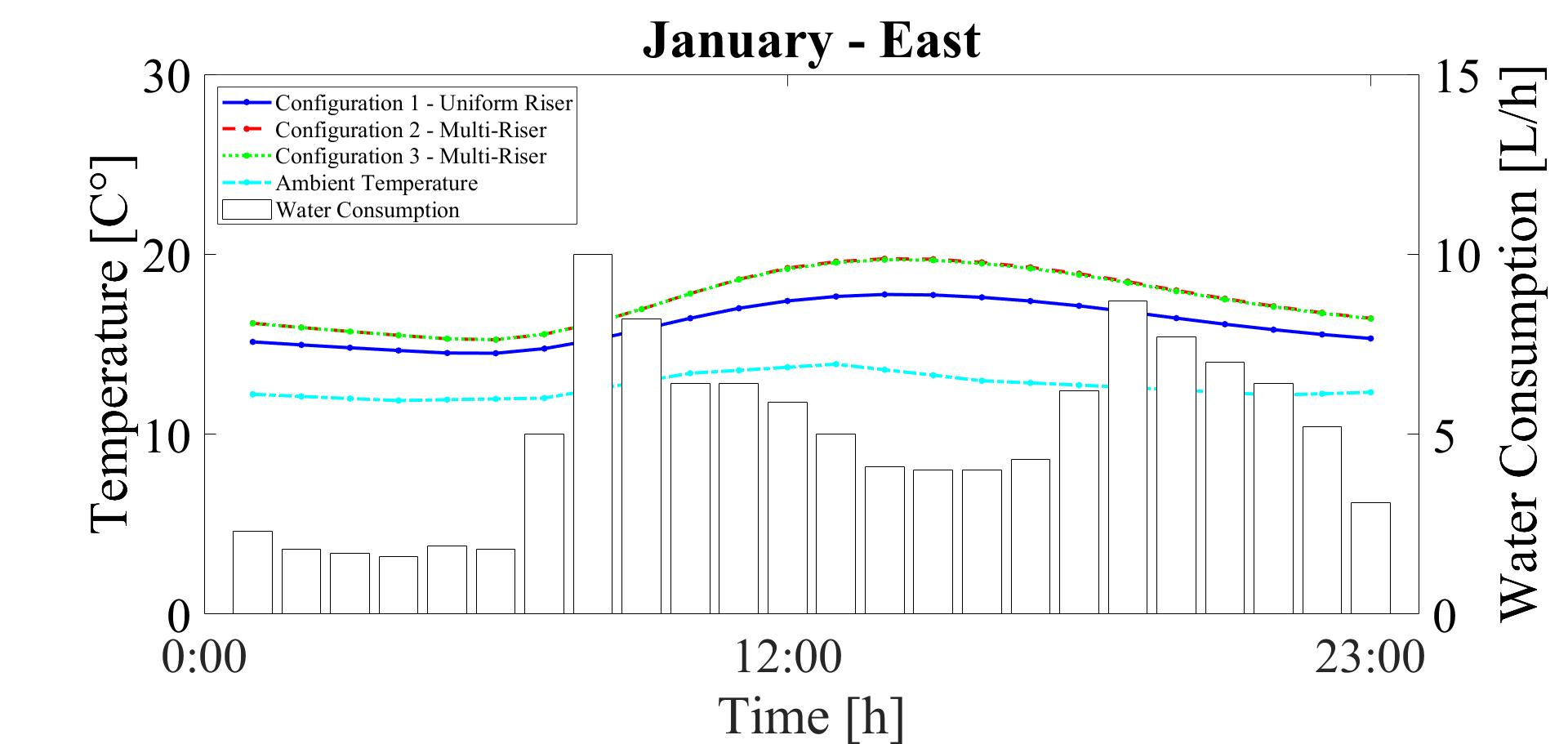 |

**Figure B2-4** Fluid Average Temperature - System comparison - January – West and East – Slope 45°

| **Graph – March - North – Slope 45°** |
| --- |
| 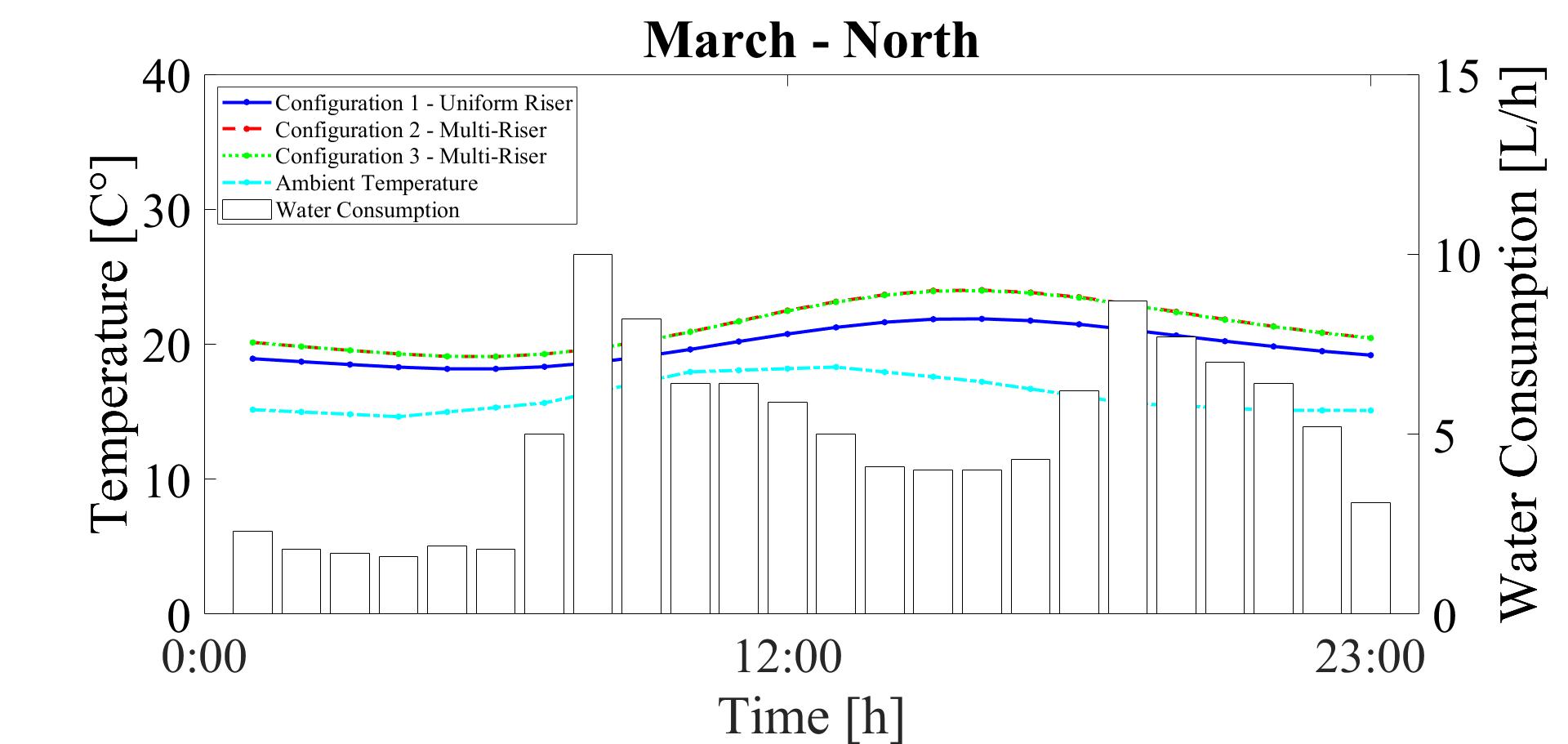 |
| **Graph – March - South – Slope 45°** |
| 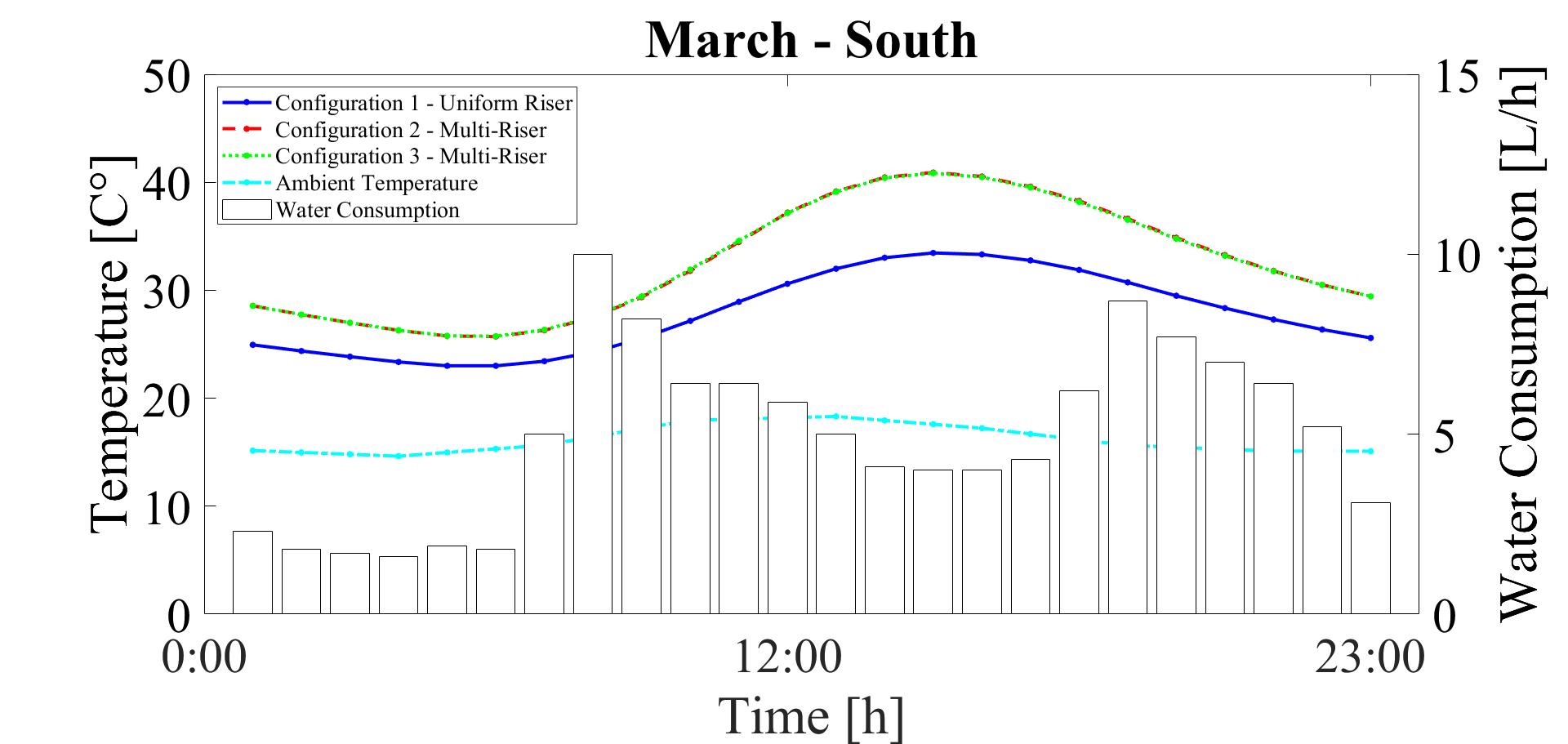 |

**Figure B2-5** Fluid Average Temperature - System comparison - March – North and South – Slope 45°

| **Graph – March - West – Slope 45°** |
| --- |
| 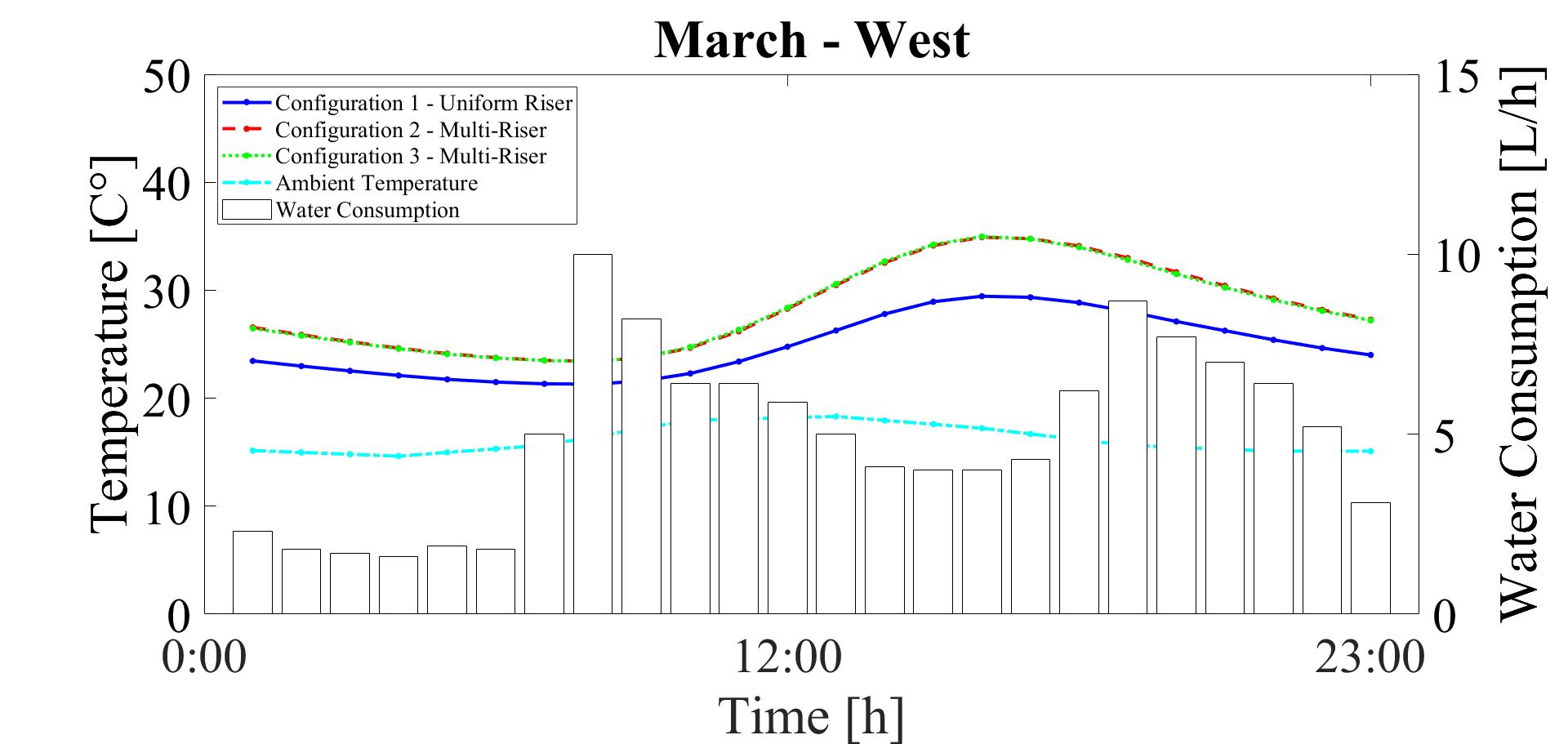 |
| **Graph – March - East – Slope 45°** |
| 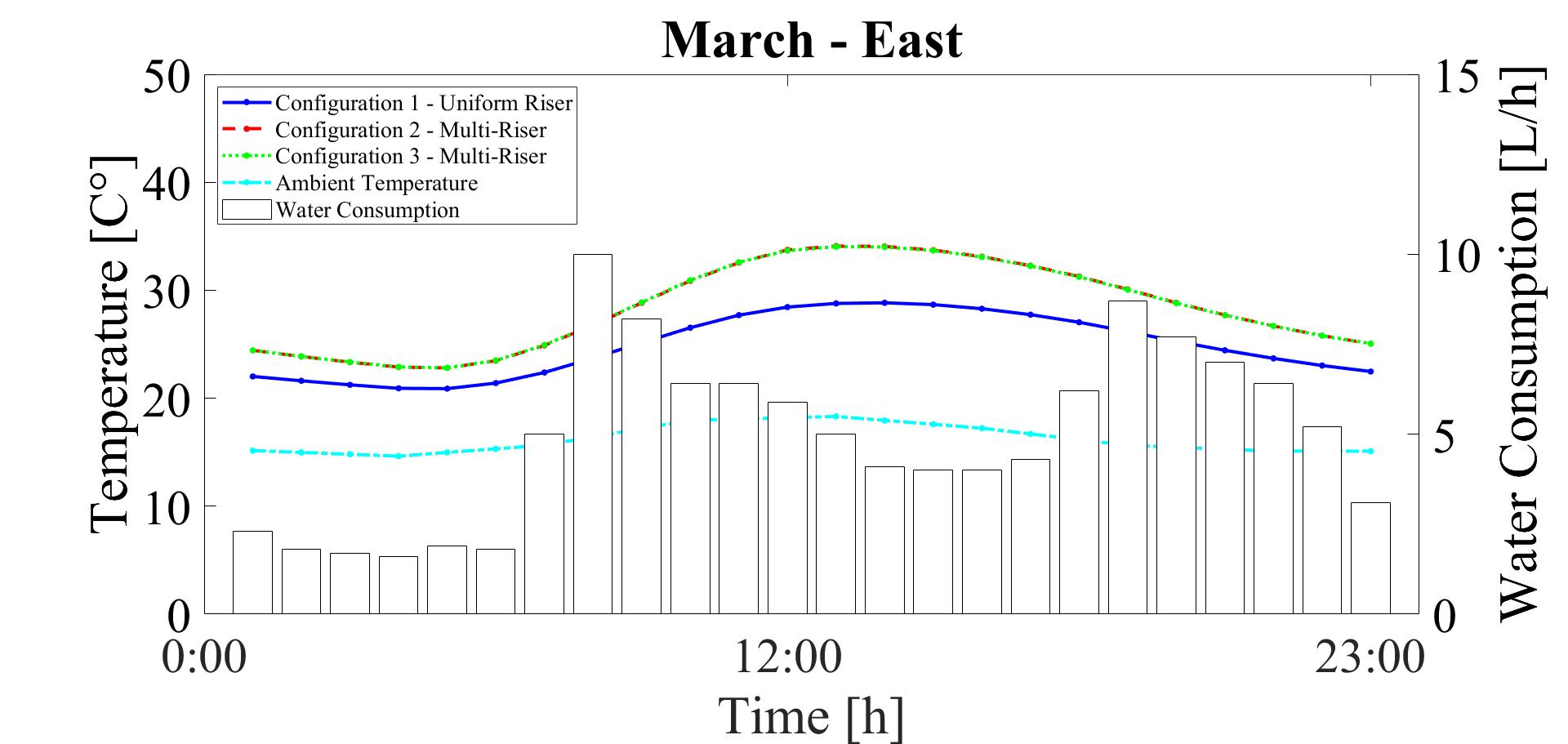 |

**Figure B2-6** Fluid Average Temperature - System comparison - March – West and East – Slope 45°

| **Graph – August - North – Slope 45°** |
| --- |
| 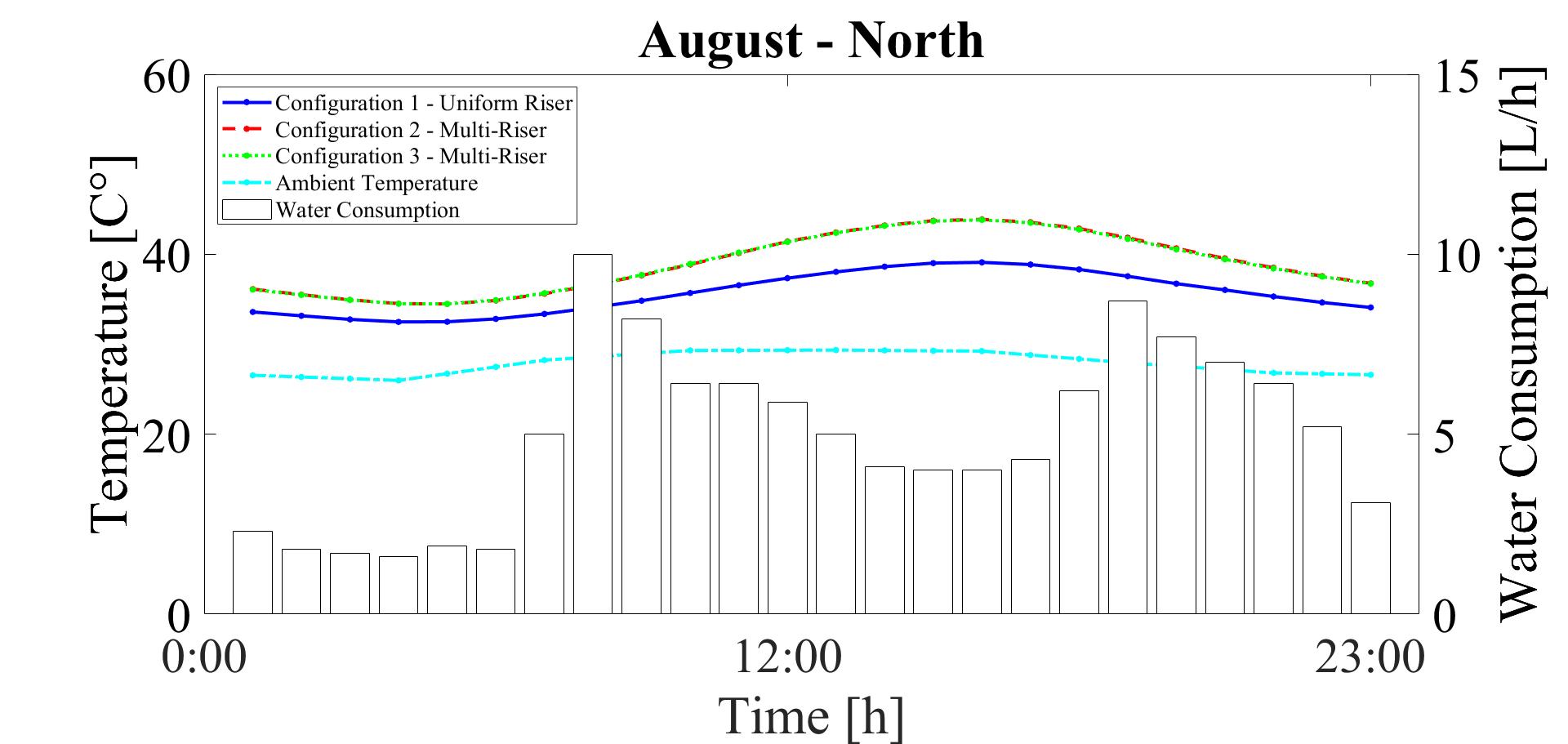 |
| **Graph – August - South – Slope 45°** |
| 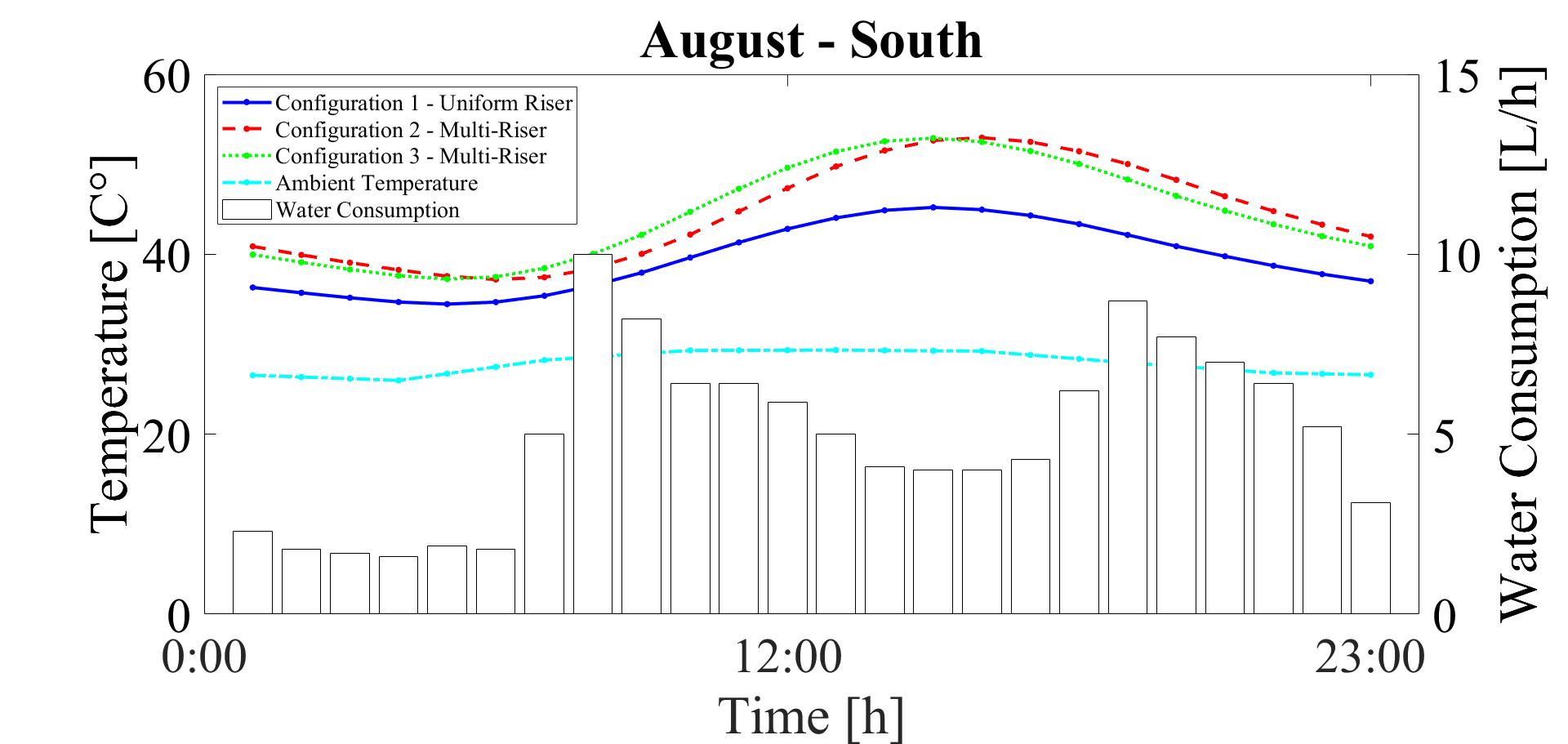 |

**Figure B2-7** Fluid Average Temperature - System comparison - August – North and South – Slope 45°

| **Graph – August - West – Slope 45°** |
| --- |
| 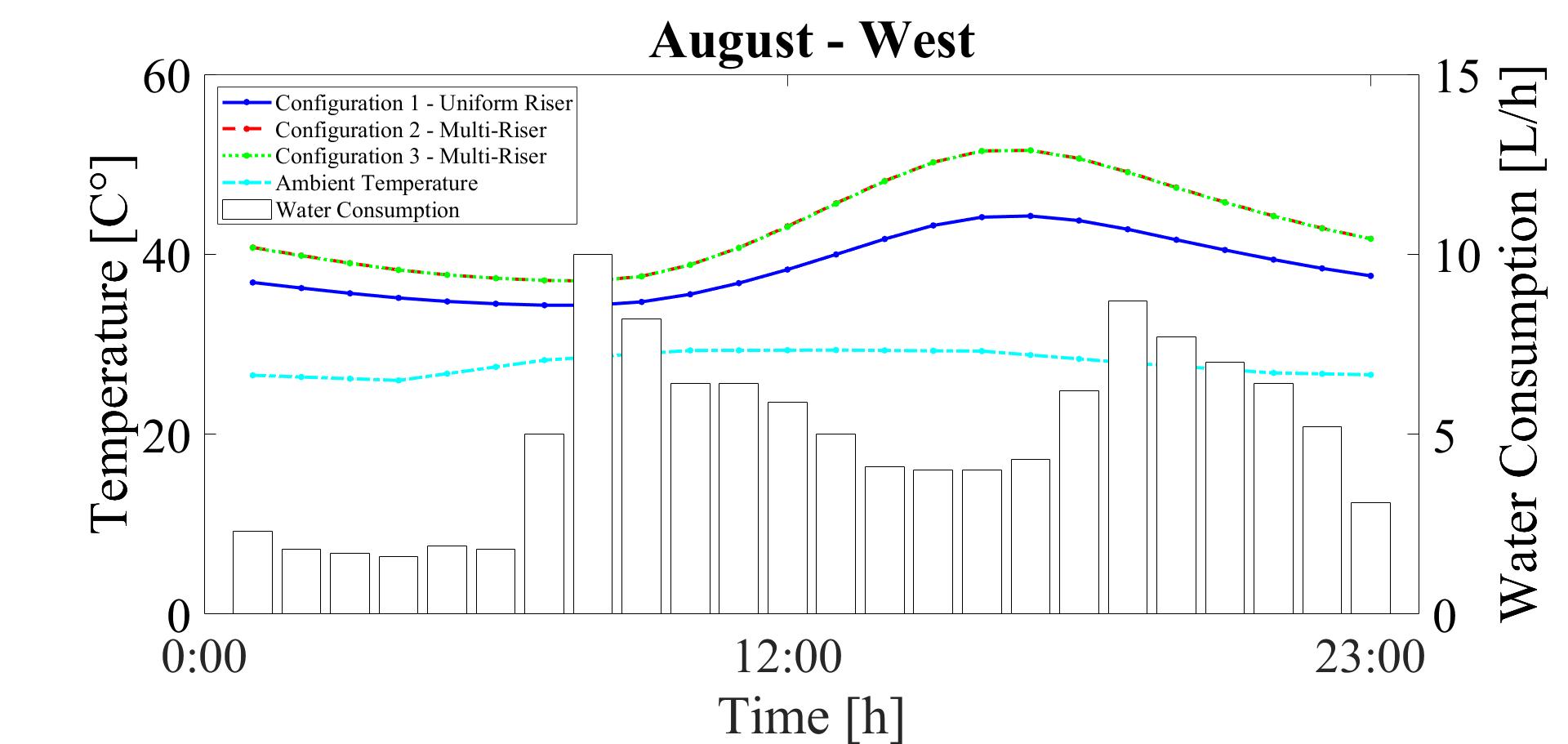 |
| **Graph – August - East – Slope 45°** |
| 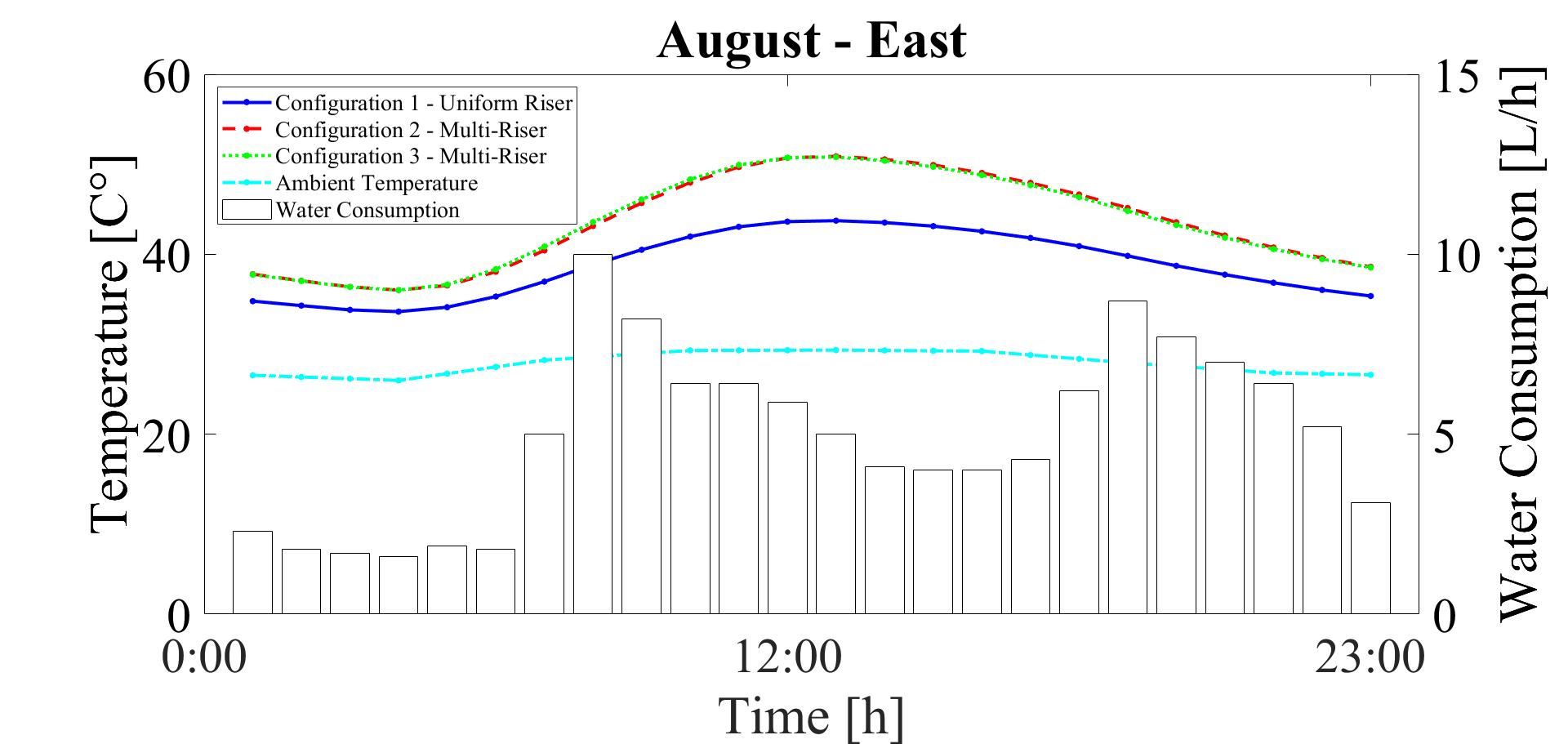 |

**Figure B2-8** Fluid Average Temperature - System comparison - August – West and East – Slope 45°

## **B.3 Fluid Average Temperature - Configuration Comparison - Slope 90°**

| **Graph – November - North – Slope 90°** |
| --- |
| 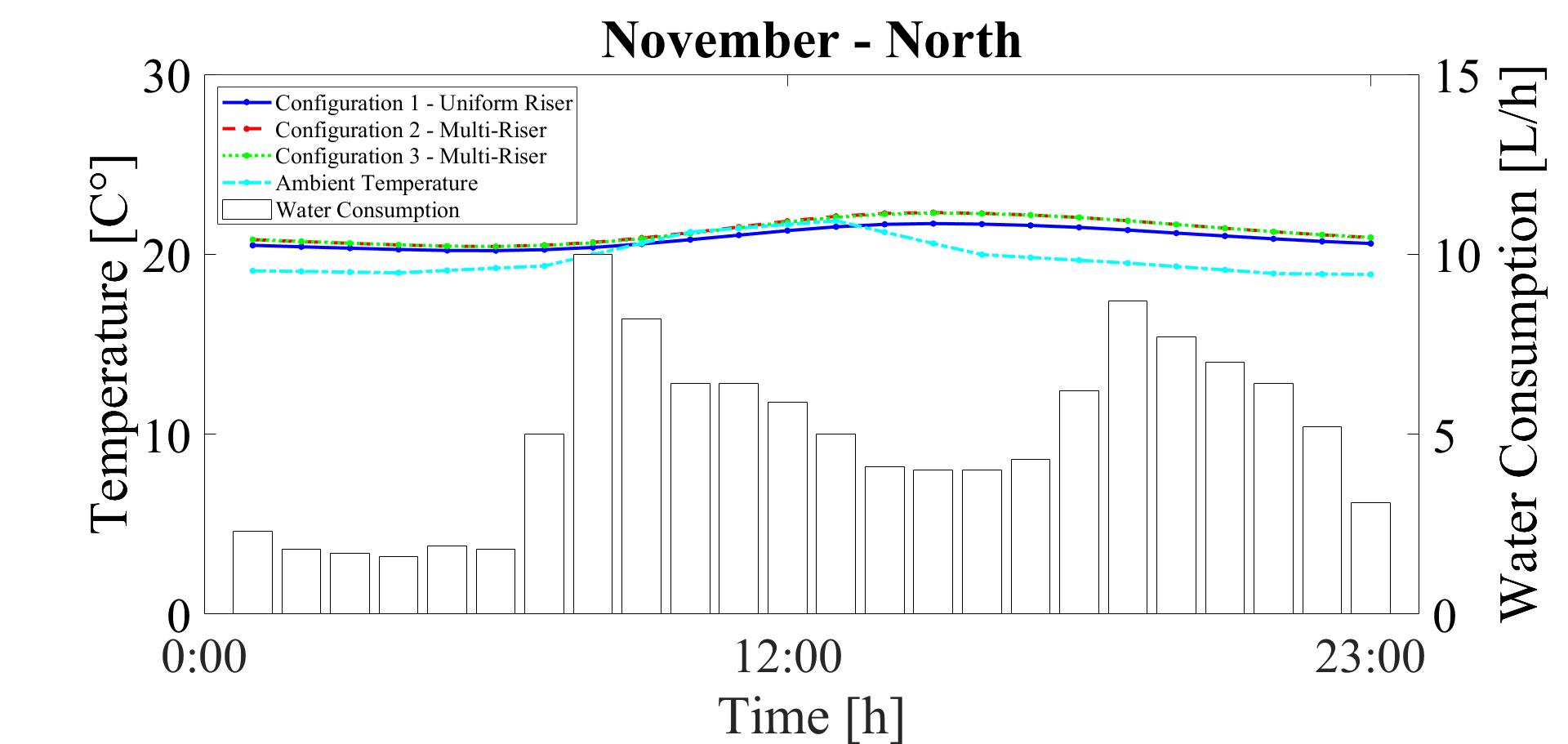 |
| **Graph – November - South – Slope 90°** |
| 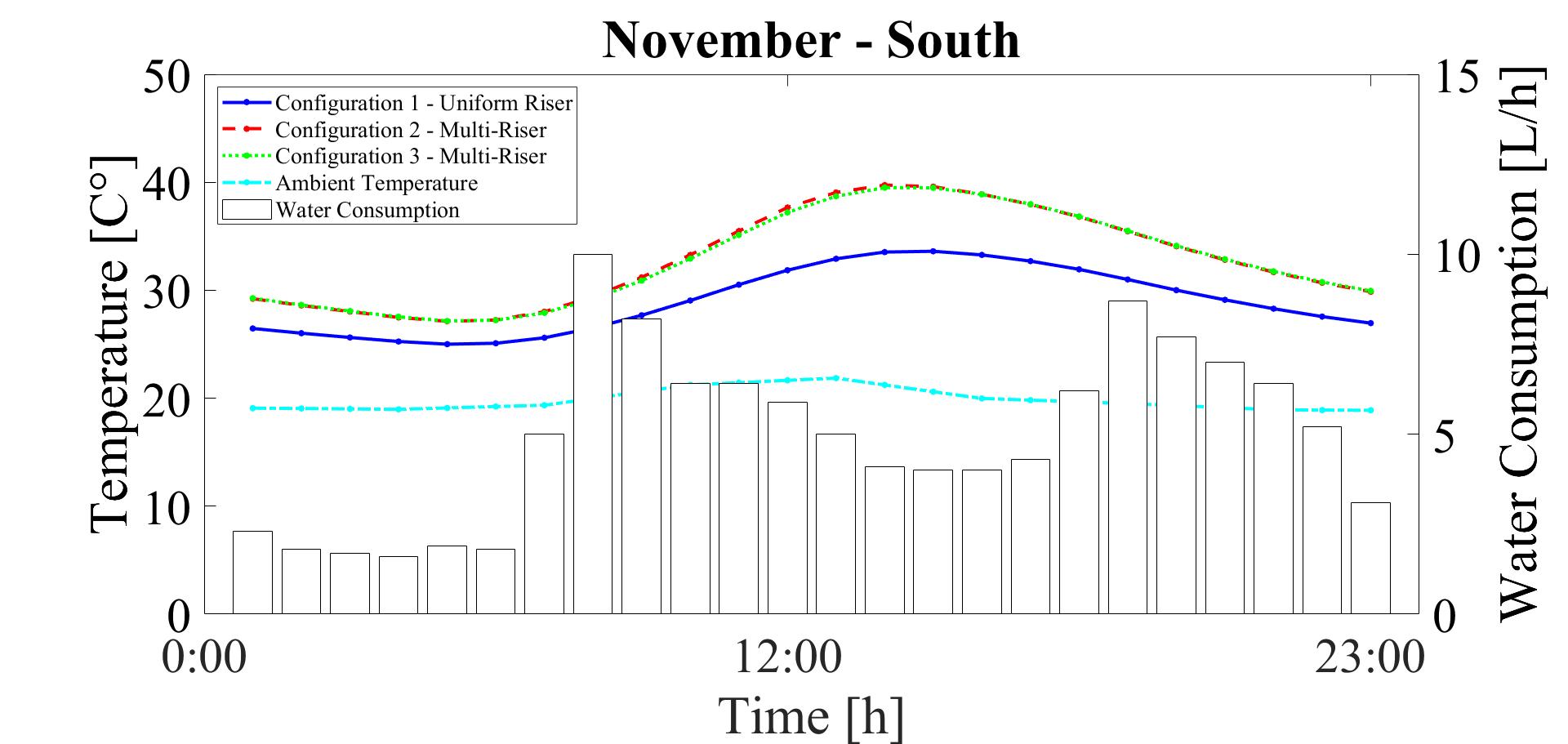 |

**Figure B3-1** Fluid Average Temperature - System comparison - November – North and South – Slope 90°

| **Graph – November - West – Slope 90°** |
| --- |
| 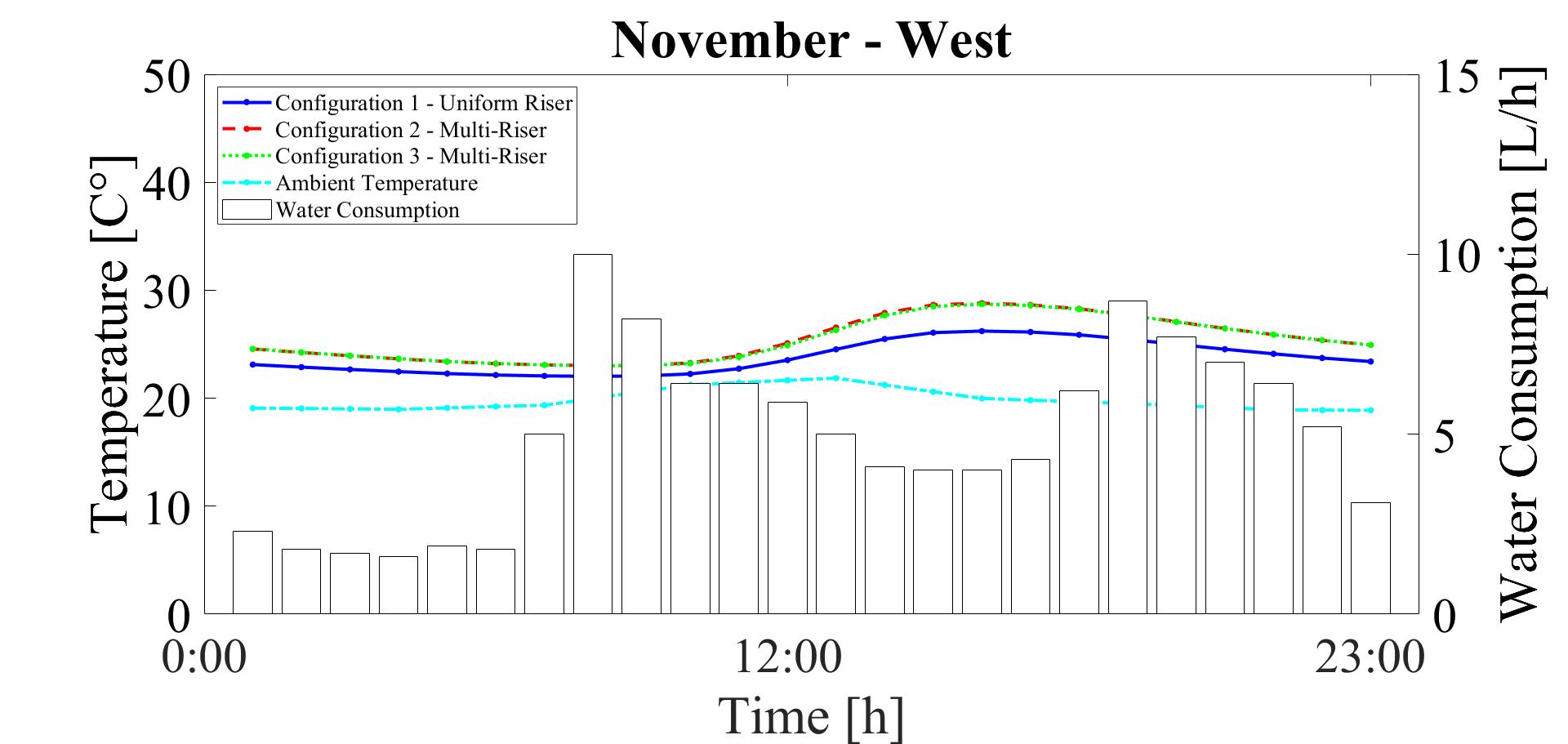 |
| **Graph – November - East – Slope 90°** |
| 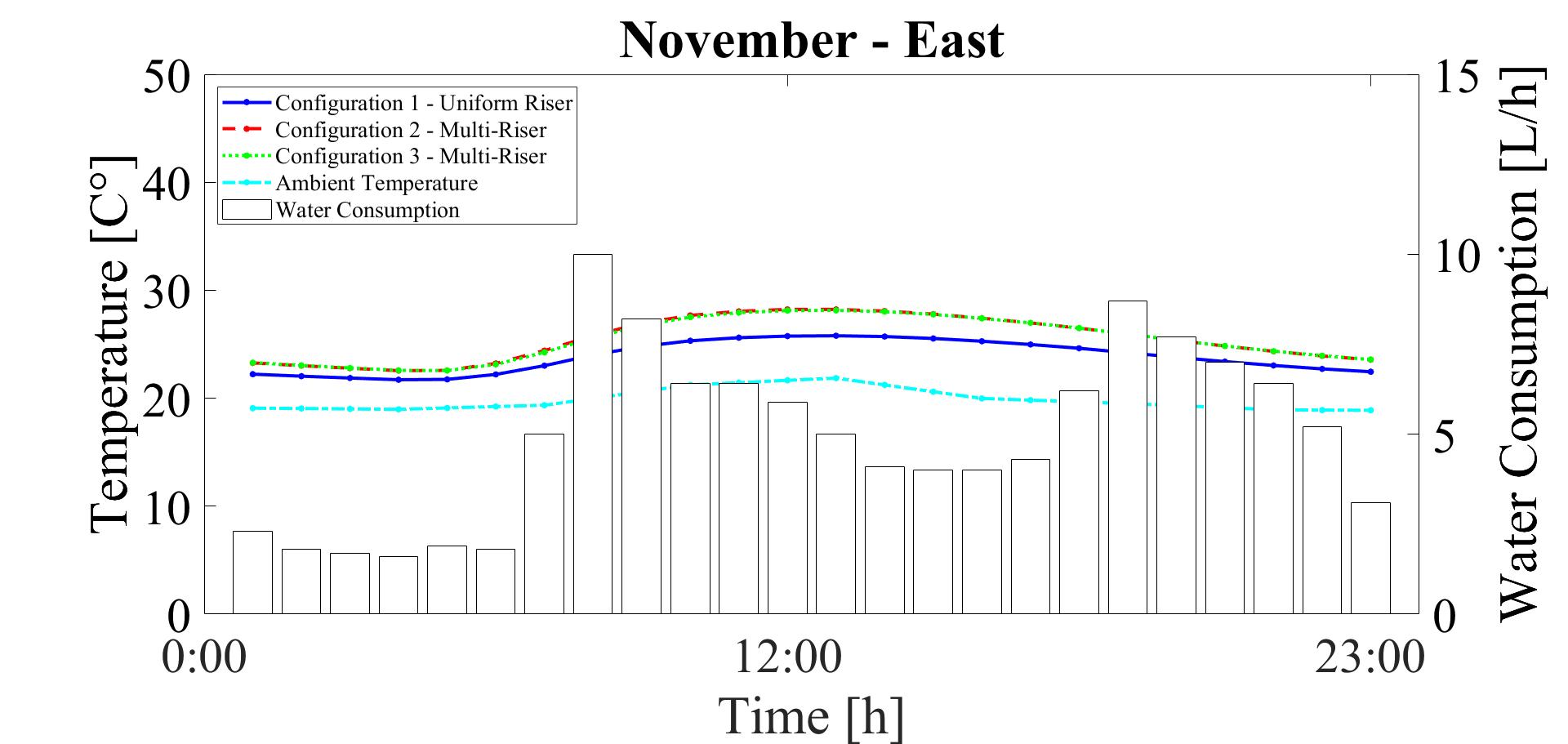 |

**Figure B3-2** Fluid Average Temperature - System comparison - November – West and East – Slope 90°

| **Graph – January - North – Slope 90°** |
| --- |
| 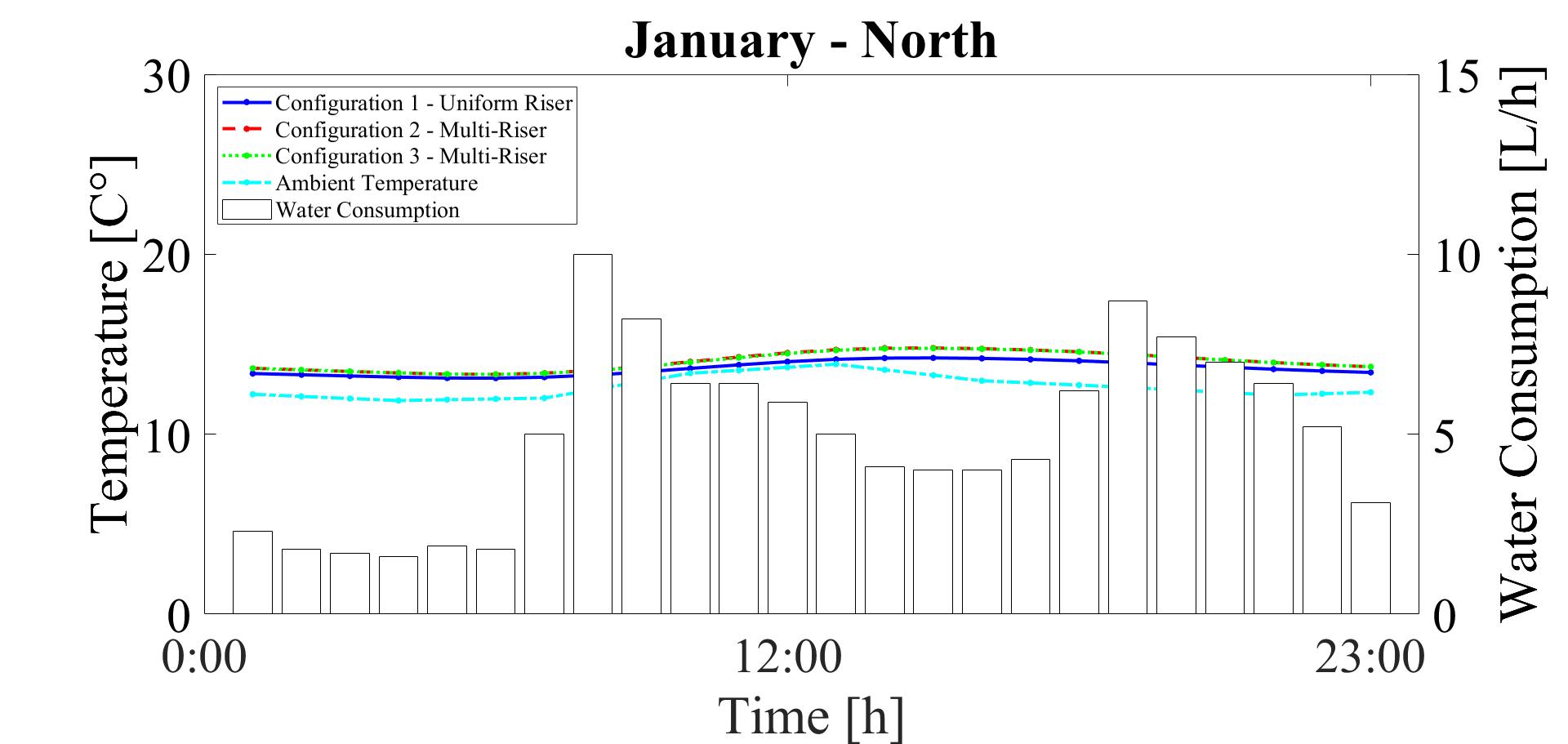 |
| **Graph – January - South – Slope 90°** |
| 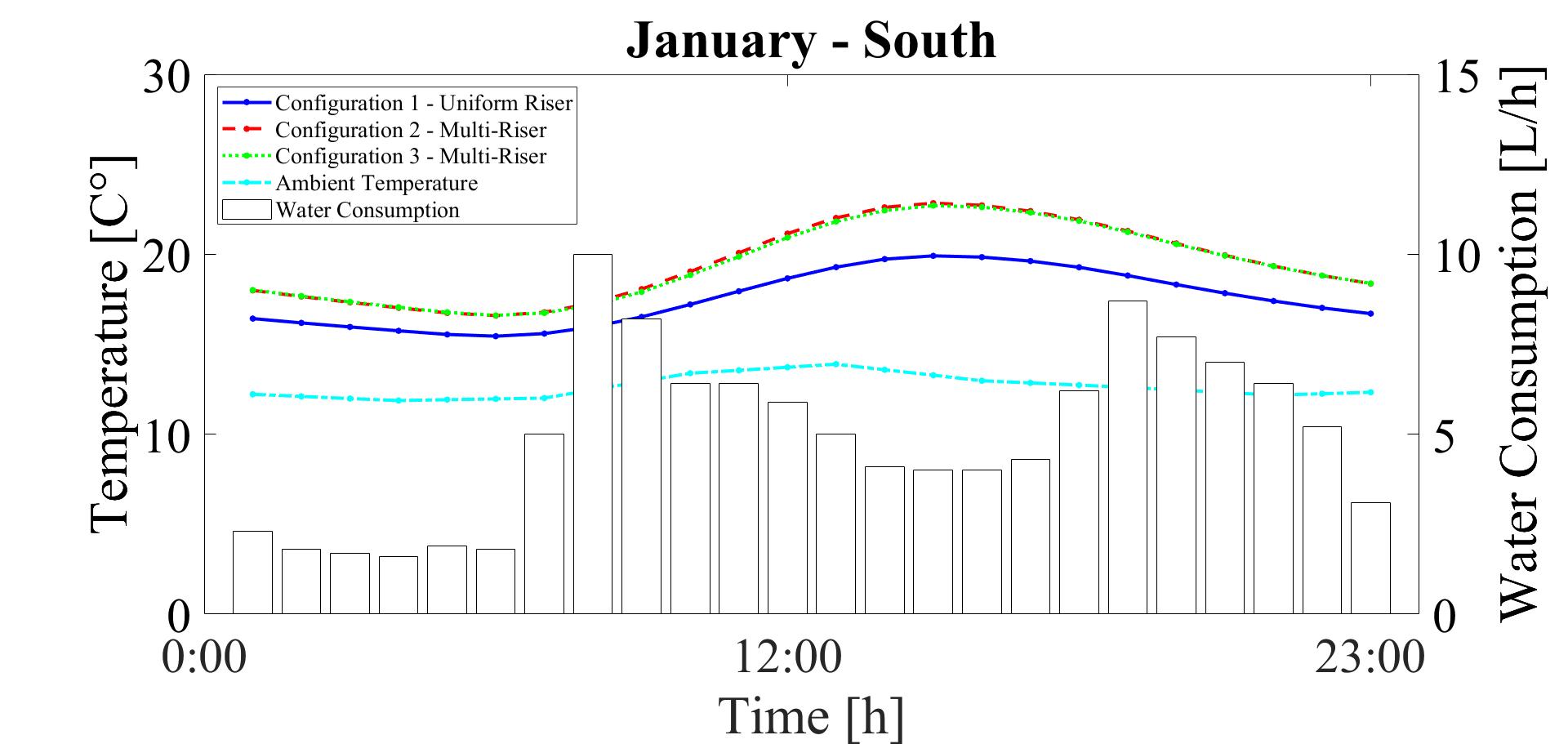 |

**Figure B3-3** Fluid Average Temperature - System comparison - January – North and South – Slope 90°

| **Graph – January - West – Slope 90°** |
| --- |
| 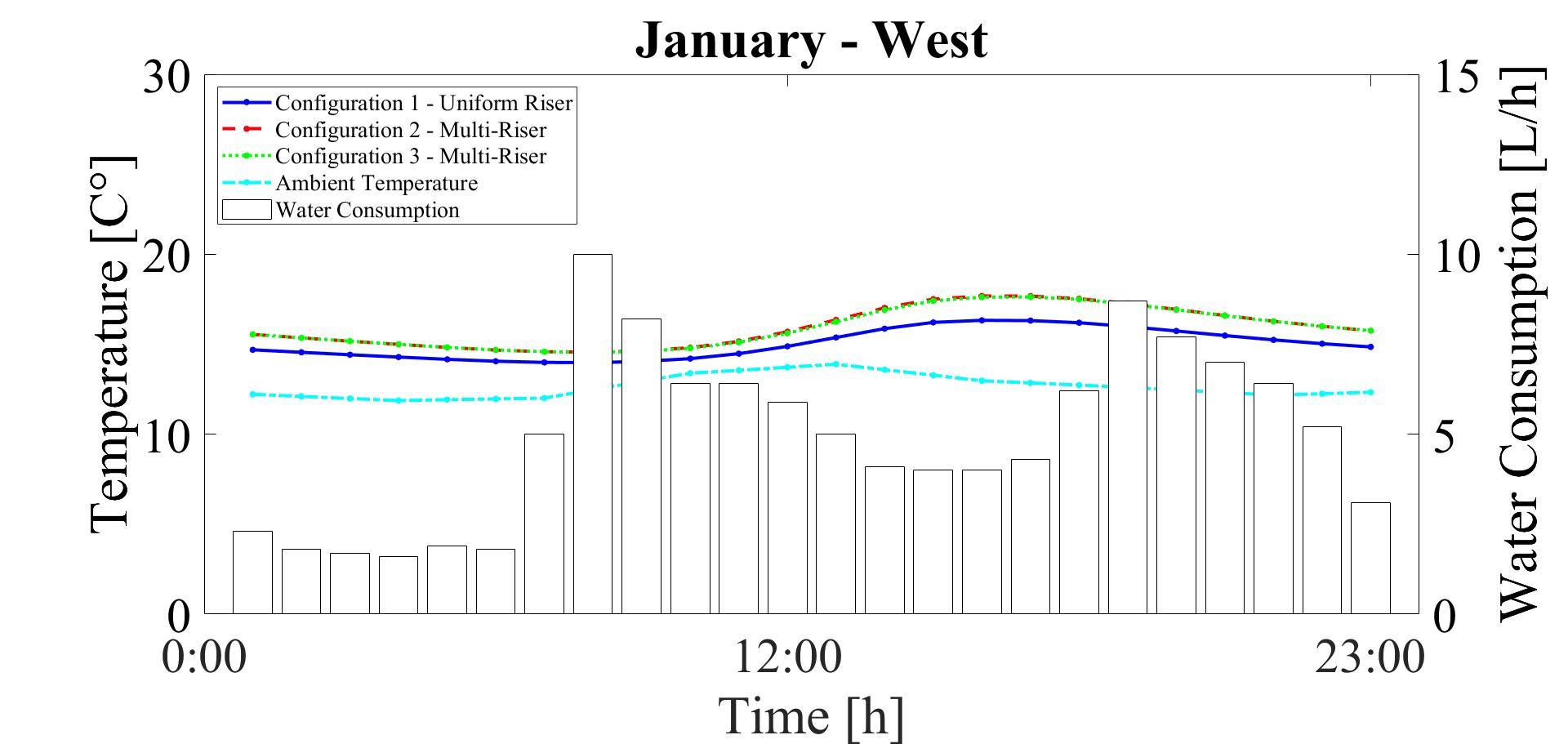 |
| **Graph – January - East – Slope 90°** |
| 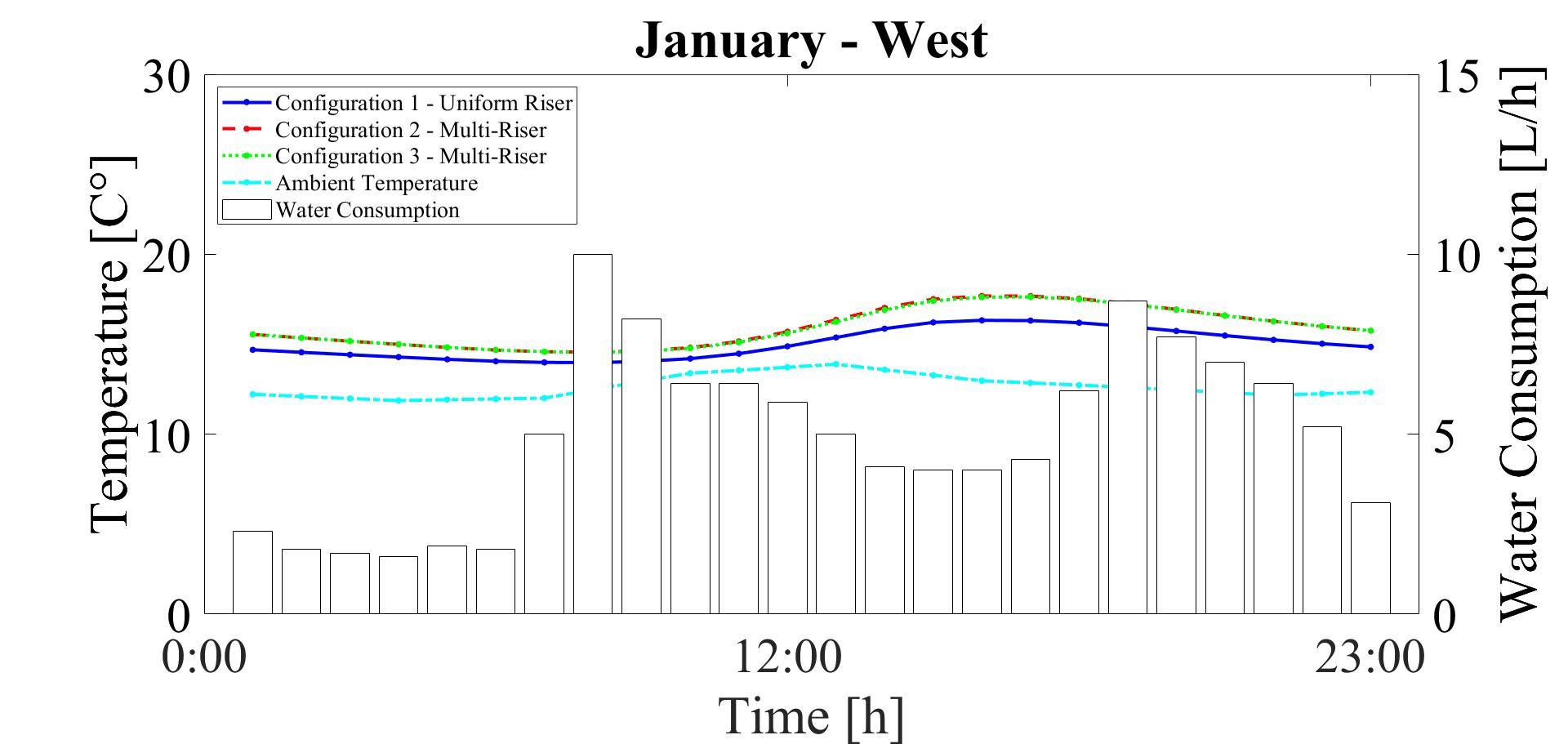 |

**Figure B3-4** Fluid Average Temperature - System comparison - January – West and East – Slope 90°

| **Graph – March - North – Slope 90°** |
| --- |
| 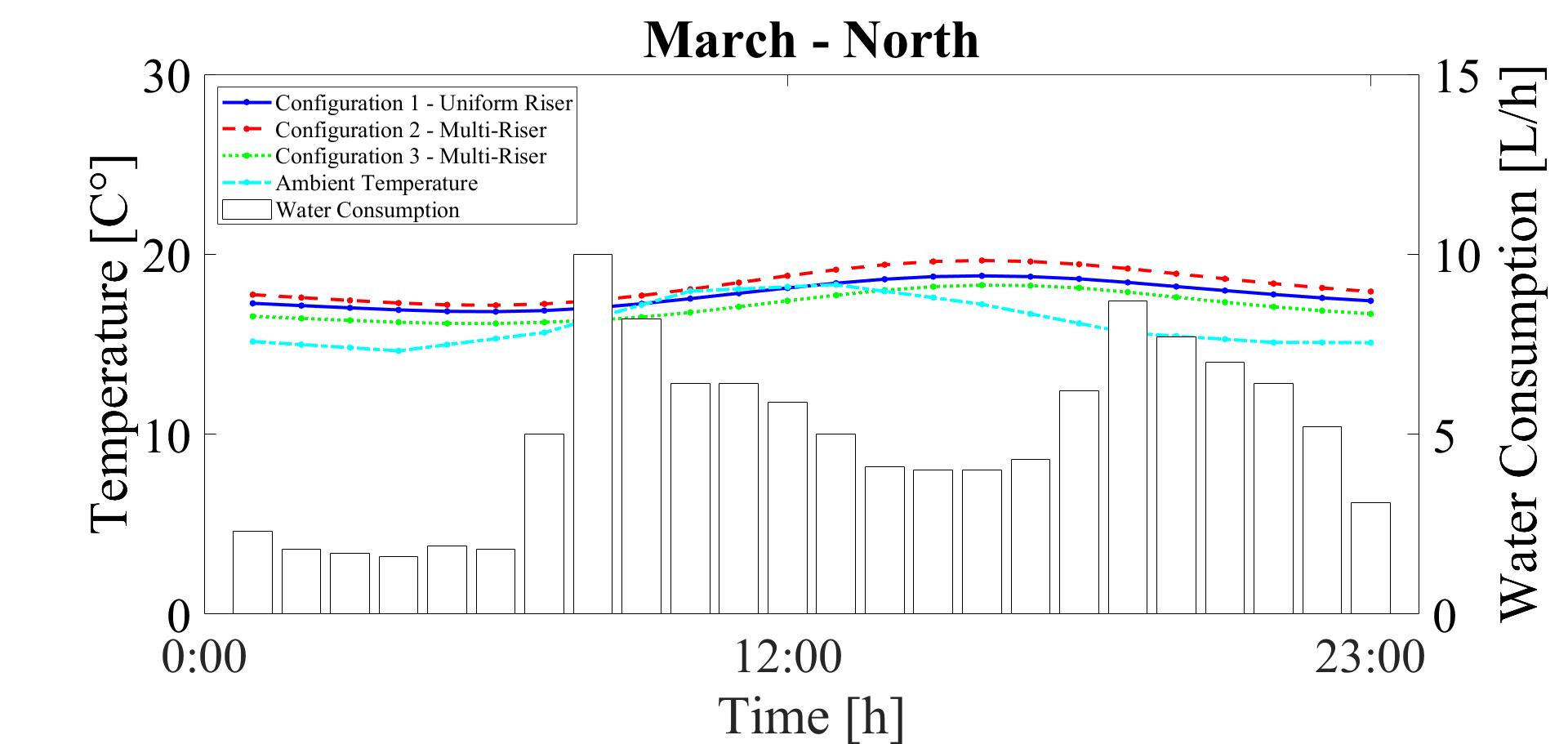 |
| **Graph – March - South – Slope 90°** |
| 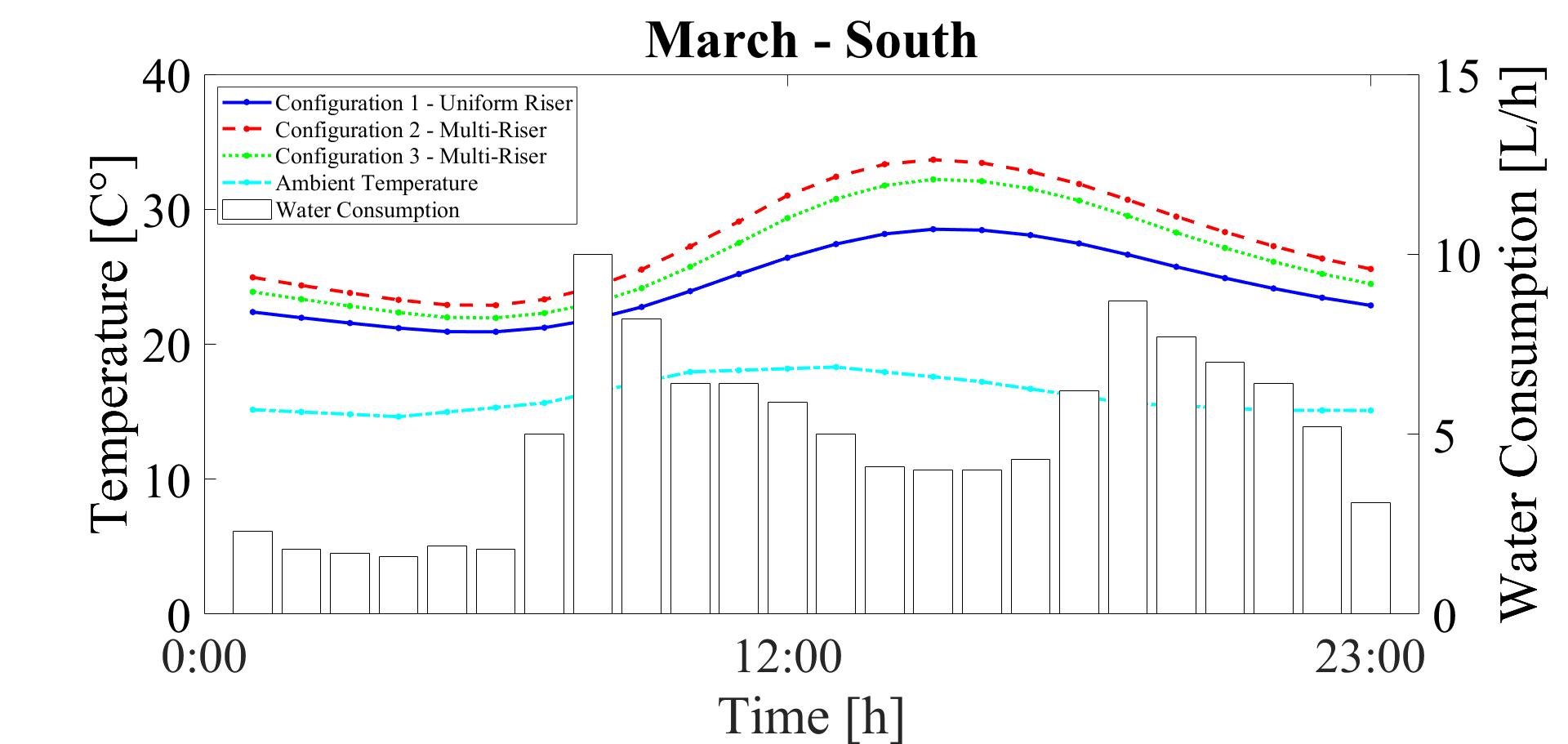 |

**Figure B3-5** Fluid Average Temperature - System comparison - March – North and South – Slope 90°

| **Graph – March - West – Slope 90°** |
| --- |
| 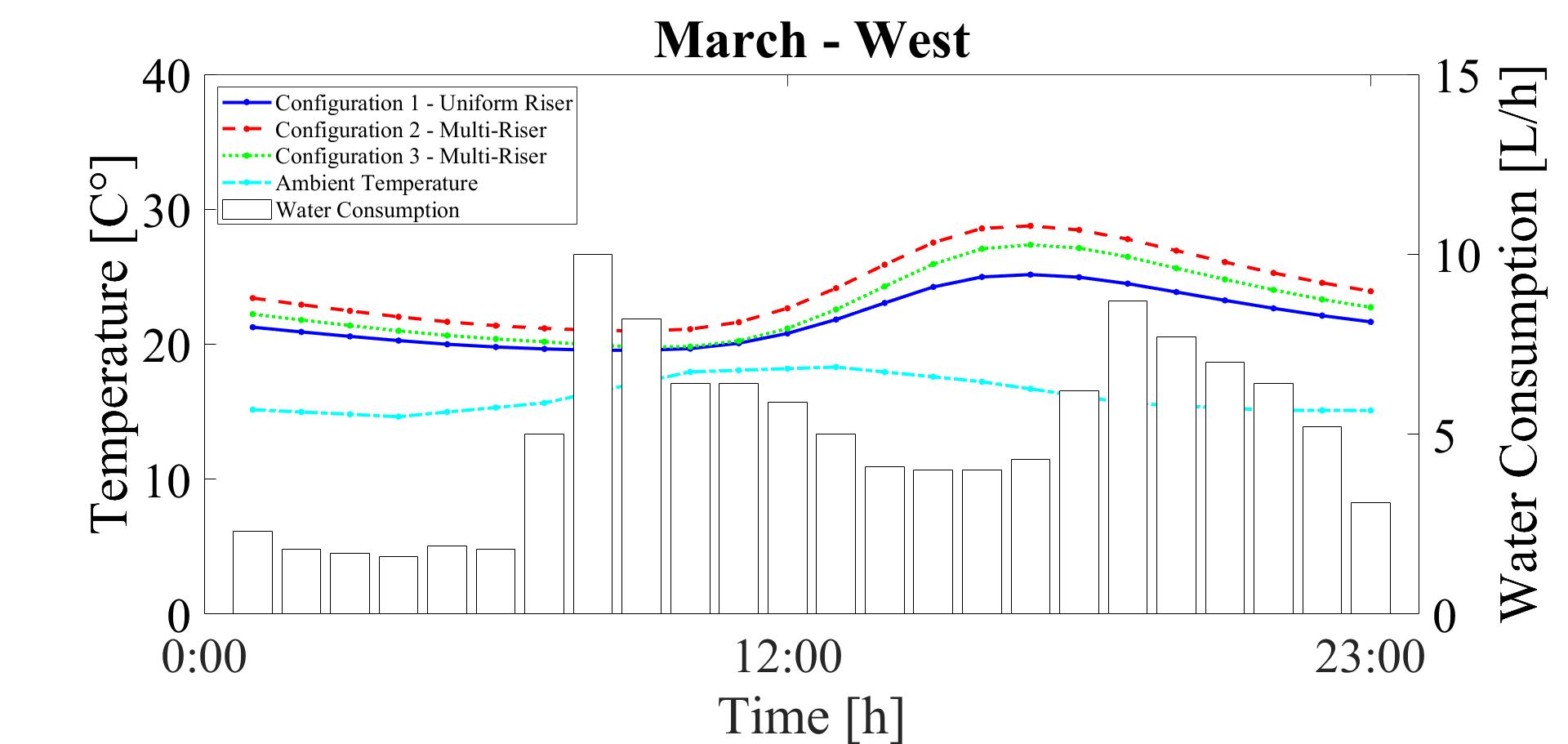 |
| **Graph – March - East – Slope 90°** |
| 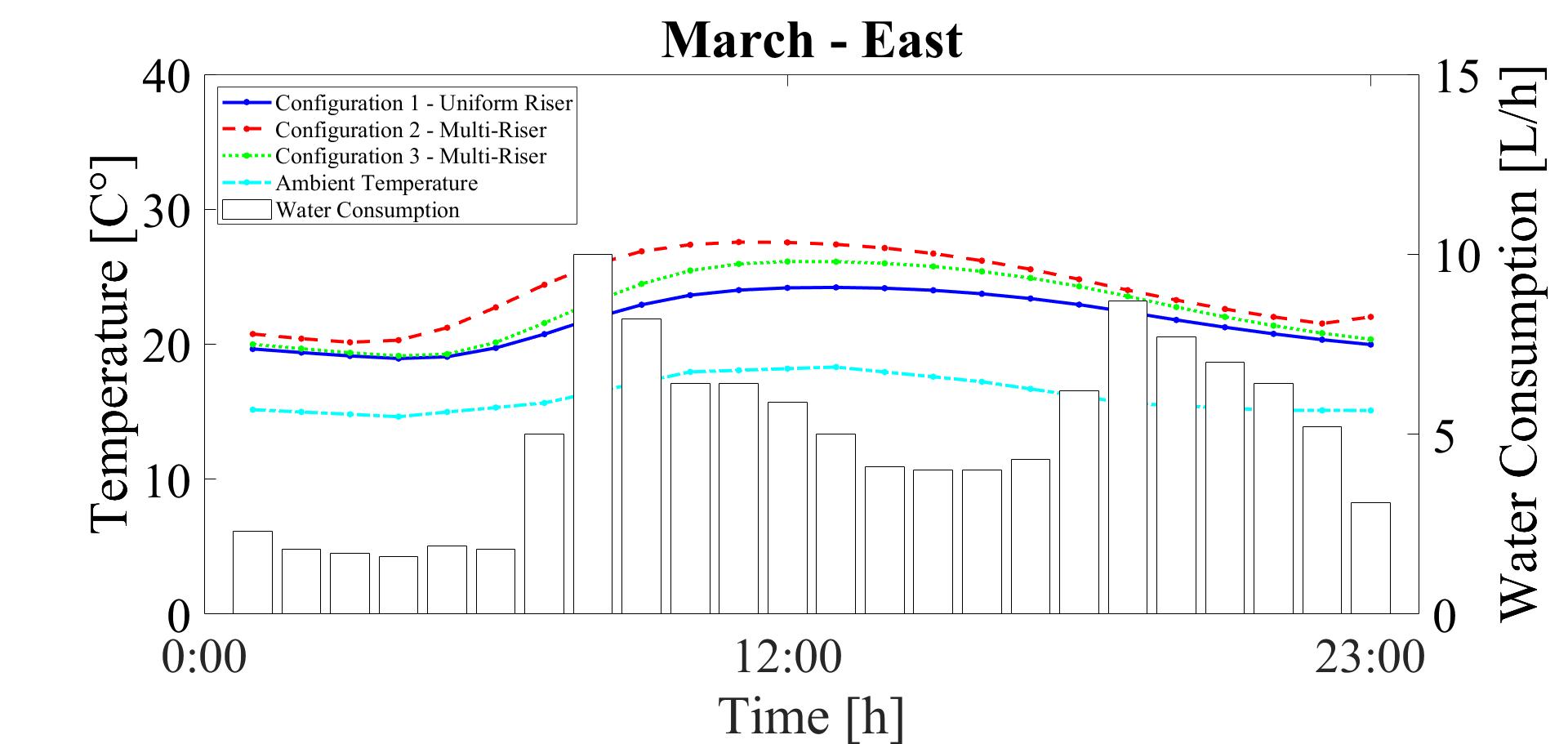 |

**Figure B3-6** Fluid Average Temperature - System comparison - March – West and East – Slope 90°

| **Graph – August - North – Slope 90°** |
| --- |
| 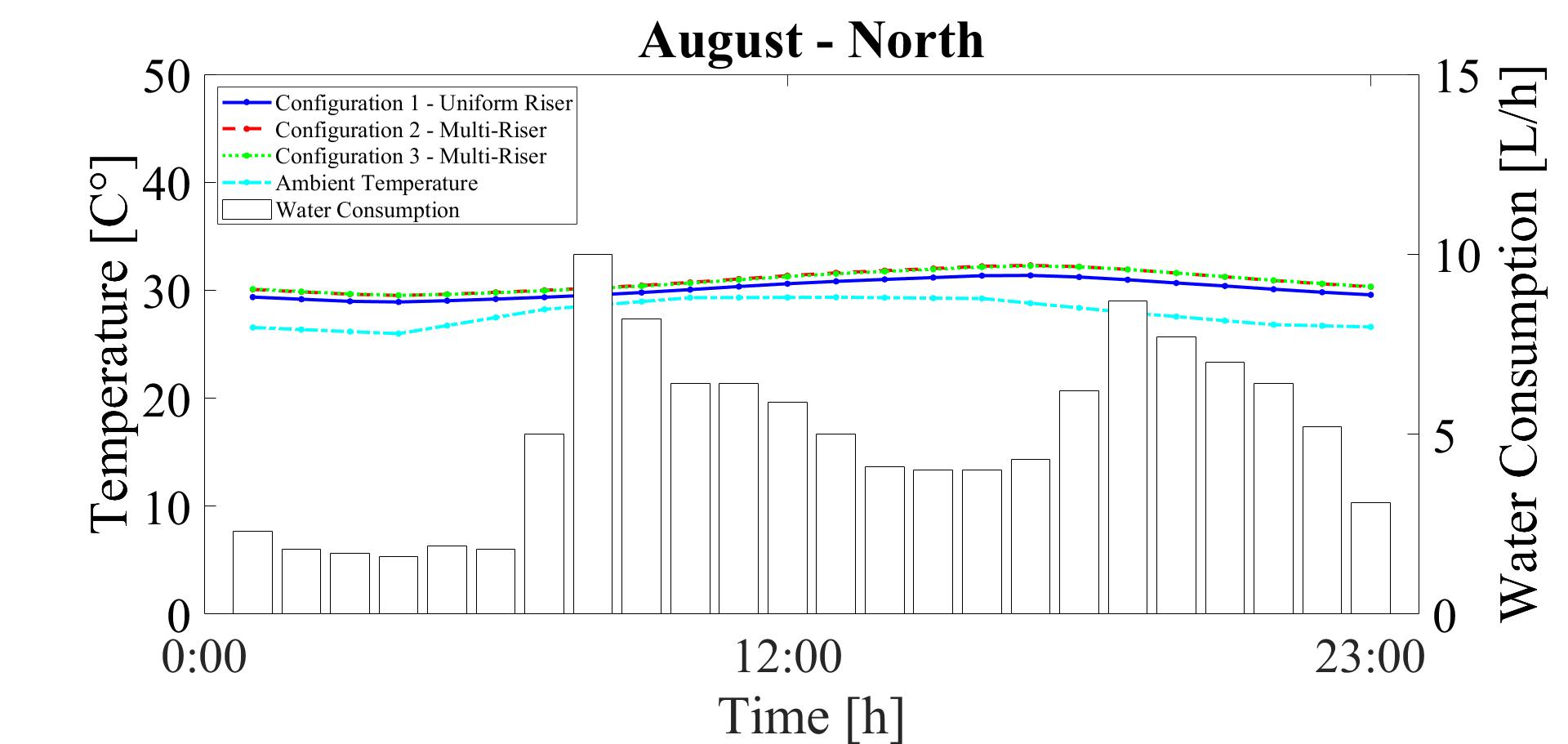 |
| **Graph – August - South – Slope 90°** |
| 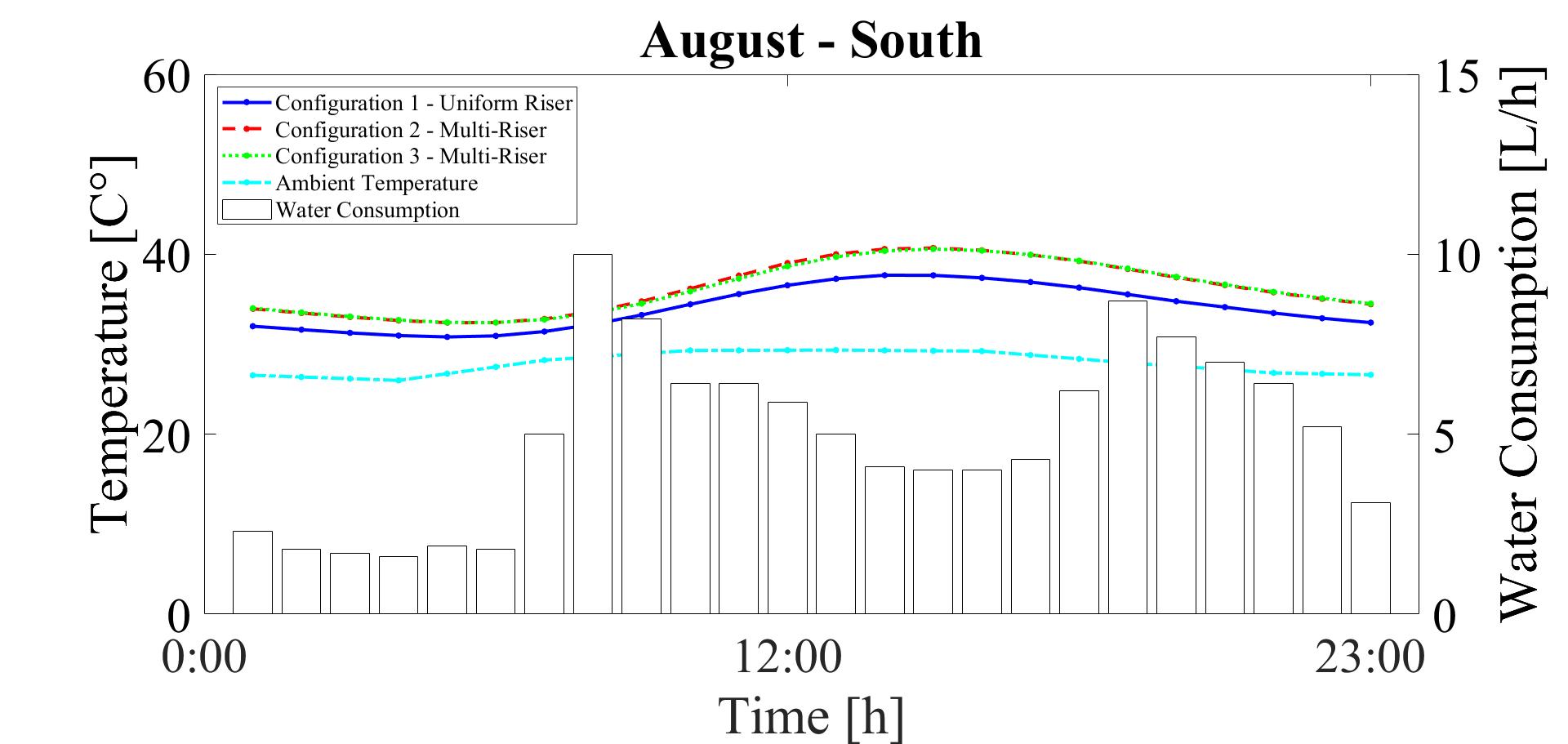 |

**Figure B3-7** Fluid Average Temperature - System comparison - August – North and South – Slope 90°

| **Graph – August - West – Slope 90°** |
| --- |
| 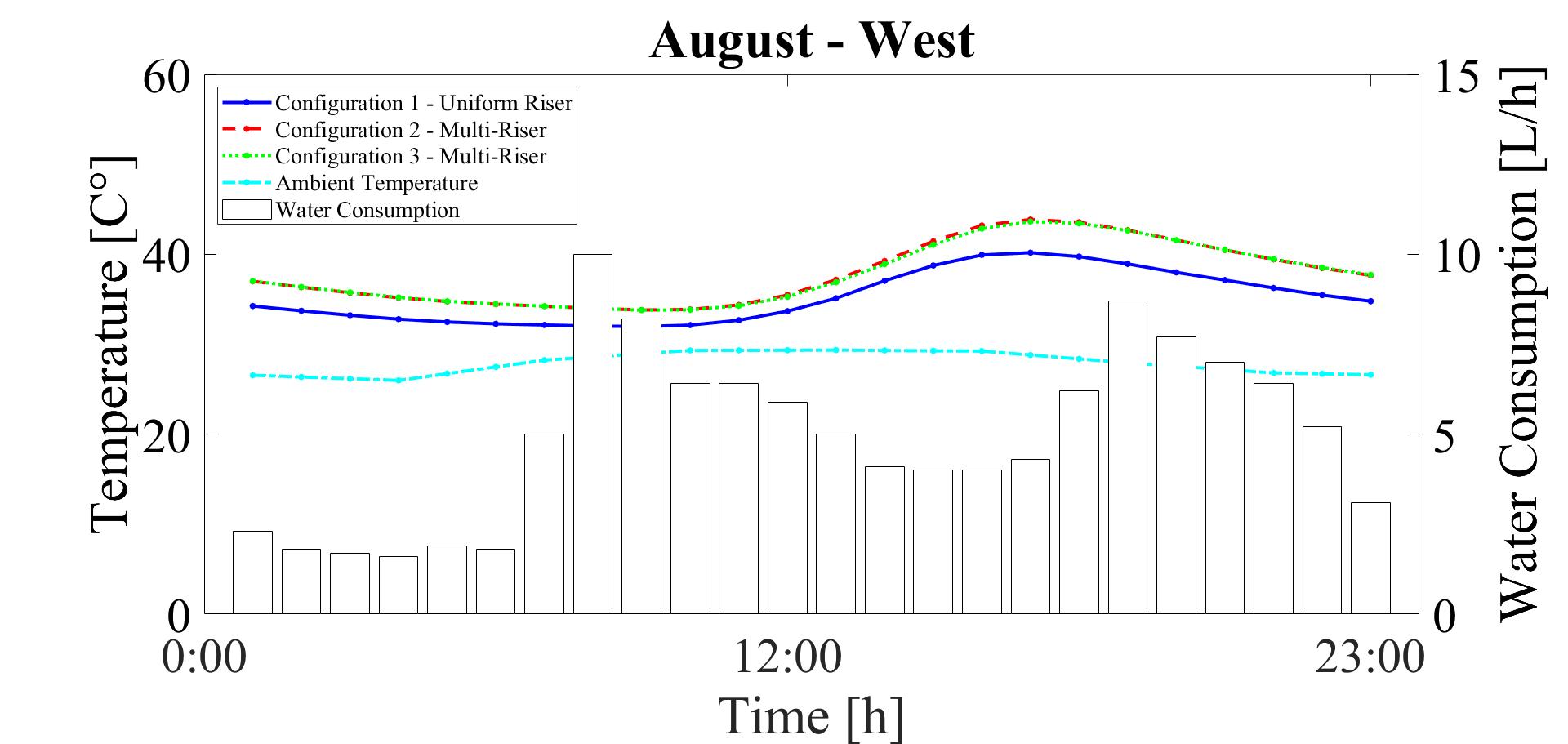 |
| **Graph – August - East – Slope 90°** |
| 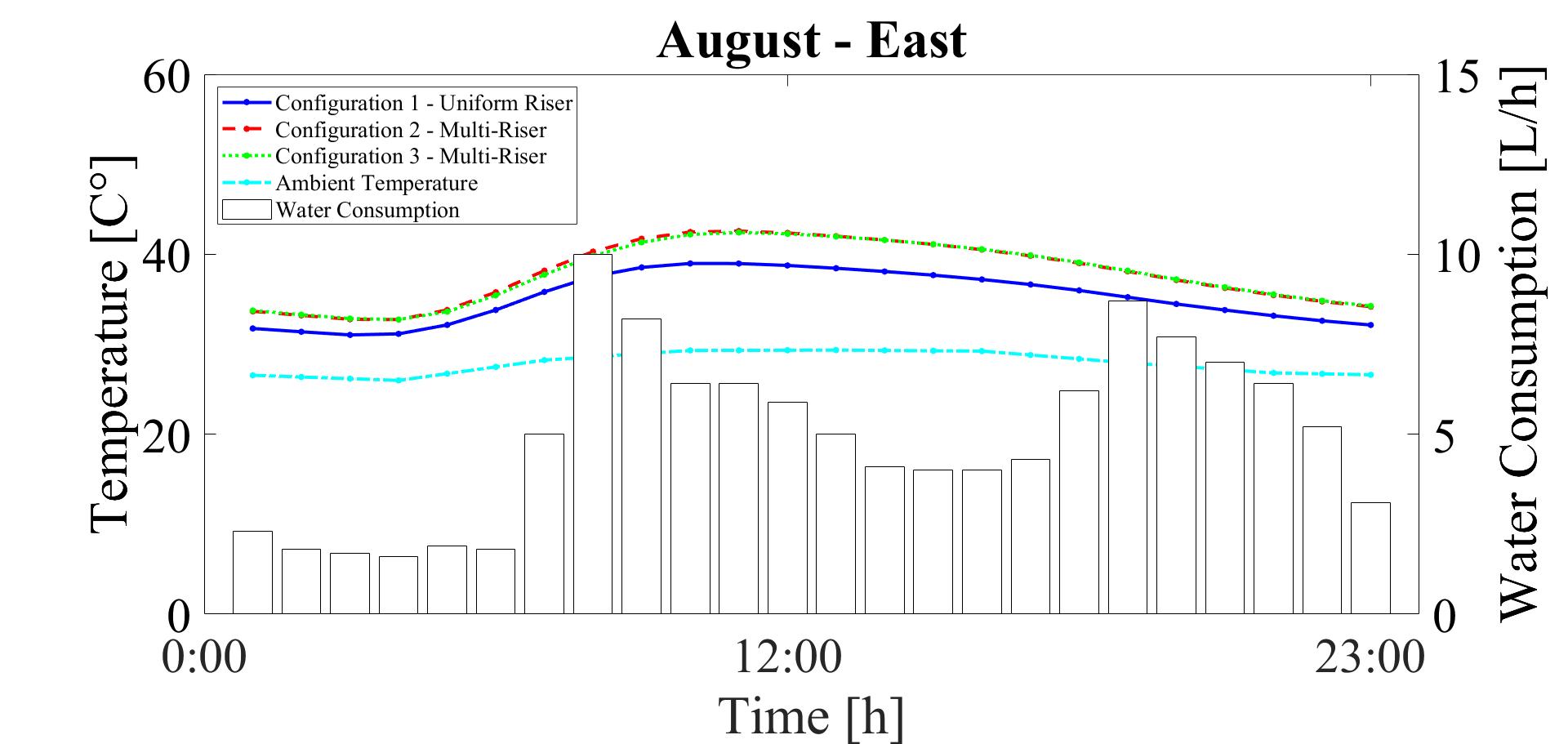 |

**Figure B3-8** Fluid Average Temperature - System comparison - August – West and East – Slope 90°
